# Supplementary material for: Isotopic evidence of high reliance on plant food among Later Stone Age hunter-gatherers at Taforalt, Morocco
Source: Nat Ecol Evol. 2024 Apr 29;8(5):1035–45. doi: 10.1038/s41559-024-02382-z (PMC11090808; doi:10.1038/s41559-024-02382-z)
Supplement: Supplementary file 1 — Supplementary Sections 1–7, Figs. 1–17 and Tables 1–13. [file 41559_2024_2382_MOESM1_ESM.pdf]

nature ecology & evolution

Article

https://doi.org/10.1038/s41559-024-02382-z

Isotopic evidence of high reliance on plant food among Later Stone Age hunter-gatherers at Taforalt, Morocco

In the format provided by the authors and unedited

This PDF file contains:

Supplementary Information 1: Archeological context

Supplementary Information 2: Details on isotope proxies used in this study

Supplementary Information 3: Material and Methods

Supplementary Information 4: Results

Supplementary Information 5: Additional discussion

Supplementary Information 6: Supplementary figures and tables

Supplementary Information 7: 3D models of the human teeth and sampling strategy

1. Supplementary Information 1: Archeological context

1.1 The site of Taforalt "Grotte des Pigeons"

The cave site of Taforalt (also known as La Grotte des Pigeons) is located in eastern Morocco (34° 48' 38" N, 2° 24' 30" W) approximately 500m northeast of the village Tafoughalt from which it derives its name. The site is approximately 40km inland from the Mediterranean Sea and at an altitude of 750m above sea level. The cave is a large dolomitic limestone cavity formed in the Beni Snassen Mountains and opens to Northeastern with 30m in width and 15m in length [1].

The site was first discovered by Dr. Pinchon in 1908, since then the cave was the subject of several excavations. The first excavations were initiated by Ruhlmann from 1944 to 1947 where he made two surveys: in the central and the right lateral part of the cave and he reached the bottom of the fill inside the cavity. His works have provided insight into the nature of the archeological deposits which span the Aterian (Middle Stone Age) and the Iberomaurusian (Later Stone Age) (Roche, 1976). From 1950 to 1955 Roche carried out a systematic excavation of the the ashy layers which he described them as an Iberomaurusian occupation. During his campaign, he uncovered an extensive series of burials in the grey ashy deposits. This includes the two burial areas (designated Necropolis I and II). Moreover, he recovered multiple human graves which were analyzed by Ferembach in 1962. Further excavations were carried out from 1969 to 1979 to acquire detailed information about the archaeological and geological aspects [2]. From 2003 to 2017, new work was conducted on the Iberomaurusian sequence of Taforalt. These new investigations of the Iberomaurusian sequence described two main units[1,3]:

The Yellow Series (YS): The underlying deposit, known as the Yellow series, is associated with the early Iberomaurusian phase, dated around 22,292-21,825 cal BP[1]. This series extends from approximately 22,292 cal BP to around 15,000 cal BP, marking a clear sedimentation change in the Iberomaurusian deposits around 15,190-14,830 cal BP[1].

The Grey Series (GS): The upper unit refers to the grey ashy deposits of the Iberomaurusian occupation with a 4m-thickness and it is dated between 15,000 and 12,600 cal B.P. The sedimentary change between the YS and GS coincides with a change in subsistence strategy by the Iberomaurusians[1]. The recent excavations revealed a series of primary burials in sector 10 in

the grey ashy deposits and evidence show that this area was reused several times because earlier burials were disturbed by subsequent ones.

1.2 The human burials at Taforalt

The recent excavations (2003-2017) revealed a series of additional burials including twelve partially articulated skeletons (seven adults and five infants). This disarticulation indicates the intensive use of the area. In addition to that, an almost complete infant skeleton and a broken cranium alongside a mandible were discovered in the same area. The cemetery mainly includes primary burials which indicate that death happened not too far from the site. Furthermore, there are some secondary depositions of skeletal elements with post-mortem modifications (removal of soft tissues). There are two suggested hypotheses for these secondary burials, either the individuals died elsewhere –which could suggest a seasonal occupation of the site– and some selected parts were carried to the site for burial or these burials simply reflect a change in funerary practices over time[4,5].

However, most of the discovered skeletons were buried in a seated or semi-reclined position. The majority of the individuals were facing towards the entrance of the cave. The bodies were probably surrounded by a pocket of empty space during their decomposition and were covered by organic materials because of the partial loss of anatomical articulation of skeleton elements. It should be mentioned that many burials were associated with funerary objects like horn cores and stones stained with ochre [6].

Seven human bone samples from Sector 10 (GS) have been directly dated by radiocarbon accelerator mass spectrometry (AMS) using ultrafiltration giving dates that span the period of 15,077 cal BP and 13,892 cal BP[4]. Details regarding the human burials and their archaeological context are published in a monograph[2].

1.3 The Iberomaurusian culture

In Northwest Africa, the end of the Late Pleistocene is associated with the Iberomaurusian culture characterized by bladelets and microlithic backed tools, which marks a technological break with the antecedent Aterian (Middle Stone Age)[7,8]. The Iberomaurusian culture first appeared around 25,000 cal BP in North Africa[8] and may have persisted into the Holocene after 11,600 cal BP[9]. The Iberomaurusian culture has covered a vast geographical area stretching from modern day Morocco in the west (10°W) to Cyrenaica in the east (22°E). However, its southern extension is still not well understood but it reaches up to about 33°N along the Atlantic coast. The term of the "Iberomaurusian" was coined by Pallary in 1909. He used this term to refer toan assemblage primarily composed of microlithic tools, which he discovered under the shelter of La Mouillah (Oran region, west Algeria). The second part "maurusian" refers to the Moors which is a Roman name for North African population. Pallary used the term "Ibéromaurusien" to draw attention to the similarities between the lithic industries found in Spain and Morocco. However, the cultural link with southern Europe was later rejected by archaeologists who noted stronger African affinities. Instead they adopted alternative names such as "Oranian" or "Mouillian". Recently some researchers proposed "Late Upper Palaeolithic of Northwest African facies" in order to avoid terminological confusion[10]. However, the scientific community prefers to keep the term of Iberomaurusian as proposed by Camps, 1974[2]. This term has always persisted in the literature and that is why we adopt it here.

1.4 Faunal remains of Taforalt

Several faunal taxa are recovered from the Later Stone Age deposits at Taforalt. The most common taxa are Barbary sheep (*Ammotragus lervia*), equids, large bovines, gazelles and members of the Alcelaphinae subfamily (hartebeest). Barbary sheep was the dominant species of game exploited by the Iberomaurusians at this site[11]. This can be easily explained by the location of the cave which corresponds to its preferred habitat (stony plateaus, steep valley slopes). The rest of the species are typical of open grassland or steppe. Butchery marks on the remains of Barbary sheep indicate it was primarily consumed by this population[11] (Supplementary Figure 1). The presence of Barbary sheep horn cores accompanying human burials in sector 10 suggests their use as funerary objects. The occurrence of these horn cores in both Taforalt's sector 10 and Afalou bou Rhummel[12,13] raises the intriguing possibility of common and synchronous idiosyncratic practices during the Late

Iberomaurusian across varied geographical regions. The study of the faunal assemblage from Taforalt indicates that the Iberomaurusians hunted all age-groups of herbivores but that prime adults were preferentially chosen[11].

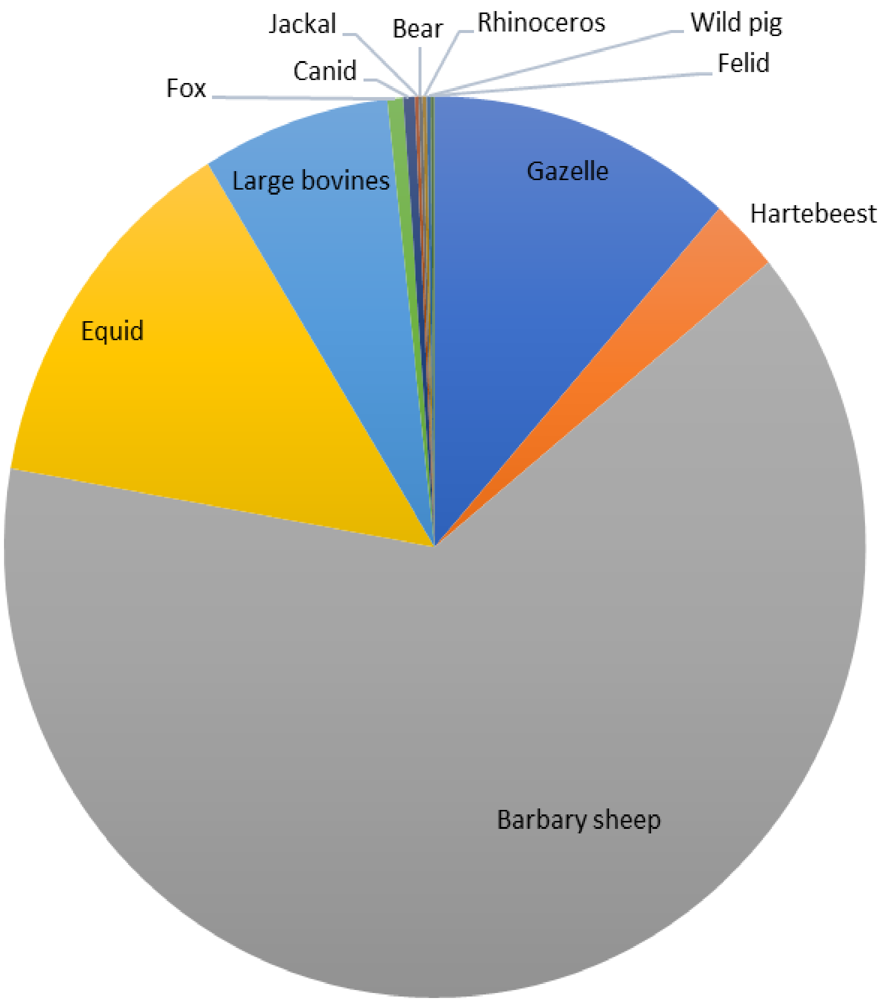

Supplementary Figure 1: The percentage of number of identifiable faunal species from sector 10 at Taforalt[11]

Supplementary Table 1 : List of the identifiable faunal species from sector 10 Taforalt[11,14]. NISP stands for number of identified specimens.

**Faunal remains sector 10**		
**Taxa**	**NISP**	**NISP%**
Barbary sheep (*Ammotragus lervia*)	433	60.39
Equid (Equidae)	91	12.69
Gazelle (*Gazella sp.*)	78	10.88
Large bovines	48	6.69
Hartebeest (*Alcelaphus buselaphus*)	18	2.51
Fox (*Vulpes vulpes*)	4	0.56
Canid (Canidae)	3	0.42
Jackal (*Canis aureus*)	1	0.14
Bear (Ursidae)	1	0.14
Rhinoceros (Rhinocerotidae)	1	0.14
Wild pig (*Sus scrofa*)	1	0.14
Felid (Felidae)	1	0.14
Total	681	95

2 Supplementary Information 2: Details on isotope proxies used in the study

2.1 Carbon and nitrogen isotope ratios of bulk collagen and single amino acids

2.1.1 Carbon and nitrogen isotopes of bulk collagen

Nitrogen isotope ratios ($\delta^{15}N_{collagen}$) reflect the trophic position of an individual in a food web[15–17], as there is typically 3-5‰ increase in $\delta^{15}N_{collagen}$ values between diet and consumer[18]. Carbon isotope ratios ($\delta^{13}C$), on the other hand, provide information on the source of primary production at the base of the food web [16,19]. Carbon and nitrogen isotope analyses from bone proteins therefore provide valuable information on the past diet[17,20–22].

However, collagen is susceptible to diagenetic alterations, which can potentially influence its isotopic composition[23]. To mitigate the impact of such alterations and ensure the integrity of our results, we employ the calculation of C:N ratios (2.9-3.6) as a quality control measure for the collagen, following the approach outlined by Ambrose in 1990[24]. This robust quality control step is essential in assessing the reliability of our collagen samples in the face of potential diagenetic influences on isotopic data.

Reconstructing the paleodiet using the bulk collagen has three major limits:

1) The utilisation of bulk collagen $\delta^{13}C_{collagen}$ to reconstruct a marine and terrestrial diet is often limited in regions with mixed C3 and C4 environments. This is because of the overlap in $\delta^{13}C_{collagen}$ values of C4 plants and high marine proteins consumers, both having relatively more positive values. Freshwater fish and terrestrial fauna can also exhibit similar $\delta^{13}C_{collagen}$ values. Previous studies showed the limitation of estimating the marine food contribution in the diet of humans living in regions with mixed C3/C4 terrestrial environments[25,26].
2) Bulk collagen $\delta^{15}N_{collagen}$ isotope ratios are affected by several environmental and physiological factors in addition to the trophic level effect. For instance, elevated $\delta^{15}N_{collagen}$ values of herbivore tissues are documented in arid regions, and consequently lead to a misinterpretation of the trophic levels of humans [27,28]. In addition, there is a strong baseline effect impacting the $\delta^{15}N_{collagen}$ and $\delta^{13}C_{collagen}$ values of animals with similar diets,

but coming from different regions. This is due to environmental factors, such as the aridity, influencing the isotope composition of plants. It is often assumed that there is a 3.4‰ increase per trophic level[29] but already Deniro and Epstein, 1981[29] observed a wide variation in $\delta^{15}N_{collagen}$ trophic discrimination factors depending on the samples analysed (-0.5 to +9.2)[29].

3) Another problem is related to the characterization of $\delta^{15}N_{collagen}$ in the primary producers, like plants, which can display an important temporal and geographical variation, possibly because of the assimilation of nitrogen from different sources [30]. For example, plants that fix nitrogen from the soil (NH_4^+, NO_3^-) can exhibit distinct nitrogen isotopic signatures compared to those that fix nitrogen from the atmosphere (N_2)[30]. These differences in isotopic ratios are influenced not only by nitrogen sources but also by various location-specific environmental characteristics, such as precipitation, altitude, temperature, and salinity[31,32].

2.1.2 Carbon and nitrogen on single amino acids

Compound Specific Isotope Analysis of Amino Acids (CSIA-AA) has recently been used to overcome the methodological problems of bulk isotope analyses[33]. The use of this powerful new technique allows estimating more precisely the trophic position of organisms and eliminating the influence of environmental factors. The analyses of $\delta^{13}C$ isotope value of Single Amino Acid (SAA) (Phe: Phenylalaline, Val: Valine*)* can clearly differentiate terrestrial from freshwater food resources while N isotope on SAA (Phe: Phenylalaline, Glu: Glutamic acid) elucidate the trophic position with more precision than possible with the bulk isotope analyses[34,35].

2.1.2.1 Trophic position estimation using $\delta^{15}N$ of amino acids

One could estimate the Trophic Position (TP) of humans without need to characterize the $\delta^{15}N$ values of the associated fauna by analysing nitrogen isotope ratios of single amino acids, particularly the Phenylalanine (Phe) and the Glutamic acid (Glu). These amino acids serve as excellent tracers of the trophic level. The $\delta^{15}N_{Glu}$ is significantly enriched for each trophic position (+8.00‰) because of the substantial isotopic fractionation occurring during the transamination. The $\delta^{15}N_{Phe}$ shows only a slight change with each trophic position (+0.4‰) and mainly reflects the

local base line, which in turn influences the $\delta^{15}N_{Glu}$ with the same amplitude. By combining the analysis of these two amino acids, it becomes possible to determine the trophic position of an organism while effectively eliminating interference from the baseline effect [34].

The analysis of $\delta^{15}N$ on these two amino acids provides an internal trophic position (TP) indicator which can be calculated by employing the following equation[34]:

$$TP(C3) = [(\Delta^{15}N_{Glu\text{-}Phe} + 8.4)/7.6] + 1$$

2.1.2.2 Carbon isotope ratios on amino acids and exploited environment

Previous studies[35,36] demonstrated the potential of carbon isotope analyses on the Phe and the valine (Val) to distinguish between 4 main dietary groups (Marine fish consumers; freshwater fish consumers, terrestrial C_3 protein consumers, terrestrial C_4 protein consumers). Generally, terrestrial C_3 protein consumers have similar $\delta^{13}C_{Phe}$ and $\delta^{13}C_{Val}$ values in collagen. The terrestrial C_4 protein consumers would also exhibit similar $\delta^{13}C_{Phe}$ and $\delta^{13}C_{Val}$ values but relatively higher. The $\delta^{13}C_{Val}$ values of freshwater fish consumers overlap with the terrestrial C_3 protein consumers but have a lower $\delta^{13}C_{Phe}$. The $\delta^{13}C_{Val}$ of marine protein consumers overlap with the terrestrial C_4 protein consumers but have lower $\delta^{13}C_{Phe\ values}$ [35,36].

2.2 Zinc isotope ratios: a dietary proxy retrieved from dental enamel

Dental enamel is more resistant to time degradation than bone, therefore, dietary investigations of the new metal isotope trophic proxies in the mineral hydroxyapatite $Ca_{10}(PO_4)_6(OH)_2$ have gained increased attention in recent years[37–42]. The Zn isotopic composition of vertebrate tissues appears to be strongly related to diet[38,43–46]and can be analysed in dental enamel(oid), a tissue very resistant to diagenesis[37–39,43,47].

The variability of the $^{66}Zn/^{64}Zn$ ratio (expressed as $\delta^{66}Zn$ value) in vertebrate consumer is caused by two main factors. The Zn isotope composition from the source of intake and the Zn isotope fractionation within the organism. In the food web, the Zn isotope composition of primary producers, like plants, comes from the underlying bedrock further fractionated within the plants tissues. However, bedrock $\delta^{66}Zn$ values of this latter can exhibit some variations depending on

their composition[38,48]. For instance, marine carbonates (+0.3 to +1.4‰) can display higher and much more varied values than most clastic and volcanic rocks (+0.3±0.14‰ [2σ]) and other sedimentary rocks[49–51].

Previous work on bioapatite δ^{66}Zn values have shown a ^{66}Zn enrichment in herbivore's enamel bioapatite relative to that of carnivores. This is because zinc isotope compositions become relatively depleted in heavy isotopes with successive trophic levels[44]. Therefore, herbivores have systematically higher δ^{66}Zn values than carnivores ($\delta^{66}Zn_{herbivore} > \delta^{66}Zn_{carnivore}$), which is consistent with previous findings demonstrating a decrease of approximately 0.30 to 0.60‰ in δ^{66}Zn values with each step in archaeological and modern food webs[38,46,52,53].

The trophic fractionation of δ^{66}Zn is a result of Zn isotope fractionation within an organism[54–57]. The body muscles are usually depleted in heavy Zn isotopes compared to the diet and bioapatite tissues[54,56]. Thus carnivores, which consume exclusively meat, are expected to exhibit lower bioapatite zinc isotope ratios compared to those of their prey. Because bones are enriched in heavy Zn isotopes compared to muscles, bone consumers like hyena usually exhibit a different isotopic composition than sympatric carnivores feeding exclusively on meat [46]. Zinc isotope fractionation also occurs in plants resulting in a different isotopic composition among roots, stems and leaves[58,59]. This isotopic variation within a plant may affect the entire trophic web. Some Zn studies showed that faunal species feeding on leaves ("browsers") have different δ^{66}Zn values than those feeding on grass ("grazers")[45,46]. Leaves are usually ^{64}Zn-enriched compared to the other parts of the plants. Consequently, browsers exhibit lower Zn isotopic ratios than grazers[46,59]. However, this browser-grazer δ^{66}Zn variability may not be ubiquitous, as it has not been observed in South East Asian tropical food webs[38,43].

One study documented the impact of breastfeeding on $\delta^{66}Zn_{enamel}$ values with teeth formed during breastfeeding having higher values than post-weaning formed enamel[48].

In summary, meat consumption is associated with low δ^{66}Zn values – especially when combined with elevated δ^{15}N values in bone collagen. In contrast to N, Zn becomes relatively depleted in heavy isotopes with successive trophic levels. However, both N and Zn show higher isotope ratios in tissues of breastfed children relative to their nurturer[48,60].

2.3 Strontium isotope ratios: a mobility proxy retrieved from dental enamel

Strontium, a trace element, consists of four naturally occurring isotopes, including three stable ones: ^{84}Sr, ^{86}Sr, and ^{88}Sr. The fourth isotope, ^{87}Sr, is radiogenic and is produced through the radioactive decay of rubidium, specifically ^{87}Rb, which has a half-life of approximately 4.7×10^{10} years. The natural abundances of these isotopes are as follows: ^{84}Sr (0.56%), ^{86}Sr (9.879%), ^{88}Sr (82.53%), and ^{87}Sr (7.04%). The amount of radiogenic ^{87}Sr in geological formation depends on factors like rock age and the ^{87}Rb/^{87}Sr ratio, causing variations in the ^{87}Rb/^{87}Sr ratio in bedrock based on rock age and lithology[61,62].

The isotopic composition of strontium is expressed as the ratio of ^{87}Sr/ ^{86}Sr. Generally, the ^{87}Sr/ ^{86}Sr ratio of the continental crust varies between 0.700-0.750[62]. Rocks with expected high original ^{87}Rb/^{87}Sr ratio will exhibit a higher ^{87}Sr/^{86}Sr ratio, usually above 0.710. This often includes lithologies such as granite and gneiss. Lithologies with low ^{87}Rb/^{87}Sr usually have low ^{87}Sr/ ^{86}Sr ratio (0.706) often even lower in basalts (0.704)[61]. On the other hand, unaltered marine limestones and dolomites exhibit an intermediate Sr signature (0.707-0.709) recording the isotopic composition of the seawater at the time of carbonate mineral precipitation. Although the isotopic variations seem small, they are large compared to the instrumental error of modern mass spectrometers (± 0.00001)[61].

Strontium is chemically and structurally similar to calcium. Consequentially, Sr substitutes for Ca in carbonate minerals such as calcite, aragonite and dolomite, as well as in bioapatite[63]. Strontium is released from rocks by weathering and incorporated into the soil, ground water and stream, which will be taken up by primarily producers and then passed on through the food web. The fractionation of ^{87}Sr/^{86}Sr is small due to its large isotopic mass and invisible due to the normalization performed during Sr isotope analyses[61]. Therefore, the Sr signature within an organism reflects that of local geology. For humans and other animals, strontium is incorporated into their skeleton via diet. Thus, bioapatite ^{87}Sr/^{86}Sr ratios serve as a tracer for the location of an individual's dietary resources.

2.4 Sulphur isotope ratios: a mobility proxy retrieved from bone and tooth collagen

Sulphur (S) isotope analysis is a complementary method to C and N isotope analyses, a tool for gaining insights into mobility and the consumption of marine foods [64,65].

On earth, there are three main reservoirs for S: Oceanic dissolved sulphates, evaporitic sulphates and pyrite[65]. Sulphur analysis is primarily based on the variations in δ^{34}S values observed across different ecosystems, such as marine, freshwater, and terrestrial environments. These variations serve as a fundamental principle for studying sulphur isotopes. In marine ecosystems, the δ^{34}S values are close to +20‰, the value of the oceanic sulphates[66]. Individuals living close to coastal regions display δ^{34}S values close to +20‰ due to the “sea spray effect”. Terrestrial plants, representing the base of a trophic chain receive their S through soil minerals or from aerosols. The aerosols are the main sources of sulphur for plants living close to coastal regions[67].

Meanwhile, terrestrial δ^{34}S values vary from ca. -20‰ to +20‰ and depending on the S of the local bedrock[65,68]. Sulphur is released from rocks by weathering and incorporated into the soil, which will be taken up by primarily producers and then passed on through the food web. For instance, low δ^{34}S values are found in sedimentary sulphides and igneous minerals, while the values largely increase in geological sulphates[67].

The plants are depleted by 1.5‰ to their sources of sulphates[69]. A small number of publications shows that the isotopic fractionation between diet and consumers tissues, is likely small to negligible[65]. Therefore, the δ^{34}S values reflect the local ecosystem of the individual[70]. Combined δ^{34}S with δ^{13}C and δ^{15}N values enables the detection of seafood consumption[69–72].

3 Supplementary Information 3: Material and Methods

3.1 Faunal samples

Archaeological samples included in this study originated from the Taforalt archaeological site in Morocco, and the necessary permits for exporting and sampling human and faunal specimens were obtained from the Institut National des Sciences de l'Archéologie et du Patrimoine (INSAP) in Rabat, Morocco, under permit N°02/2019-2020-232. The analyses were conducted at the Max Planck Institute for Evolutionary Anthropology in Leipzig.

3.1.1 Faunal species identification

Only a small number of carnivore remains were available at the site (NISP= 9, Supplementary Table 1) and we sampled all teeth available for the isotopic analysis (n=2). However, these carnivores might not have a pure carnivorous diet, since many canid species can feed on a variable amount of plants[73].

One carnivore (SEVA 35834, Supplementary Table 2) was identified by ZooMS as a Canidae (although the identification and combination of peptide markers for this sample exclude the possibility of a red fox (*Vulpes vulpes*, Supplementary Table 2). This means that this specimen more likely belongs to the Caninae subfamily. Another carnivore (SEVA 35828) was identified using traditional zooarchaeology as a red fox (*Vulpes vulpes*)[11].

The Barbary sheep samples were initially identified using traditional zooarchaeological methods. Of the samples, three were subsequently confirmed as Caprinae through ZooMS analysis, which aligns with the initial zooarchaeological identification.

ZooMS analysis corroborated the zooarchaeological identification of equids, hartebeest, Rhinoceros, and gazelle. Moreover, it provided a more specific identification for a bovid, which was determined to be a hartebeest. However, one Barbary sheep sample and hare proved unidentifiable through ZooMS analysis. Consequently, we retained the initial zooarchaeological identification for these specimens in our study (Supplementary Table 2). Among the herbivore taxa, most of them are expected to be grazers but Barbary sheep and gazelles can be mixed-feeders[74,75]. Gazelles, hartebeest and Barbary sheep are also drought tolerant[76–78].

Supplementary Table 2: The morphological and ZooMS taxonomic identification of the faunal remains included in this study. SEVA numbers are the identification numbers given to each sample processed and analysed at the Max Planck Institute for Evolutionary Anthropology.

ID	Sector	SEVA	Zooarchaeological identification	Tissue type	ZooMS	Final identification	Latin name
TAF 10-11408		34565	Equus	M		Equus	*Equidae*
TAF 05-3475	sector 10	34566	Barbary sheep	M		Barbary sheep	*Ammotragus lervia*
TAF.13-12280	sector 10	35819	Barbary sheep	M3	Unidentifiable	Barbary sheep	*Ammotragus lervia*
TAF.04-379	sector 8	35820	Equus	M1/2	Equidae	Equus	*Equidae*
TAF.13-11819	sector 10	35821	Barbary sheep	dm3	Caprinae (Ovis)	Barbary sheep	*Ammotragus lervia*
TAF.13-11617	sector 10	35822	Equus	I	Equidae	Equus	*Equidae*
TAF 05-2907	sector 10	35823	Hare	bone	Unidentifiable	Hare	*Lepus*
TAF-1807		35824	Bovid	M3	Alcelaphinae	Hartebeest	*Alcelaphus buselaphus*
TAF.04-2188		35825	Equus	M3	Equidae	Equus	*Equidae*
TAF 13 -12127	sector 10	35826	Equus	dp	Equidae	Equus	*Equidae*
TAF 08-F23	sector 4	35827	Alcelaphus	M2	Alcelaphinae	Hartebeest	*Alcelaphus buselaphus*

TAF 05-2498	sector 10	35828	Fox	M1	Unidentifiable	Fox	*Vulpes vulpes*
TAF 05-2649	sector 10	35829	Gazelle	M3	Bovidae (not Bovinae or Caprinae) / Cervinae / Giraffidae	Gazelle	*Gazella sp.*
TAF 13-11933	sector 10	35830	Barbary sheep	I	Caprinae (Ovis)	Barbary sheep	*Ammotragus lervia*
TAF 13-11757	sector 10	35831	Barbary sheep	M2	Caprinae (Ovis)	Barbary sheep	*Ammotragus lervia*
TAF 13-11660	sector 10	35832	Gazelle	P2/P3		Gazelle	*Gazella sp.*
TAF 05-2450	sector 10	35833	Gazelle	P	Antilopinae	Gazelle	*Gazella sp.*
TAF 10-11226	sector 10	35834	Canid	M	Canidae (not red fox)	Canid	*Canidae*
TAF 04-1842	sector 8	35835	Gazelle	P2		Gazelle	*Gazella sp.*
TAF 04-375	sector 8	35836	Rhinoceros	P or M	Rhinocerotidae	Rhinoceros	*Rhinocerotidae*

332

3.2 Human material and tooth pathologies

The human samples consisted of 23 teeth and seven bones. Most of the samples belong to 7 individuals buried in sector 10[5]. The other samples were isolated and are therefore described as "unassigned" (Supplementary Table 3). The human teeth were scanned using a micro-CT scanner in the Department of Human Evolution at MPI-EVA (Leipzig, Germany). The teeth were scanned and revealed an important amount of hypoplasia (78% of our samples; n=23) and caries (13% of our samples; n=23). All 3D scans are not available online but are shared by the authors after request. The location of the caries and hypoplasia are documented in the Supplementary Information 7 and are summarized in Supplementary Table 4. Areas sampled for Sr and Zn isotope measurement are also shown in Supplementary Information 7 (Teeth scans).
The sampling strategy is described in detail in section 3.3.1.1, titled "Strategy of Tooth Sampling". The approximate areas that were sampled in the vertical plane are represented by arrows on the tooth scans (Supplementary Information 7). However, it is crucial to note that these arrows provide a general indication of the sampled regions and do not accurately represent the depth or width of the sampled areas.

In this study, our analysis of dental remains has revealed a presence of dentine and enamel stress in the teeth, as indicated in Supplementary Information 4. This finding suggests that periods of tooth formation were marked by disruptions, which can be associated with various stressors experienced during different stages of life[79,80]. In other studies, linear enamel hypoplasia, is typically observed in teeth formed early in life, such as incisors and canines, which coincides with the weaning process[66,67]. Weaning, occurring between 2-5 years of age in hunter-gatherer societies, is often regarded as a period of significant stress for infants[79,81].

However, our investigation has unveiled a more nuanced picture. Notably, hypoplasia is not present solely in teeth formed during the weaning period. We find its presence in teeth that form after weaning, including the M2 and M3 molars. This raises the possibility that factors beyond weaning, such as persistent nutritional stress or traumatic events, may also contribute to the formation of these stress markers. Other influential elements could include genetic predisposition, environmental influences, or psychological stress[82]. Compared to the Natufian population, the Iberomaurusians from Taforalt exhibit a much higher frequency of caries[4,83]. The presence of enamel hypoplasia has been attested in Natufian sites like Nahal Oren in the Levant, mainly

between the age of 3-7yrs[83]. However, we could not establish the frequency of this stress between Natufians and our samples due to our limited sample size.

Supplementary Table 3: The human samples from the sector 10 of Taforalt. analyzed in this study with their sex and age of death estimation (from Humphrey et al., 2020)[5]. SEVA are the identification numbers used at the Max Planck Institute for Evolutionary Anthropology. The ID are the identification numbers given to the finds during the excavations. ND stands for Not Determined. Mo stands for months.

Individual	SEVA	ID	Element	Sex	Age at death
1	35961	TAF_06_3966	M2	male	20yrs
1	35962	TAF_06_4164	M2	male	20yrs
1	35967	TAF_06_3966	I2	male	20yrs
1	35970	TAF_06_3967	M	male	20yrs
1	35979	TAF_06_4167	P4	male	20yrs
1	35985	TAF_06_H576	bone	male	20yrs
1	35963.A	TAF_06_4164	C	male	20yrs
1	35963.B	TAF_06_4164	M2	male	20yrs
2	35981	TAF_05_3281	bone	ND	ND
5	35977	TAF_08_6875	M2	female	16-18yrs
5	35978	TAF_08_6875	C	female	16-18yrs
5	35971.A	TAF_08_6880	I1	female	16-18yrs
5	35971.B	TAF_08_6880	M1	female	16-18yrs
6	35964	TAF_06_4753	dm2	male	6-12mo
6	35965	TAF_06_4753	di1	male	6-12mo
6	35982	TAF_08_5883	bone	male	6-12mo
6	35983	TAF_08_5753	bone	male	6-12mo
9	35966	TAF_09_8111	di1	female	5-6mo
9	35966	TAF_09_8111	bone	female	5-6mo
13	35975	TAF_13_1000	M3	male	18-20yrs
13	35975	TAF_13_1000	bone	male	18-20yrs

14	35980	TAF_10_10105	P4	male	18-20yrs
unassigned	35959	TAF_05_2882	bone	ND	ND
unassigned	35959	TAF_05_2882	I	ND	ND
unassigned	35960	TAF_05_3380	I2	ND	ND
unassigned	35968	TAF_04_1031	M3	ND	ND
unassigned	35969	TAF_05_3685	I2	ND	ND
unassigned	35972	TAF_06_4224	P3	ND	ND
unassigned	35973	TAF_05_3144	I2	ND	ND
unassigned	35974	TAF_06_3907	M3	ND	ND
unassigned	35976	TAF_05_3613	M	ND	ND
unassigned	34561	TAF_10_11025	I1	ND	ND

Supplementary Table 4: Caries and hypoplasia presence in the human teeth from Taforalt included in this study. Hypoplasia are detected on the CT scans (3D models and 2D virtual sections in Supplementary 7). We utilized the positioning of hypoplasia within the teeth in relation to the tooth's developmental timeline[84] to make an approximate estimation of its occurrence period

Individual	sex	age of death	SEVA	tooth	presence of caries	presence of hypoplasia	location	period of hypoplasia/ stress
1	male	20yrs	35963.B	LLM2	yes	yes	complete tooth	3-16yrs
			35961	ULM2	no	yes	complete tooth	3.5-16yrs
			35963.A	LLC	no	yes	complete tooth	4-14yrs
			35962	LLM2	yes	yes	root	7-15yrs
			35970	UxM	no	yes	crown	?
			35967	LRI2	no	yes	complete tooth	3-10yrs
5	female	16-18yrs	35971.A	LLI1	no	yes	complete tooth	small hypoplasia around 3yrs and again at around 5yrs
			35971.B	LRM1	no	yes	crown enamel	between 2.5 and 5yrs
			35978	ULC	no	no	crown	light EH around 3.5yrs
6	male	6-12mo	35965	URdi1	no	yes	root dentine	4.5-11mo
			35964	URdm2	no	no		

9	female	5-6mo	35966	ULM2	no	no		
13	male	18-20mo	35975	LRM3	no	yes	crown enamel	around 13-14yrs
14	male	18-20yrs	35980	LRP4	no	yes	complete tooth	4-10yrs
unassigned			35959	LxI	no	yes	root	3-11yrs
unassigned			35960	llI2	no	yes	complete tooth	strong EH 3-5yrs + slight stress between 5 and 10yrs
unassigned			35968	ULM3	no	yes	complete tooth	14-16yrs
unassigned			35969	LRI2	no	yes	root	7-10yrs
unassigned			35972	LLP3	no	yes	root	6-10yrs
unassigned			35973	LRI2	no	no		
unassigned			35974	ULM3	yes	yes	crown and root	12 yrs-
unassigned			35976	LRM	no	yes	crown and root	?
unassigned			34561	UI1				

3.3 Methods of isotope analyses

The isotopic analyses were conducted in the Max Planck Institute for Evolutionary Anthropology, Leipzig, Germany.

3.3.1 Human Teeth Sampling Approach

3.3.1.1 Estimation of the period of teeth formation

To estimate the age of the individual at the time when the teeth was formed, we referred to AlQahtani, 2010[84]. This reference provided valuable information for determining the timing of tooth development. More specific details are found in the accompanying Excel document (Supplementary table 16). It is important to note that the formation of a tooth occurs over an extended period, for instance, I2 mineralizes between 1 and 10yrs. To account for this variability, we categorized the sampled tooth parts into groups with distinct period of formation groups (Supplementary table 16, Excel document):

- Placental Formation: This category includes tooth parts formed before birth, such as the upper part of the crown of dm2 and di1.
- Pre-Weaning Formation: Tooth parts formed during the first four years of life fall into this category.
- Post-Weaning Formation: Any tooth parts formed after four years are considered post-weaning.

Following this categorization, we exclusively considered values from tooth parts formed post-weaning as representative of the adult diet since hunter gatherers are weaned on average around 4 years[81].

3.3.2 Faunal Teeth Sampling Approach

3.3.2.1 Estimation of the period of teeth formation

Determining the age of mineralization in fauna can be challenging due to limited data availability. However, we based our estimations on existing information for specific species (Supplementary table 17, Excel document):

Caprids (e.g., Barbary sheep): M2 mineralizes between 8 and 18 months, while M3 mineralizes between 19-36 months. Caprids are typically weaned around 4 months, which means that these two teeth (M2 and M3) are not influenced by nursing[85]. However, it's worth noting that the enamel of incisors can be impacted by nursing.

Equids: Equids are typically weaned between 9-15 months[86]. The P2, P3, P4, and M3 mineralize after weaning, while M2 partially mineralizes before weaning. The deciduous premolars (dp) of Equids are unlikely to be affected by nursing, as they begin calcifying before birth and are fully calcified shortly after birth.

Bovids: Bovids are generally weaned between 6-8 months. The M1 mineralizes before weaning, whereas the M2 and M3 mineralize after weaning. The premolars also mineralize after weaning[87].

Carnivores: Carnivores are usually weaned at an earlier age. For example, foxes are weaned around 41 days, but in the case of the enamel on M1, it might be affected by nursing signals[88]. However, the root of M1 is formed post-weaning. We could not determine the exact species of the other canid but the pattern of weaning is likely to be similar.

3.3.2.2 Fauna teeth sampling

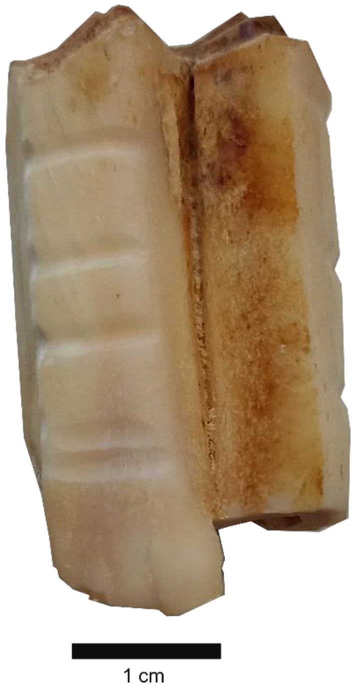

Supplementary Figure 2: An example of sampling strategy for Zn isotope analyses on a herbivore tooth

4 Supplementary Information 4. Results

4.1 Zinc isotopic results

4.1.1 Data quality

The zinc analyses incorporated two reference materials, SRM 1400 and SRM 1486, which were prepared and subsequently analyzed alongside the samples. The obtained values fell within the established range of previous findings documented in the literature (Supplementary Table 5).

Zinc was extracted from the tooth enamel of human and faunal remains which is generally more resistant to diagenetic alteration[47,89]. Zinc isotope ratios correlation between concentrations in tooth enamel is used to test the good preservation of dietary biogenic δ^{66}Zn values[90]. There is a slight correlation between the Zn isotope ratios and the concentrations (1/conc) in our samples (p-value < 0.001) (Supplementary Figure 3), however, this correlation is likely of dietary origin: the human enamel has systematically higher Zn concentration than that of the fauna and, since most of our samples consist of herbivores which usually have lower Zn concentrations, a correlation between δ^{66}Zn and concentrations appears (Supplementary Figure 3). There is no correlation between the Zn concentration and δ^{66}Zn values if we consider human and fauna elements separately (Supplementary Figure 3), which argues for the good preservation of the biogenic zinc isotope ratios.

Supplementary Table 5: Zinc isotope ratios of reference materials from this study and literature data.

	**This study**			**Literature data**		
	**δ^{66}Zn**	**SD**	**n**	**δ^{66}Zn**	**SD**	**References**
SRM 1400	0.93	0.03	5	0.96	0.02	[39] [38,43,45,48,52]
SRM 1486	1.23	0.04	2	1.2	0.04	[52,53]

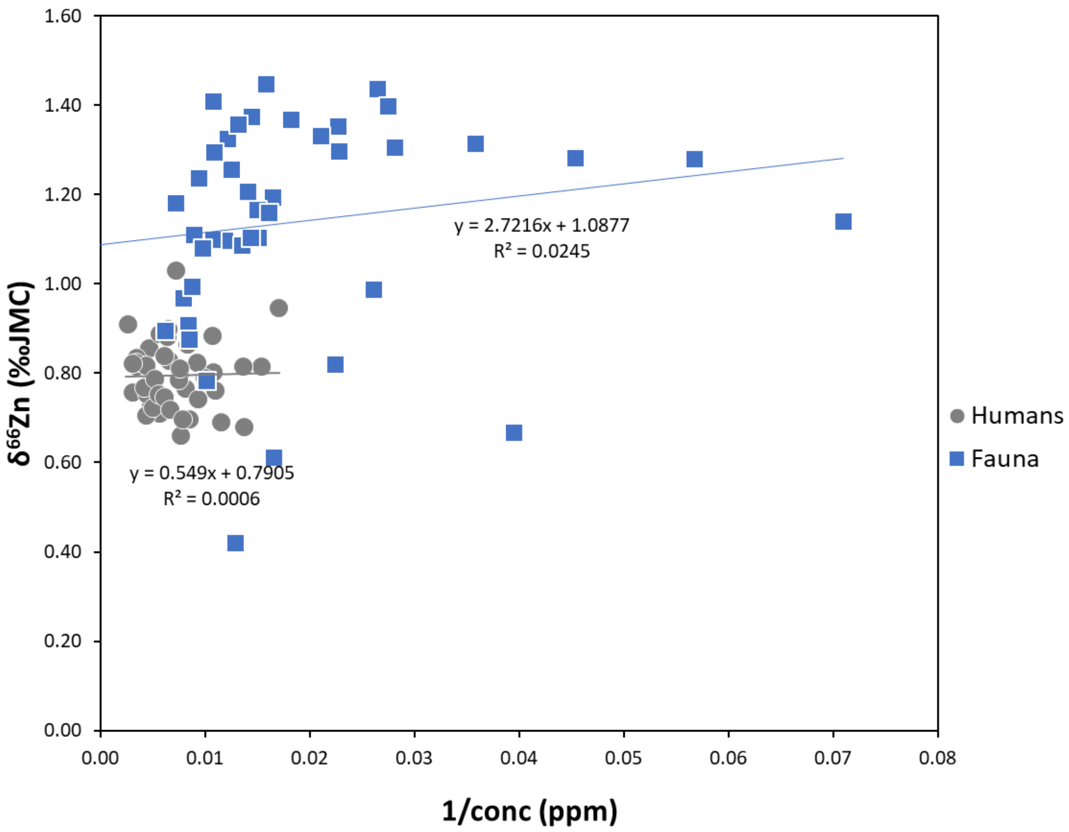

Supplementary Figure 3: Relationship between the Zn isotope ratios and Zn concentrations in enamel of fossil teeth from Taforalt, Morocco.

4.1.2 Description of the Zn isotope ratios of Taforalt fauna and animal teeth

The $\delta^{66}Zn_{enamel}$ values of the fauna from Taforalt ranges from 0.42 to 1.45‰ which is comparable with the range observed in the modern food web of Koobi Fora (Kenya) and Gabasa (Spain)[39,46]. When considering only the teeth formed post-weaning in herbivores, they exhibit the highest $\delta^{66}Zn_{enamel}$ values in the food web at Taforalt (1.12±0.07‰, $n_{samples}$=20). The carnivores (a fox and a canid), on average, show lower values (0.52 ± 0.14‰, n=2) (Figure 3). While the $\delta^{66}Zn_{enamel}$ value of the M1 enamel sampled from the fox may be influenced by the nursing signal (0.61‰), the canid's molar exhibits a lower $\delta^{66}Zn$ value (0.42‰) and is less likely to be affected by nursing[48]. While our preliminary data suggests differences in $\delta^{66}Zn_{enamel}$ values between dietary groups (herbivores and canids) (Extended Data Figure. 1) , it is important to note that our sample size is limited for the carnivores and that they might not have been purely carnivorous and occasionally

included plants into their diet[73]. However, $\delta^{66}Zn_{enamel}$ values for the carnivores at Taforalt are still lower than values observed in other food webs, even lower than wolf[39] which is usually considered a pure carnivore[73]. The trophic level spacing $\Delta^{66}Zn_{herbivores\text{-}canids}$ would be 0.60‰ and can be 0.70‰ if we exclude the M1 of the fox. Although based only on two carnivores, the trophic spacing at Taforalt fits with ranges observed in other food webs[38,46,52]. The humans from Taforalt have elevated $\delta^{66}Zn_{enamel}$ values (0.78 ± 0.07‰, $n_{samples}$= 28) that are close to that of the herbivores from the same site. The offset between the adult humans and the herbivores is 0.34‰.

4.1.2.1 Zinc variability among herbivores

In Taforalt, we observe a pattern of $\delta^{66}Zn_{enamel}$ between grazer and mixed feeder herbivores (Kruskal-Wallis chi-squared = 19.737, df = 1, p-value < 0.05) (Extended Data Figure.1). Grazers have the highest $\delta^{66}Zn_{enamel}$ values (1.28‰ ± 0.15, n= 21). The mixed feeders (i.e. Barbary sheep and gazelle) have lower $\delta^{66}Zn$ values (Extended Data Figure.1). This pattern is similar to the one observed in the modern food web of Koobi Fora in the African Savannah[46], and could be due to their consumption of leaves and fruits. The roots, shoots and all low growing plants are usually more enriched in heavy Zn isotopes, while tree leaves have lower $\delta^{66}Zn_{enamel}$ values[59,91].

4.1.2.2 Zinc isotopic variation among human individuals

The zinc isotope values do not vary significantly among the individuals analysed if we consider the average value of each individual (Kruskal-Wallis chi-squared = 5, df = 5, p-value = 0.4159). No discernible sex-related distinction was observed in the Taforalt population. The zinc isotope values, as illustrated in (Supplementary Figure 4), revealed no significant relationship with the sex of adult individuals (Kruskal-Wallis chi-squared = 0.49522, df = 1, p-value = 0.4816, Supplementary Table 6). This observation holds true for infants as well. It is important to note that the analysis of adult values exclusively includes teeth formed post-weaning, while those formed pre-weaning are categorized as part of the infant diet.

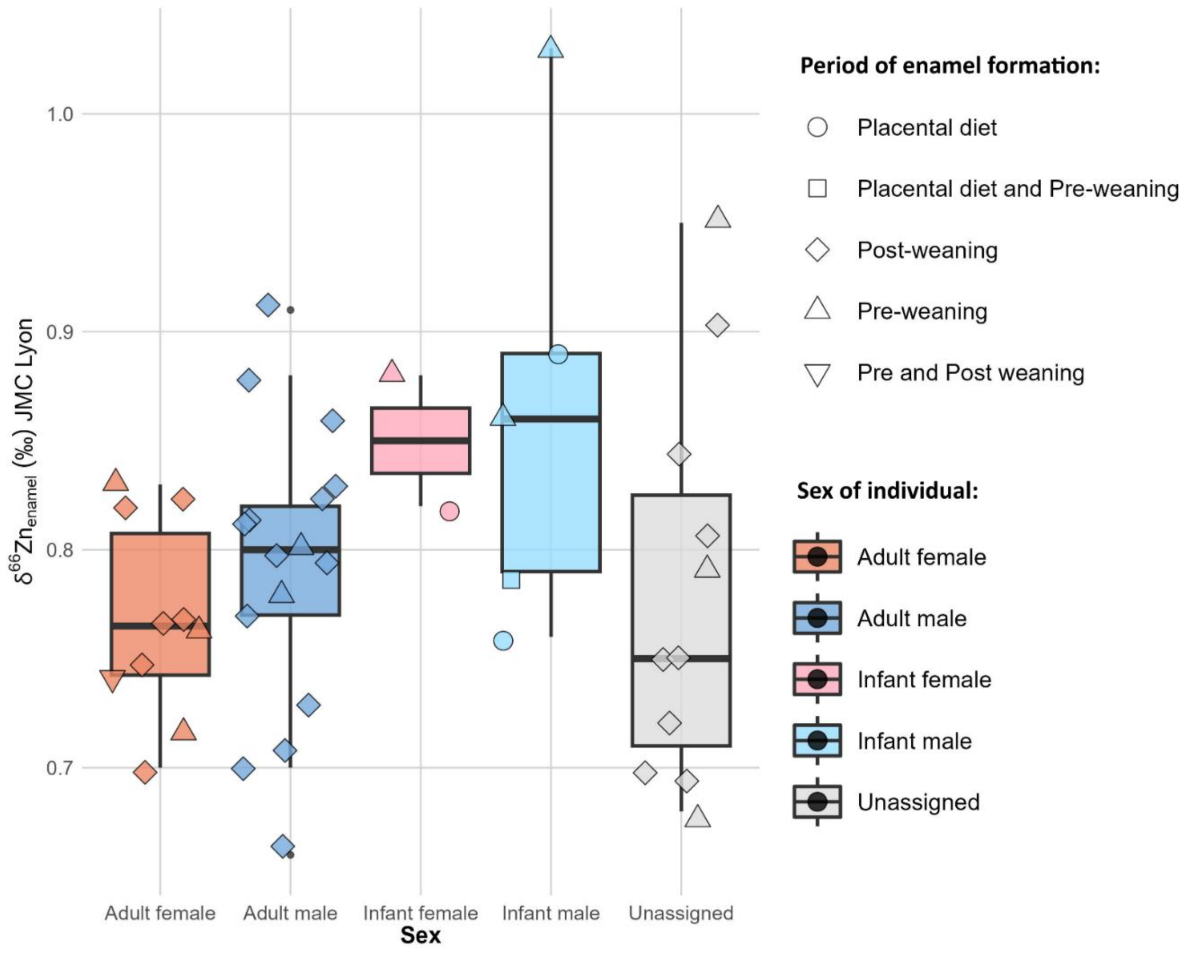

Supplementary Figure 4: Boxplot of zinc isotope values obtained from teeth enamel from male and female samples in Taforalt ($n_{individual}$=17, 45 samples in total). Each point represents a single sampling from a tooth. Boxes correspond to the median (centre line) and the first and third quartiles, while whiskers indicate the minimum and maximum values.

Supplementary Table 6: Chi-squared and p-value of Kruskal Wallis tests comparing Zn_{enamel} isotope ratios with the sex of the human samples from Taforalt.

Age group	χ2	p value	p-value<0.05
Biological sex (Adult)	0.49522	0.2652	No
Biological sex (Infant)	0.42346	0.5152	No

For some of the individuals, several teeth were sampled. In general, the variability between teeth is quite small, within 0.1‰ of standard deviation. There are not enough teeth to conduct statistical

analyses. Individual 1 has mostly teeth formed after the introduction of solid food in the diet of a child. Three M2 have been analysed, along with P4, C and I2, and the M2 exhibit the lowest and highest ratios (Extended Data Figure.3). We do not see any weaning pattern for this individual. Individual 5 show higher ratios in their I1 and M1 compared to M2 and C, but the values are very similar. Individual 6 had only deciduous teeth, and the dm2 shows the highest ratios, which is consistent with the hypothesis than exclusive breastfeeding triggers higher δ^{66}Zn values than a placental diet (recorded by di1).

4.2 Strontium isotopic results

4.2.1 Data quality

For strontium isotope ratios, our analysis involved the preparation and examination of the standard SRM 1986 alongside the samples. The $^{87}Sr/^{86}Sr_{enamel}$ values obtained from this standard fall consistently within the established range of published values (Supplementary Table 7). The $^{87}Sr/^{86}Sr_{enamel}$ and Sr concentration of our samples show no correlation, which suggests the preservation of biogenic strontium isotope ratios (Supplementary Figure 5).

Supplementary Table 7: Strontium isotope ratios of reference materials from this study and literature data [38,39,43,45,46,48,92].

	**This study**			**Literature data**		
	**$^{87}Sr/^{86}Sr$**	**SD**	**n**	**$^{87}Sr/^{86}Sr$**	**SE**	**References**
SRM 1486	0.7093	2E-05	7	0.7093	3E-05	Long term value at MPI-EVA, n=137

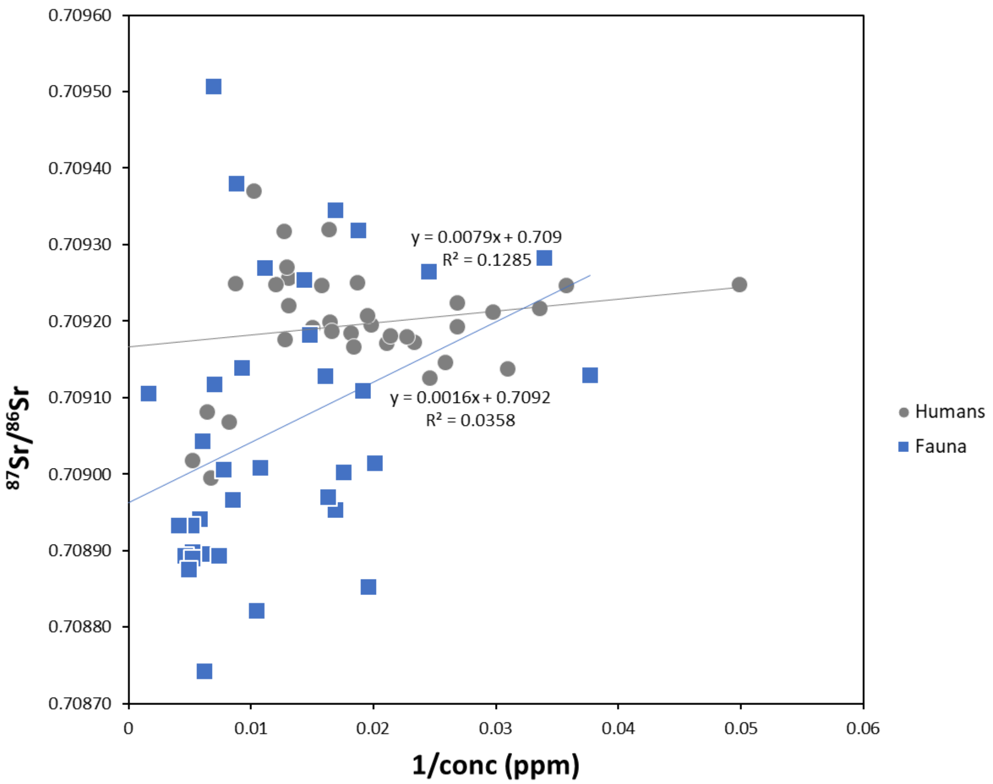

Supplementary Figure 5: Relationship between the Sr isotope ratio and Sr concentration in enamel of human and fauna teeth from Taforalt, Morocco.

4.2.2 <u>Strontium isotope data</u>

The $^{87}Sr/^{86}Sr_{enamel}$ of fauna and humans display a narrow range, from 0.7089 to 0.7093, and 0.7090 to 0.7094 respectively, compatible with what is expected for a coastal region (<100km) dominated by a calcareous bedrock from Late Jurassic[2,61,65,93]. Among all the enamel samples analyzed in this study, no correlation between the Zn isotope and Sr isotope ratio has been detected (Figure 2, Main paper). This suggests that the Sr isotope composition in local geology is relatively homogenous and has no influence on the Zn isotope ratios at Taforalt. All humans show $^{87}Sr/^{86}Sr$ compatible with that of the fauna, which is compatible with the hypothesis that they were all local individuals.

4.3 Carbon and nitrogen results

4.3.1 Data quality

Carbon and nitrogen stable isotopes were measured from bulk collagen. The weight percentage of carbon (wt% C) and nitrogen (wt% N) in our samples range from 47.8 to 14.4% and 17.1 to 5.6% respectively, and the C/N ratios fall within the acceptable range for ancient collagen extracts (3.0-3.3)[23,94] (Supplementary table 16, Supplementary table 17 , Excel document). We thus exclude the possibility of diagenetic alteration affecting our collagen isotope values[94]. The $\delta^{13}C$ and $\delta^{15}N$ of the reference material that was analysed along with our samples fall within the range of accepted values (Supplementary Table 8).

Supplementary Table 8: Carbon and nitrogen isotope ratios of reference materials from this study and the accepted values (https://nucleus.iaea.org/sites/ReferenceMaterials/Pages/Stable-Isotopes.aspx)

	This study					Expected values	
	**Material**	**$\delta^{13}C$ (‰, VPDB)**	**SD**	**$\delta^{15}N$ (‰, AIR)**	**SD**	**$\delta^{13}C$ (‰, VPDB)**	**$\delta^{15}N$ (‰, AIR)**
**IAEA N1 CH6 (n=12)**	ammonium sulfate	-10.47	0.3	0.43	0.04	−10.449 ± 0.03	0.43 ± 0.07
**IAEA N2 CH7 (n=12)**	ammonium sulfate	-32.13	0.04	20.35	0.05	−32.151 ± 0.05	20.41 ± 0.12

4.3.2 Carbon and nitrogen isotope data

The $\delta^{15}N_{collagen}$ values of the herbivores display a wide range of variation (5.5 to 11.7‰) and the $\delta^{13}C_{collagen}$ values ranges from -20.91 to -18.06‰ (Figure 2, Main paper). Various factors could influence this variation. Notably, nursing can raise herbivores $\delta^{15}N_{collagen}$ levels, which is the case for a deciduous tooth of a Barbary sheep ($\delta^{15}N_{collagen}$ = 11.7‰) and a deciduous tooth of an equid ($\delta^{15}N_{collagen}$ = 8.7‰). After excluding potentially nursing-affected teeth, the values still span between (5.5 to 11.6‰). One possible cause is the metabolic adaptation of certain species to drought conditions, exemplified by the elevated $\delta^{15}N_{collagen}$ values in the hartebeest species, known for their ability to survive extended periods without water[77]. Additionally, variations in $\delta^{15}N$ baseline values may play a role, as seen in Barbary sheep, which possess a flexible diet and may consume plants with differing $\delta^{15}N$ values; For the $\delta^{15}N_{collagen}$, the trophic level spacing between

carnivores and herbivores, considering only post-weaning tissues, is only of 2.8‰ which is in lower than the common 3-5‰ trophic level increase[29,95–97].

The $\delta^{13}C_{collagen}$ and $\delta^{15}N_{collagen}$ for adult humans (tissues formed post-weaning) are between -19.3 to -18.4‰ and 8.8 to 12.1‰ respectively. The $\delta^{13}C_{collagen}$ and $\delta^{15}N_{collagen}$ of the infant humans (tissues formed pre-weaning) are between -18.8 to -18.6‰ and 12.3 to 13.5‰ respectively. Contrary to Zn, there is an overlap between some herbivores, humans and carnivores (Figure 2 main paper). If we included only adult $\delta^{15}N_{collagen}$ values, the overall trophic offset between adult humans and herbivores is +2.5‰ and it is slightly larger with the Barbary sheep, the most dominant species in the site (+4.1‰). There were not enough carnivores (n=2) to accurately estimate the trophic level effect TLE but based on our data it is around +2.8‰ for $\delta^{15}N_{herbivore\text{-}canid}$. Although the canids could have been omnivorous, our CSIA analysis on nitrogen show that one of the specimens has a carnivore trophic level (TP=2.9) (See section 4.4). The analyses were performed on the root of the M1 of these individuals, less likely to be influenced by the nursing effect than the crown (see section 3.3.2.2). The $\delta^{15}N_{collagen}$ values show no significant relationship to the sex of the human individuals (Supplementary Table 9).

Supplementary Table 9: Chi-squared and p-value of Kruskal Wallis tests comparing N isotope ratios with the sex of the human samples from Taforalt

No age considered	chi-squared	p value	p-value<0.05
Biological sex (Adult)	0.3247	0.5688	No
Biological sex (Infant)	0.22059	0.6386	No

4.3.3 Comparison with published results from Taforalt (Lee-Thorp et al., 2019)[98]

In our study, we observed a trophic level spacing ($\Delta^{15}N_{collagen}$ $TLS_{herbivore\text{-}human}$) of 2.5, which differs from the $\Delta^{15}N_{collagen}$ TLS value of +4.2‰ reported by Lee-Thorp et al. for Taforalt[59,91]. This variation in $\Delta^{15}N_{collagen}$ TLS values suggests potential differences in the species sampled for the isotopic analyses. Notably, Barbary sheep was the most frequently sampled species in Lee-Thorp's study. Lee-Thorp et al.[98] reported an average $\delta^{15}N_{collagen}$ value of +6.42 ± 1.72‰ for all the

herbivores, while our study observed an average $\delta^{15}N_{collagen}$ value of 8.3 ± 3.16‰. Our study and Lee-Thorp et al. both found substantial variability in $\delta^{15}N$ values for Barbary sheep. This variability in $\delta^{15}N$ values within the Barbary sheep population may be attributed to the flexible dietary habits of this species, and it underscores the challenges of characterizing a stable baseline when considering this species (Supplementary Table 10). No carnivore (or canid) was analysed in their study to estimate the trophic level effect. Furthermore, it is essential to note that our study employed both teeth and bone collagen for isotopic analysis, whereas Lee-Thorp et al.[98] focused solely on bone collagen.

Apart from Barbary sheep, we observed similar $\delta^{15}N_{collagen}$ values for other species (Supplementary Table 10). Comparing our results to Lee-Thorp et al.[98], the $\delta^{15}N_{collagen}$ values for Equids and *Gazelle* sp. in both datasets show alignment, indicating some consistency in the dietary patterns of these species in the Taforalt region. Furthermore, both studies observed no evidence of marine resource consumption by the population, a finding that corroborates the conclusion that the Taforalt population had a limited marine dietary component.

In their study, Lee-Thorp et al.[98] also analyzed the bone collagen of some of the same human individuals as in our study (Supplementary Table 10). The $\delta^{15}N_{collagen}$ values obtained for bone collagen in their analysis closely align with our results. However, it is worth noting that there was one exception, where individual 13 exhibited a relatively higher $\delta^{15}N_{collagen}$ value (12.1‰) compared to the values observed by Lee-Thorp et al.[98] (10.4‰). One potential explanation for this variation could be that we sampled different bones with varying turnover rates. Bones with different turnover rates, such as rib bones and long bones, can record different aspects of an individual's diet over time[99]. However, the specific bone type analyzed for isotopic differences was not specified in the previous publication.

Supplementary Table 10: Comparison between the average $\delta^{15}N_{collagen}$ values obtained from Lee-Thorp et al.[98] and the values obtained in our study from the same species and human individuals

Study	Barbary sheep	Equids	Gazella	Ind.5	Ind.6	Ind.9	Ind.13	Ind.14
Lee-Thorp (bone)	6.4‰	8.1‰	7.4‰	9.7‰	12‰	12.1‰	10.4‰	10.9‰
This study (all tissues	8.3‰	8.3‰	7.8‰	10.3‰	12.8 ‰	12.6‰	11.8‰	-
This study (bone)	-	-	-	-	12.5 ‰	12.3‰	12.1‰	-

4.4 Carbon and nitrogen on single amino acids results

4.4.1 Data quality

We successfully obtained the amount of collagen required for conducting Compound-Specific Isotope Analysis (CSIA) for 54% of the samples.

Carbon and nitrogen isotope measurements on single amino acids were commercially performed by the University of Davis. The measurements are calibrated against certified standard reference materials from USGS and IAEA. Internal reference materials with known isotope values are used to measure accuracy and reproducibility. Reference materials were analyzed along with the samples and gave results consistent with expected values (Supplementary Table 11). For Carbon and Nitrogen, the His, Met are below LOQ for all samples. Hyp, Ile, Tyr are below LOQ for select samples.

In addition, we performed two additional controls:

1) We sent the reference material SRM 1477c (bovine liver) along with our samples to the University of Davis for the analyses. The SRM 1477c was previously analysed and published in Jaouen et al (2019)[100] (Supplementary Table 11). The carbon, the amino acids Ala, Gly, Leu, Lys, Val, Pro Phe and Val are within the values published in Jaouen et al. 2019[100] except for the Asx, Glx and Thr. Since the publication of Jaouen et al., 2019, significant advancements have been made in measuring carbon isotope values of threonine (Thr). Unfortunately, there is currently a lack of available carbon isotopic data on Thr for SMR1577C beyond the aforementioned study. We should also mention that we did not include the Thr results in our PCA. For nitrogen (Supplementary Table 11) Ala, Asx, Glx, Leu, Pro, Ser, Thr and Val are within the published values except for Lys, Phe. For some of the amino acids, our current findings differ from those reported in Jaouen et al. 2019, but we cannot definitively determine whether our values or the values published in 2019 are accurate.
2) Following the recommendations of O'Connell and Collins (2017)[101], we verified the existence of a correlation between isotope ratios measured in hydroxyproline and proline (Supplementary Figure 6; Supplementary Figure 7).

According to a previous study, the correlation between the $\delta^{15}N$ values of the proline (Pro) and hydroxyproline (Hyp) is essential to assess the data quality (O'Connell and Collins, 2018)[101]. Since the nitrogen in Hyp is synthesized from Pro[101], their $\delta^{15}N$ values are expected to be relatively equal and the plot of $\delta^{15}H_{Hyp}$ vs. $\delta^{15}N_{Pro}$ should give a slope close to 1. We verified this on our samples and it shows that there is a strong correlation between the $\delta^{15}N$ values of these amino acids (y= 0.9729x+ 0.1639; R^2 = 0.9898) (Supplementary Figure 6).

For $\delta^{13}C$, the $\delta^{13}C_{Hyp}$ was plotted against $\delta^{13}C_{Pro}$ but the slope was (y = 0.8652x - 3.276 R^2 = 0.5741), which is inconsistent with the slope of one expected. Hydroxyproline data are systematically enriched compared to proline data. For this reason, we plotted in grey color (Supplementary Figure 7) the samples that showed an offset superior to 1‰ between $\delta^{13}C_{Hyp}$ and $\delta^{13}C_{Pro}$. It corresponds to nine samples out of 29 samples.

Supplementary Table 11: Carbon and nitrogen isotope ratios of single amino acids measured in the reference material SRM 1577c in our study compared to published values in Jaouen et al., 2019[100]

	Ala		Asx		Glx		Gly		Leu		Lys	
	This study	***Jaouen et al. 2019***	***This study***	***Jaouen et al. 2019***	***This study***	***Jaouen et al. 2019***	***This study***	***Jaouen et al. 2019***	***This study***	***Jaouen et al. 2019***	***This study***	***Jaouen et al. 2019***
**Mean δ^{13}C value**	-20.18	-19.82	-16.06	-12.81	-14.63	-16.56	-10.04	-8.10	-28.89	-29.66	-16.64	-15.89
**SD**	1.14	0.47	0.91	0.25	0.51	0.41	0.98	1.15	0.01	0.71	0.17	1.2
**n**	2	3	2	3	2	3	2	3	2	3	2	1
	**Phe**		**Pro**		**Ser**		**Thr**		**Tyr**		**Val**	
	This study	***Jaouen et al. 2019***	***This study***	***Jaouen et al. 2019***	***This study***	***Jaouen et al. 2019***	***This study***	***Jaouen et al. 2019***	***This study***	***Jaouen et al. 2019***	***This study***	***Jaouen et al. 2019***
**Mean δ^{13}C value**	-25.15	-25.1	-18.35	-18.68	-7.78		-9.74	-7.23	-30.9		-24.57	-25.2
**SD**	0.23	0.50	0.59	0.19	0.77		0.06	0.25	1.2		0.18	0.3
**n**	2	3	2	3	2		2	1	2		2	3

	**Ala**		**Asx**		**Glx**		**Leu**		**Lys**		**Phe**	
**Mean**	***This study***	***Jaouen et al. 2019***	***This study***	***Jaouen et al. 2019***	***This study***	***Jaouen et al. 2019***	***This study***	***Jaouen et al. 2019***	***This study***	***Jaouen et al. 2019***	***This study***	***Jaouen et al. 2019***
**$\delta^{15}N$**	12.18	10.37	10.01	10.27	10.69	10.30	7.24	4.95	7.02	2.75	10.10	8.25
**SD**	0.38	0.61	0.88	1.15	0.68	0.40	0.16	1.86	0.67	1.11	0.38	0.12
**n**	2	3	2	3	2	3	2	3	2	2	2	3
	**Pro**		**Ser**		**Thr**		**Tyr**		**Val**			
**Mean**	***This study***	***Jaouen et al. 2019***	***This study***	***Jaouen et al. 2019***	***This study***	***Jaouen et al. 2019***	***This study***	***Jaouen et al. 2019***	***This study***	***Jaouen et al. 2019***		
**$\delta^{15}N$**	14.91	13.70	10.85	9.37	-6.74	-5.77	4.76		10.48	10.77		
**SD**	0.02	0.57	0.78		1.2	0.88	0.81		0.73	0.63		
**n**	2	3	2	1	2	3	2		2	3		

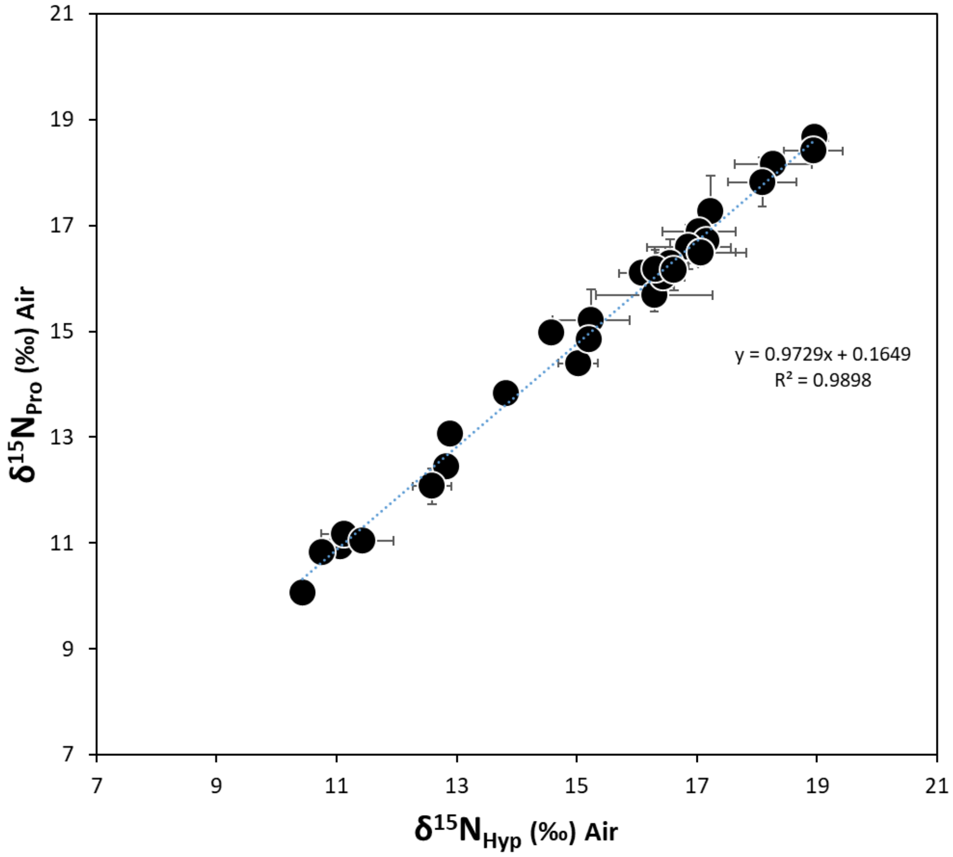

Supplementary Figure 6: Comparison of collagen proline and hydroxyproline δ^{15}N values of the fauna and humans from Taforalt ($n_{samples}$= 29). The error bars represent the standard deviation of measurements and the points represent the mean value of the measurements (centre)

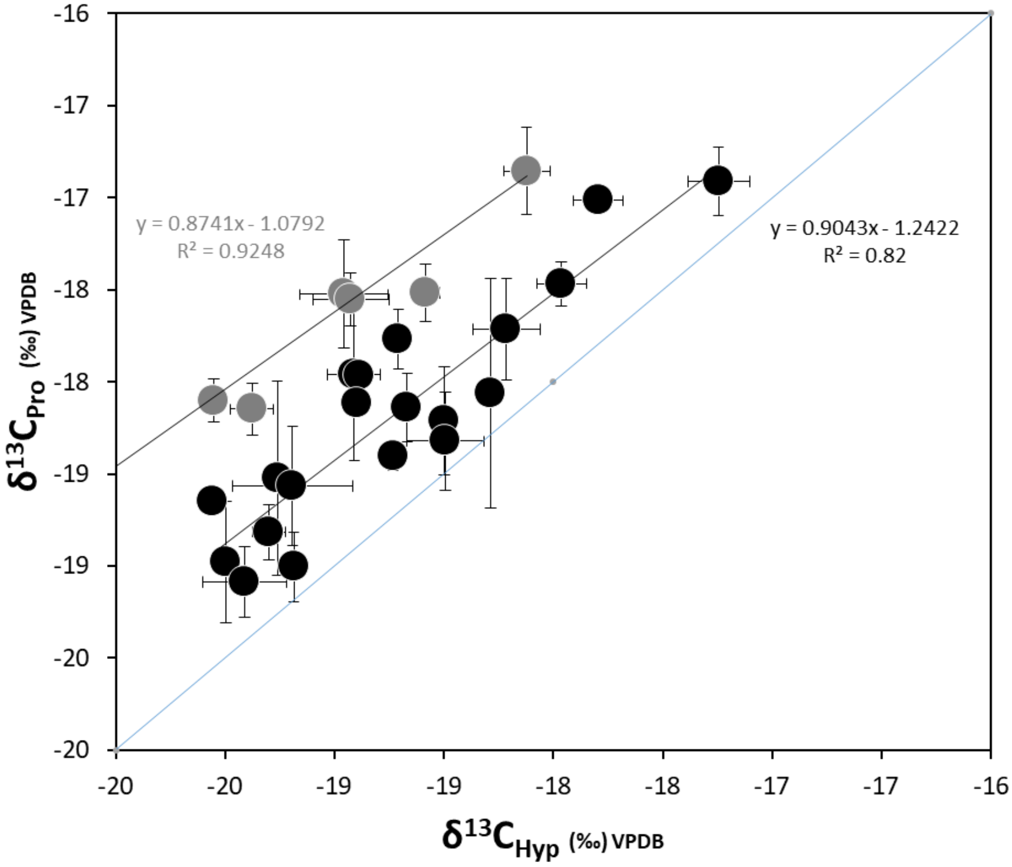

Supplementary Figure 7: Comparison of collagen proline and hydroxyproline $\delta^{13}C_{values}$ from our study ($n_{samples}$= 29). The blue line shows the expected slope x=y for the $\delta^{13}C_{Hyp}$ against $\delta^{13}C_{Pro}$. The black dots are the samples which showed an offset inferior to 1‰ between $\delta^{13}C_{Hyp}$ and $\delta^{13}C_{Pr}$; the grey dot showed an offset superior to 1‰ between $\delta^{13}C_{Hyp}$ and $\delta^{13}C_{Pro.}$ The error bars represent the standard deviation of measurements.

4.4.2 Description of carbon isotope ratios of amino acids in Taforalt samples

In order to assess the diet of the human from Taforalt using the individual amino acids isotopic values we used the principal component analysis (PCA) method used by Ma et al., 2021[102] to separate between different dietary groups (Extended Data Figure .2). We present the first two components with the calculated eigenvalues for each of the features used in the (Supplementary Table 23). The $\delta^{13}C_{collagen}$, $\delta^{13}C_{Pro,}$ $\delta^{13}C_{Asp,}$ $\delta^{13}C_{Lys,}$ $\delta^{13}C_{Ala,}$ $\delta^{13}C_{Val,}$ $\delta^{13}C_{Glx,}$ $\delta^{13}C_{Gly}$ have the highest positive effect on the first principal component (PC1), while $\Delta^{13}C_{Gly\text{-}Phe}$, $\Delta^{13}C_{Val\text{-}Phe}$, and $\delta^{15}N_{collagen}$

have most significant negative effect on the second principal component (PC2) (Supplementary Table 23).

When plotting the human and fauna from Taforalt with different dietary groups: C_3 animals, C_3 humans, C_4 humans, and the marine and freshwater consumers from published studies Extended Data Figure.2, Supplementary Figure 8, Supplementary Figure 9, Supplementary Figure 10) we see that our individual plot well with the C_3 consumers. This confirms their terrestrial C3 diet and the absence/ rare consumption of marine and freshwater resources. Some of the herbivores could integrate some C4 plants components into their diets, as they slightly differ from the range observed among C3 plant consumers in other studies (Extended Data Figure.2).

When looking at the bivariate plots of $\delta^{13}C_{Lys}$ vs. $\Delta^{13}C_{Val\text{-}Phe}$ as well as $\Delta^{13}C_{Gly\text{-}Phe}$ as suggested by Ma et al., 2021[102], the lysine carbon isotope values are consistent with a diet mostly relying on C3 resources, and the $\Delta^{13}C_{Val\text{-}Phe}$ and $\Delta^{13}C_{Gly\text{-}Phe}$ confirm the reliance on terrestrial resources (Supplementary Figure 8, Supplementary Figure 9). A PCA including CSIA Nitrogen isotope data is also available on Supplementary Figure 16.

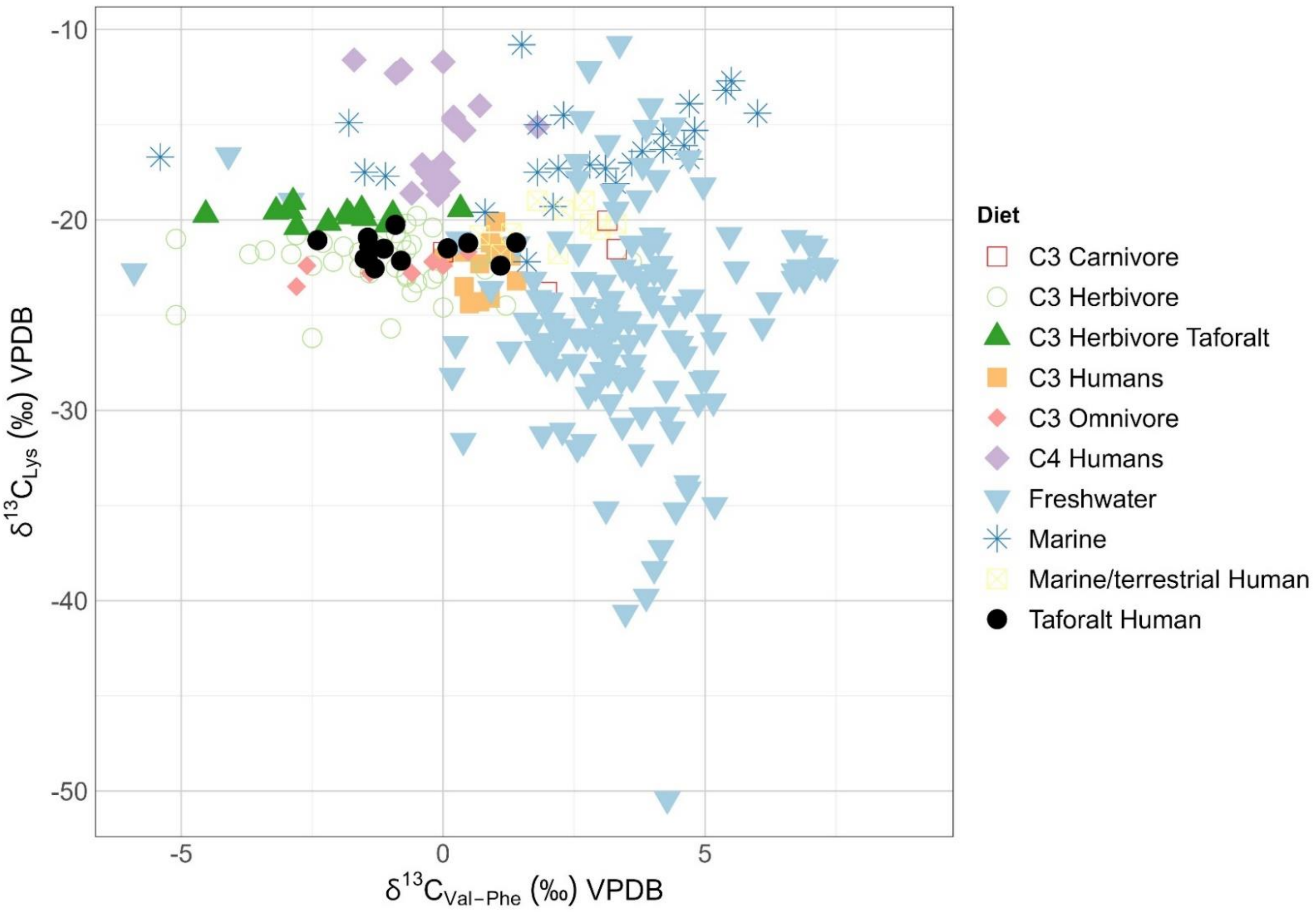

Supplementary Figure 8: Bivariate plots of the $\delta^{13}C_{Lys}$ vs. $\Delta^{13}C_{Val\text{-}Phe}$ of the samples from Taforalt and published data[35,72,100,103–106,106].

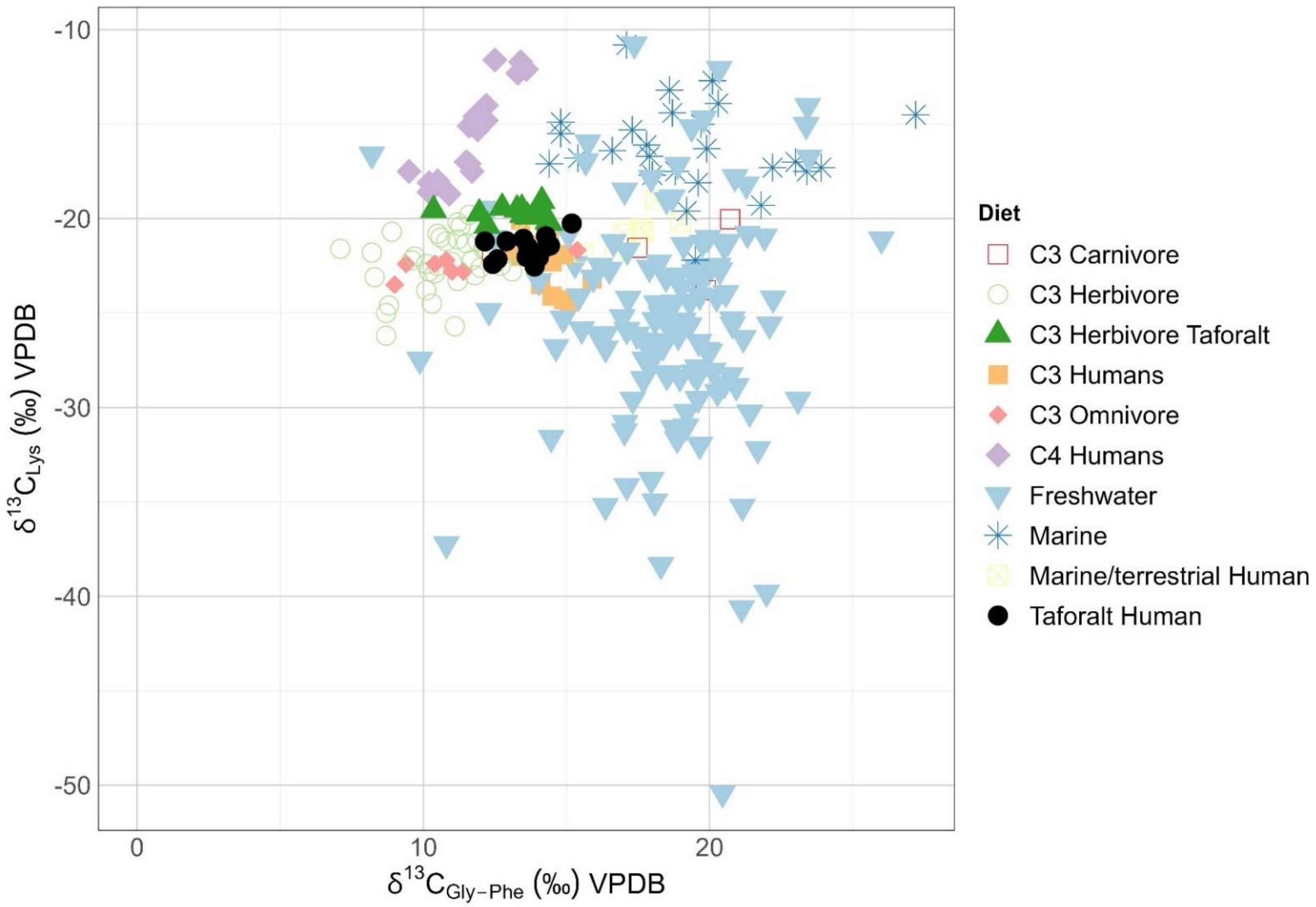

Supplementary Figure 9: Bivariate plots of the $\delta^{13}C_{Lys}$ vs. $\Delta^{13}C_{Gly\text{-}Phe}$ of the samples from Taforalt and published data[35,72,100,103–106]

Finally, we use the traditional representation of $\delta^{13}C_{Phe}$ towards $\delta^{13}C_{Val}$[35,36], which clearly indicate a terrestrial diet based on C3 plants and animals feeding on C3 plants (Supplementary Figure 10).

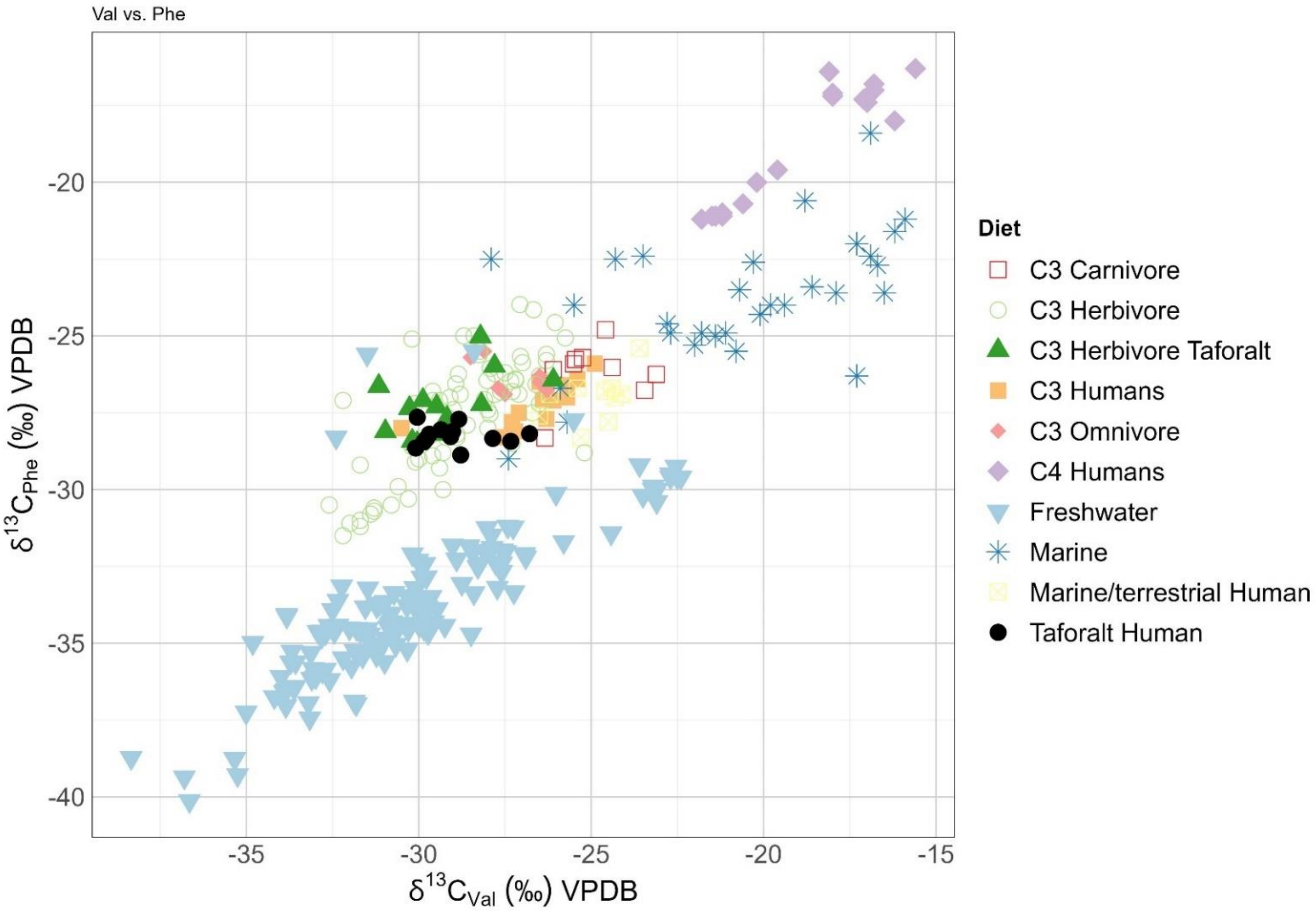

Supplementary Figure 10: Bivariate plot of the Taforalt $\delta^{13}C_{Phe}$ and $\delta^{13}C_{Val}$ results by dietary classification with known types of dietary consumers from published data[35,72,100,103–106]

4.4.3 Description of nitrogen isotope ratios of amino acids in Taforalt samples

4.4.3.1 Environmental variation of nitrogen isotope ratios

The $\delta^{15}N_{Phe}$ usually reflect the local baseline. In the overall samples, there is no correlation between the $\delta^{15}N_{bulk}$ and $\delta^{15}N_{Phe}$ (r=0.17, R^2= 0.04231, p-value: 0.1463) since this latter is not strongly affected by the trophic enrichment. The herbivores in Taforalt exhibit a wide range of $\delta^{15}N_{Phe}$ (9.1-16.7‰) implying nitrogen isotope ratio variations related to the plants that they consume, which in turn were impacted by environmental factors. A sample from Barbary sheep and an equid have the highest $\delta^{15}N_{Phe}$ values while the hare exhibits the lowest value (Supplementary Figure 11). There are also variations within species. The two Barbary sheep have distinctive $\delta^{15}N_{Phe}$ values of

16.7‰ and 11.6‰. However, they have similar TP (2.1). This indicates that the individuals had access to plants with different N isotopic signatures, which explains the difference in the bulk collagen. The humans $\delta^{15}N_{Phe}$ values fall within the range of herbivores (10.4-13.7‰, mean= 12.23‰, Supplementary Figure 11). The low $\delta^{15}N_{Phe}$ of the carnivores (n= 2; mean: 10.8±0.9‰) compared to the other species indicates that they prey on games with lower $\delta^{15}N_{Phe}$ values. The latter also have a low bulk collagen nitrogen value, explaining why the canids overlapped with humans in the $\delta^{15}N_{collagen}$. There is a moderate to strong correlation between the TP and $\delta^{15}N_{Phe}$ for the human samples (r= -0.693558, p-value<0.05). The humans with a lower trophic position seem to have a relatively higher $\delta^{15}N_{Phe}$.

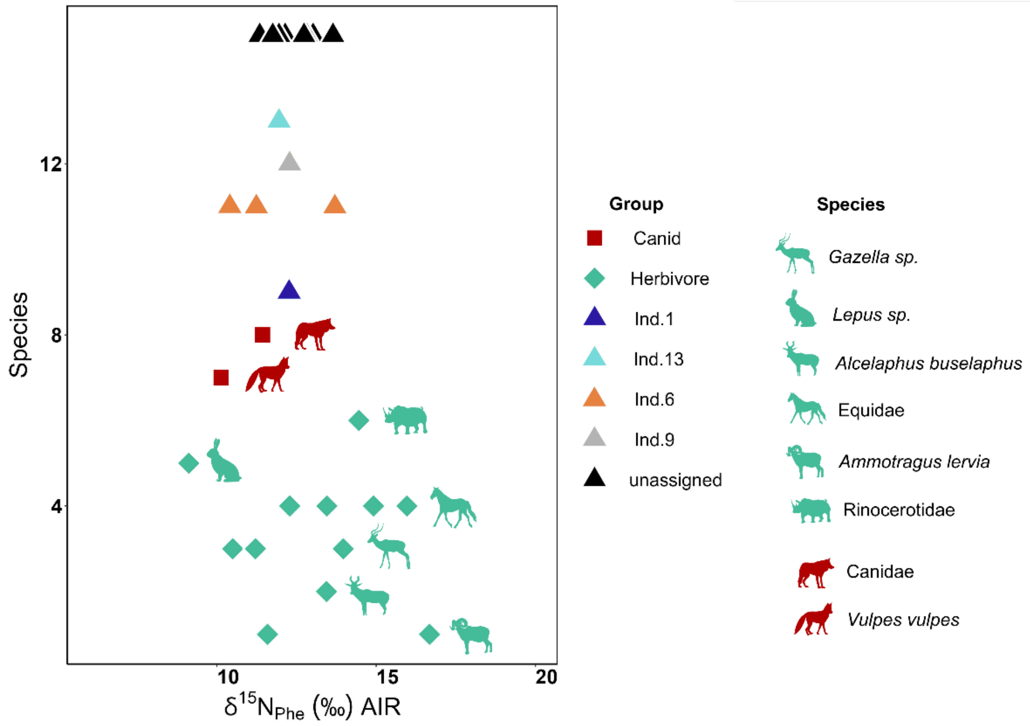

Supplementary Figure 11: The $\delta^{15}N_{Phe}$ values from the collagen of the different faunal species analysed from Taforalt in comparison with the human individuals

4.4.3.2 Trophic levels of the different fauna specimens

The $\delta^{15}N$ of amino acids generally shows the trophic position (TP) of an individual regardless the baseline variation effect. Thanks to our carbon isotope ratios, we know that there is no aquatic food consumption at Taforalt, and we can then use the C_3 equation[107] for the evaluation of the trophic positions of the fauna and humans. These equations show a range of 1.7 to 2.3 for adult

herbivores (average =2.1), which is close to the theoretical value of 2. This type of variation around the value of 2 has been previously observed[100,108,109].

For the Barbary sheep, both the tooth formed pre-weaning and post weaning have the same TP (TP=2.1) expected for herbivores, even the deciduous tooth. The TP of the deciduous teeth of the equid has also a TP of 2.1. As deciduous teeth are formed *in utero*, it is no surprise that the nursing effect cannot be observed.

Drought-tolerant herbivores show an average TP of 2.1 and the other herbivores of 2.0. This suggests that the metabolic adaptation to drought does not – or not strongly– influence the TP estimation from amino acids. We eliminate the possibility of suckling as an explanation for the high $\delta^{15}N_{collagen}$ values for the Barbary sheep and the Equid since their TP is 2.1 despite their high $\delta^{15}N_{collagen}$. Some herbivores have a TP above 2.1 it includes a hartebeest (TP= 2.3), hare (TP=2.3) and the rhinoceros (TP=2.3). It is possible that these individuals consumed plants with distinctive β factor[109]. It is unlikely that the TP of 2.3 represents a suckling effect especially for the hare with a $\delta^{15}N_{collagen}$ of 6‰.

The TP of the canid and the fox are respectively 2.9 and 2.7 (mean of 2.8) which is a little below the theoretical value of pure carnivores (TP=3) and could be explained by the opportunistic feeding habits of foxes. The second carnivore was only identified using traditional zooarchaeology as a canid. As the golden jackal (*Canis aureus*) is the most common Canidae in North Africa, we assume that our specimen might correspond to this species. The diet of the golden jackal consists primarily of small games (e.g., rodents, birds, hares...etc) and occasionally includes fruits[73,110].

4.4.3.3 Trophic levels among humans

The TP of human adults in the post-weaning period range from 2.6 to 2.2 (TP= 2.4±0.2, $n_{samples}$= 9), while the infants in the pre-weaning period have higher TP values 2.9-2.5 (TP= 2.7±0.2, $n_{samples}$=5).

The Ind.1 bone and an unassigned I2 showed a surprisingly low TP (2.2), also observed among herbivores due to the imprecision around the evaluation of the TP, indicating an almost exclusive vegetalian diet with a maximum of 20% of his dietary protein coming from meat. Knowing that

meat strongly dominates the intake of proteins in an omnivorous diet, the meat consumption is likely to be less than 20% in mass.

4.5 Sulphur isotope results

4.5.1 Data quality

We successfully obtained the required amount of collagen for sulphur isotope analyses for only three samples: a Barbary sheep, an equid and a human.

Reference materials were analyzed along with the samples and gave results consistent with the expected values (Supplementary Table 12). Sulphur isotope ratios were commercially analyzed by the society Isoanalytical are presented in the Supplementary Figure 12.

Supplementary Table 12: Reference standards used for sulphur stable isotopes analyses along with the accepted values

Standard	material	n	$\delta^{34}S_{V\text{-}CDT\ (mean)}$	SD	accepted values
IA-R061	(barium sulphate)	3	20.26	0.03	20.33
IAEA-SO-5	(barium sulphate)	2	0.35	0.19	0.50
IA-R068	(soy protein)	2	5.09	0.13	5.25
IA-R069	(tuna protein)	2	18.89	0.15	18.91

4.5.2 Sulphur isotopic data

Only three samples had enough collagen for the sulphur analyses. A Barbary sheep and an equid gave values of +14.6 and +12.5‰, respectively. The unassigned human sample has a δ^{34}S of +12.87‰, which falls within the range of the herbivores (Supplementary Figure 12). The relatively

high $\delta^{34}S$ in the samples from Taforalt possibly indicates the proximity of dietary resources for both humans and non-human fauna to the coast ("sea spray effect"), since terrestrial animals are expected to have values lower than +10‰[70]. Although the site is 40 km from the coast, the sea spray effect can extend many kilometers inland in some cases[68,70]and could have affected the vicinity around the Taforalt site [93]. Another explanation for the high $\delta^{34}S$ values in our sample is dust aerosols originating from the Sahara, which are rich in sulphur containing gypsum with high $\delta^{34}S$ values between +12 to +16‰[93,111,112]. The restricted number of samples analyzed here does not allow us to draw further conclusion.

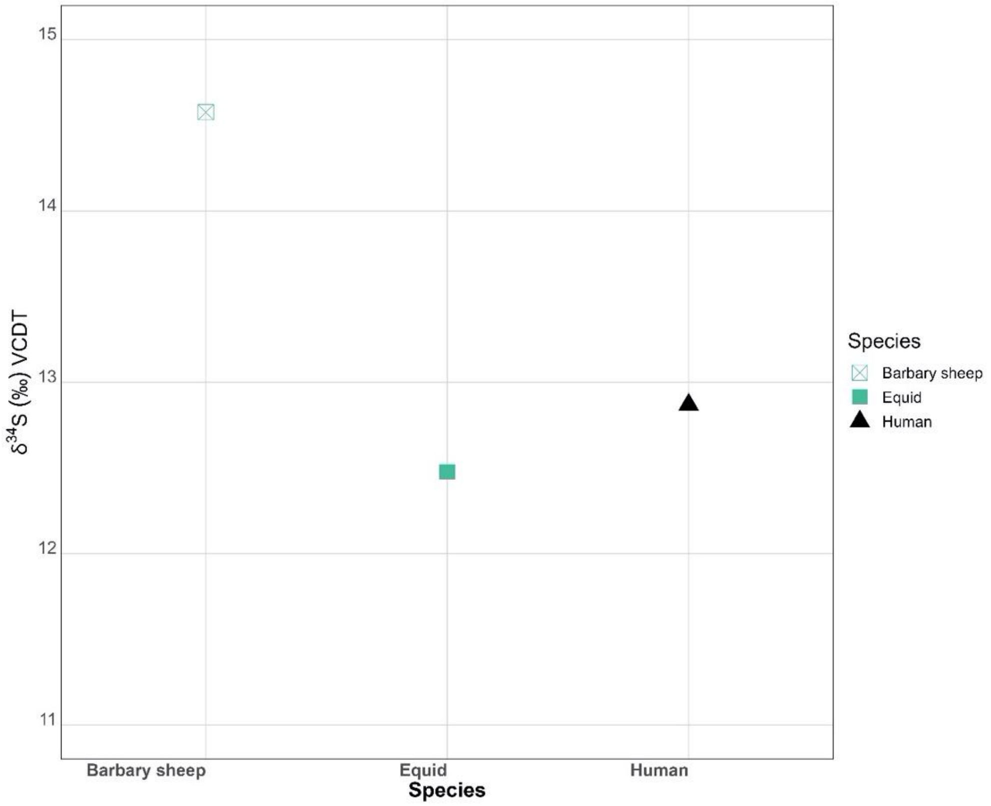

Supplementary Figure 12: Sulphur isotopic results of the fauna and an unassigned human from Taforalt. Barbary sheep: SEVA 34566, Equid SEVA 34565 Human (unassigned): SEVA 34561

4.6 Correlation between tracers

There is a strong negative correlation between $\delta^{66}Zn_{enamel}$ and trophic position (TP) (r =-0.73, R^2=0.51, p-value<0.05). However, if we exclude the infants the correlation of TP with $\delta^{66}Zn_{enamel}$ (r =-0.78, R^2=0.59, p-value<0.05) is even stronger than with $\delta^{15}N_{collagen}$ (r=0.69, R^2=0.45, p-value<0.05). The carnivores overlap with the humans and some herbivores in the N from bulk collagen while with $\delta^{66}Zn$ and $\delta^{15}N$ on amino acids, the canids clearly have a higher trophic level

than the humans. This indicates that Zn isotope ratios are less impacted by baseline effects than bulk nitrogen isotope ratios (Figure 2 main paper).

5 Supplementary information 5: Additional discussion

5.1 Weaning at Taforalt:

Although a tooth (m2) formed during the breastfeeding period exhibits relatively high Zn isotope ratios (0.79±0.04‰) (Extended Data Figure. 3), the zinc isotopic variation between enamel samples, including samples belonging to the same tooth, is not significant (n= 45, Kruskal-Wallis chi-squared = 3.6049, df = 2, p-value = 0.1649). A M1 has a quite small value (Supplementary Figure 13) in contradiction to what has been observed in Lapa do Santo (hunter gatherer from Brazil) and Rennes (medieval Breton population)[35,37] (**Error! Reference source not found.**) but matching the conclusions of two other studies performed on a single hominin individual showing that an M1 can record a post-weaning signal[32,75] (**Error! Reference source not found.**). First molars have their crown formed between birth and 2.5yrs, and our sampling selected parts formed between ≈1 to 2.5 years old (See Supplementary Information 7, Teeth scans). The variations seen in teeth for the individual 5 (Extended Data Figure. 3) could suggest that the canine and the M1 record the end of the weaning. One unassigned individual has a quite high values in their I2 compared to the rest of the population, which could suggest a longer breastfeeding period compared to other individuals. Zinc mostly deposits during the maturation phase of the enamel, and not at the earlier stages of the formation[40,113]. This may explain why we do not see a clear signature of breastfeeding at Taforalt, if weaning already started around 1 year of age for most of the population.

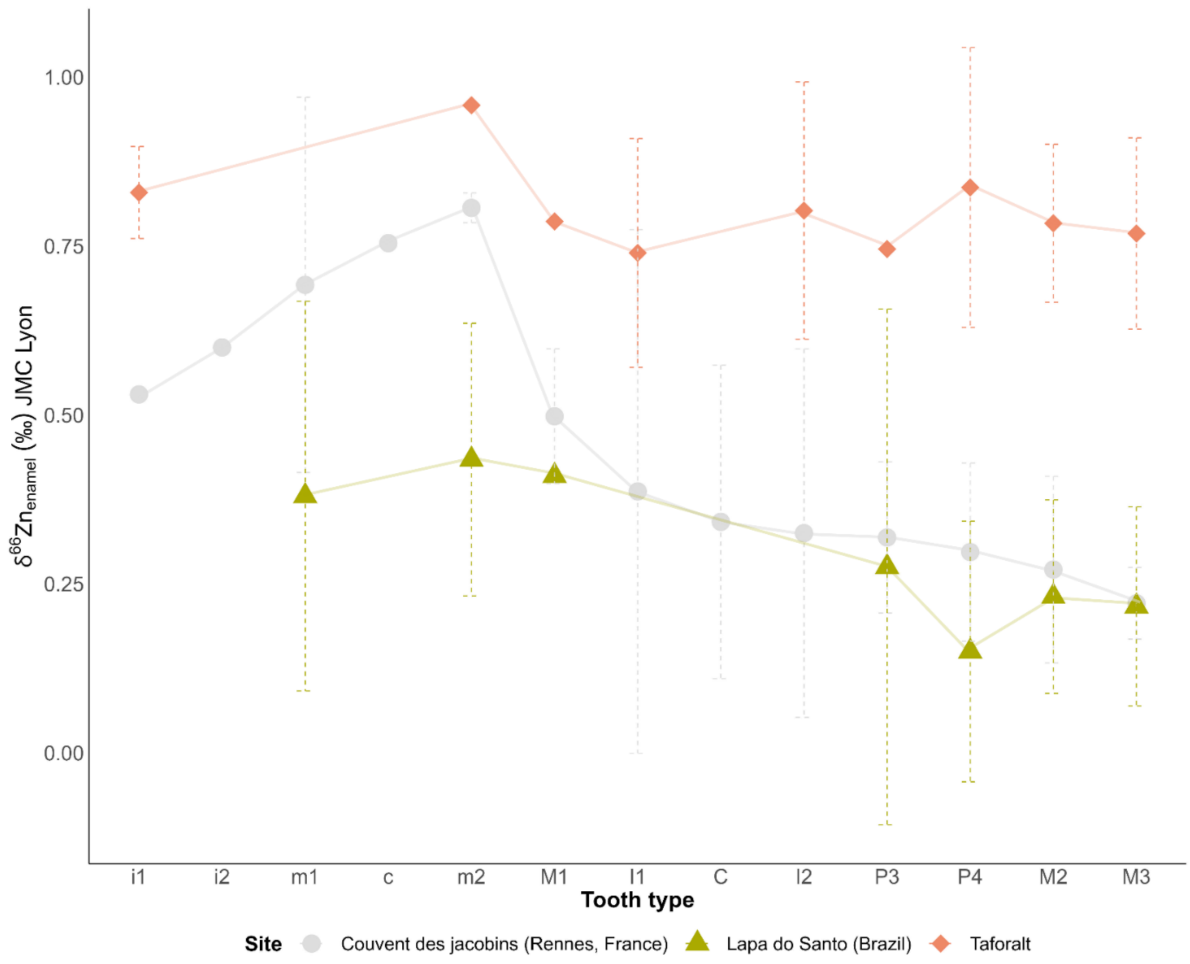

Supplementary Figure 13: The average Zn isotope ratios per dental specimen from Taforalt ($n_{samples}$= 24) compared to those from the Medieval site of Rennes ($n_{samples}$= 35), France[45] and Lapa do Santo ($n_{samples}$= 26), Brazil[48]. The teeth are ranked from the earliest forming enamel to the latest forming crown. The standard deviation for the zinc value in a tooth is represented in dashed line. Each point represents the average zinc values of the same type of tooth.

For δ^{15}N, the difference between breastfeeding and post-weaning period is clearer than with δ^{66}Zn (Supplementary Figure 14), but still lower (1.65‰) than what is usually observed (2-3‰) for δ^{15}N[99,100]. The high TPs of the infants are associated with breastfeeding, which elevates their trophic level above that of the mother[100]. However, the TP of the deciduous second molar of Ind.6 indicates the individual was breastfed during the formation time of the root which was incomplete and then stopped at his death, while that of the rib bone recorded a lower TP probably indicating the introduction of solid food to his diet before death (12 months) since the rib cage has a faster turnover than the other bones (Figure 4, Main paper). It means that breastfeeding had already been strongly decreased or the child was weaned shortly before his death, but lived at least two weeks

after this change. This decrease in trophic level was also detected on the bulk $\delta^{15}N_{collagen}$ (Supplementary Figure 14).

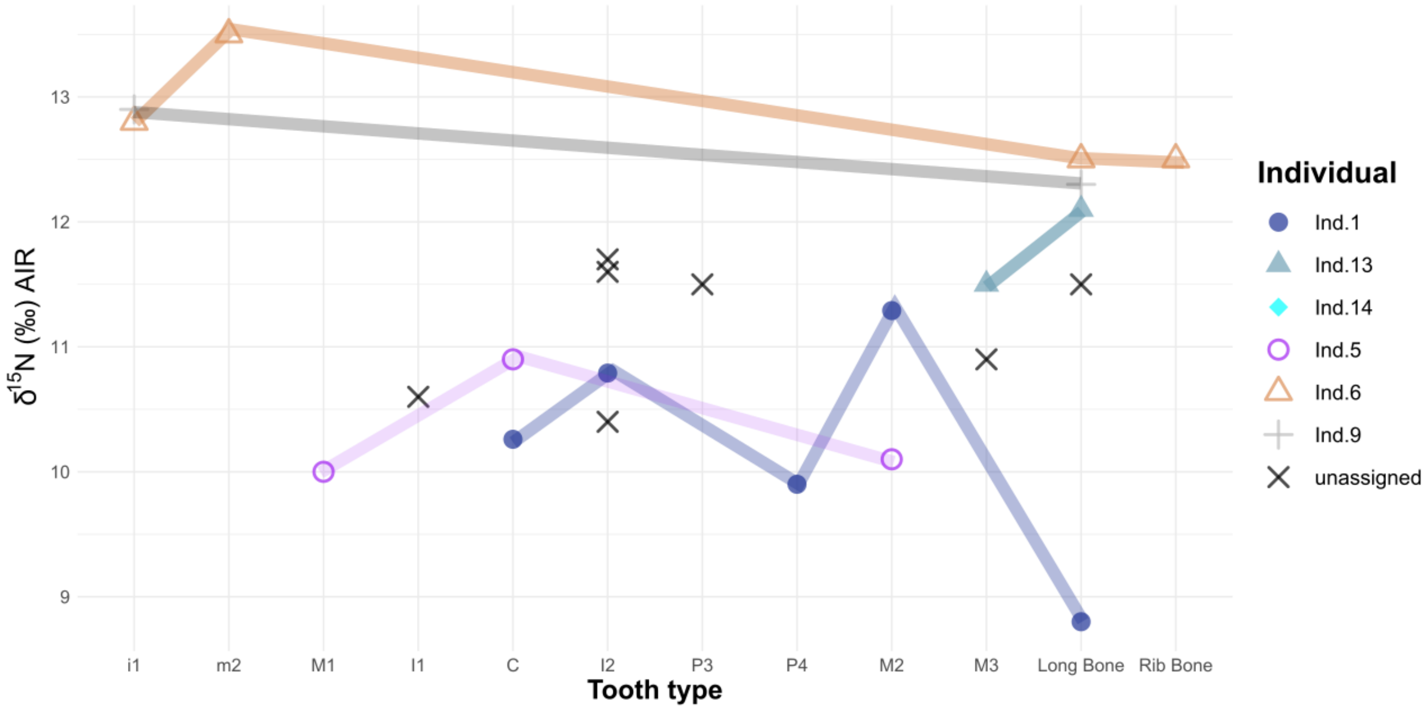

Supplementary Figure 14: The $\delta^{15}N_{collagen}$ values from dentine and bone bulk collagen of different individuals analyzed in this study. The teeth are ranked from the earliest forming to the latest forming the standard deviation for $\delta^{15}N_{collagen}$ is represented in a dashed line. The data from the same individual are connected with a line.

5.2 Trophic level prediction with Zn

The CSIA of amino acids (CSIA-AA) are powerful techniques to reconstruct the trophic position of individuals using the TP (C3) equation[34]. However, since only a few samples yielded enough preserved collagen, we were able to estimate the TP of only 25 samples. Given the fact that Zn is preserved in all the analyzed samples and shows a strong correlation with the TP (on amino acids), we established an equation to predict the TP of samples that did not yield enough collagen using the $\delta^{66}Zn$ values:

$$TP_{Zn}\ (\text{equation}) = -0.8959*\delta^{66}Zn_{enamel} + 3.1455$$

The average value of the human from Taforalt was 2.5 using the equation above while the TP_{AA} equation calculated from amino acids give an average of 2.4 for the adult humans. Because the

influence of the local geology on Zn isotope ratios and notable baseline differences between tropical and non-tropical sites [38,46], this equation is possibly only valid for sites with similar biomes and geologies. For example, applying the equation to terrestrial mammals from Tam Hay Marklot, it predicts a TL of 3 for carnivores, a TL of 2.8 for omnivores and a TL of 2.5 for herbivores [38]. Applying the same equation to terrestrial fauna from Rennes gives a TL= 2.6 for carnivores and 2.3 for herbivores while it places humans at a TP of 2.8 confirming the intensive animal food consumption in this site[45].

Supplementary Table 13: Trophic position estimation of human and animals from Taforalt based on the δ^{66}Zn following the established TP equation mentioned above.

SEVA	***Site***	***Species***	***Zn***	***TP (CSIA)***	***TP (Zn)***
**35819**	Taforalt	Barbary sheep	1.05		2.2
**35821**	Taforalt	Barbary sheep	1.26	2.1	2.0
**35830**	Taforalt	Barbary sheep	0.82	2.1	2.4
**35831**	Taforalt	Barbary sheep	1.16		2.1
**35824**	Taforalt	Hartebeest	1.22	2.2	2.1
**35833**	Taforalt	Gazelle	1.10	2.0	2.2
**35820**	Taforalt	Equid	1.32	2.0	2.0
**35822**	Taforalt	Equid	1.34	1.7	1.9
**35825**	Taforalt	Equid	1.38	1.7	1.9
**35826**	Taforalt	Equid	1.34	2.1	1.9
**35829**	Taforalt	Gazelle	0.85	1.9	2.4
**35835**	Taforalt	Gazelle	1.08	2.0	2.2
**35823**	Taforalt	Hare		2.3	
**35827**	Taforalt	Hartebeest	1.26	2.3	2.0
**35836**	Taforalt	Rhinoceros	1.10	2.3	2.2
**34565**	Taforalt	Equid	1.14		2.1
**35828**	Taforalt	Red fox	0.61	2.9	2.6
**35832**	Taforalt	Gazelle	1.24		2.0

**35834**	Taforalt	Canid	0.42	2.7	2.8
**34566**	Taforalt	Barbary sheep	0.82		2.4
**35961**	Taforalt	Adult human	0.83	2.6	2.4
**35962**	Taforalt	Adult human	0.71		2.5
**35967**	Taforalt	Adult human	0.78		2.4
**35970**	Taforalt	Adult human	0.76		2.5
**35979**	Taforalt	Adult human	0.76		2.5
**35985**	Taforalt	Adult human		2.2	
**35963.A**	Taforalt	Adult human	0.75		2.5
**35963.B**	Taforalt	Adult human	0.83		2.4
**35981**	Taforalt	Adult human	0.92		2.3
**35977**	Taforalt	Adult human	0.76		2.5
**35978**	Taforalt	Adult human	0.74		2.5
**35971.A**	Taforalt	Adult human	0.80		2.4
**35971.B**	Taforalt	Adult human	0.79		2.4
**35975**	Taforalt	Adult human	0.85	2.6	2.4
**35975**	Taforalt	Adult human			
**35980**	Taforalt	Adult human			
**35959**	Taforalt	Adult human			
**35959**	Taforalt	Adult human		2.7	
**35960**	Taforalt	Adult human	0.81	2.2	2.4
**35968**	Taforalt	Adult human	0.75	2.5	2.5
**35969**	Taforalt	Adult human	0.92	2.4	2.3
**35972**	Taforalt	Adult human	0.75	2.5	2.5
**35973**	Taforalt	Adult human	0.69	2.6	2.5
**35974**	Taforalt	Adult human	0.71		2.5
**35976**	Taforalt	Adult human	0.81	2.5	2.4

**34561**	Taforalt	Adult human	0.68		2.5

5.3 Enamel hypoplasia in the human teeth from Taforalt

For zinc isotope analyses, the teeth enamel of the human from Taforalt were sampled in several parts in order to detect possible dietary variation during the enamel formation (Supplementary Figure 13). The majority of the teeth show hypoplasia located on the enamel or on the root of the tooth.

Enamel hypoplasia occurs as linear or pitted deformation that represent disruptions during the formation of enamel.

Our results show that there is no correlation between the δ^{66}Znvalues recorded on the part of enamel with hypoplasia and the part with no hypoplasia (Kruskal-Wallis chi-squared = 0.140, df = 1, p-value = 0.7087) (Supplementary Figure 15). Enamel hypoplasia is sometimes hypothesized as a sign of malnutrition. Low intake of vitamin D from diet and the lack of exposure to sunlight could result in the presence of EH [114]δ. The site is not located in high latitude where the exposure to sunlight is limited in winter, therefore the most likely explanation for this anomaly among the population of Taforalt could be related to the fact that the individuals did not consumed a lot of foods rich in vitamin D such as meat, liver, fish and dairy products. This goes in line with our isotopic results. Studies on modern populations have found that people of low socio-economic status have a higher prevalence of enamel hypoplasia than those of higher social status population[115,116]. This is because the latter had less childhood stress than the former group. In archaeological samples there is an increase incidence in EH during the transition to agriculture, associated with a nutrient-poor diet and an increased reliance on cereals[117].

It is important to consider that life expectancy during the Paleolithic period was notably lower than by today's standards, estimated at an average of just 33 years[118]. In Taforalt, this is evident as many individuals did not exceed 20 years of age[5], likely due to several factors including the prevalence of infectious diseases[4]. Furthermore, dental issues such as caries, tooth wear, periodontal disease, and the intentional removal of upper central incisors may have impaired mastication. Notably, a study has suggested that the elevated occurrence of oral pathologies in the Taforalt population could be indicative of a generally compromised health status, linked to a high prevalence of disease and increased mortality rates [4]. The absence of a pattern between δ^{66}Zn and the location of EH

could be related to the differential timing between the maturation of the enamel and the timing of the incorporation of zinc and other elements [113,119].

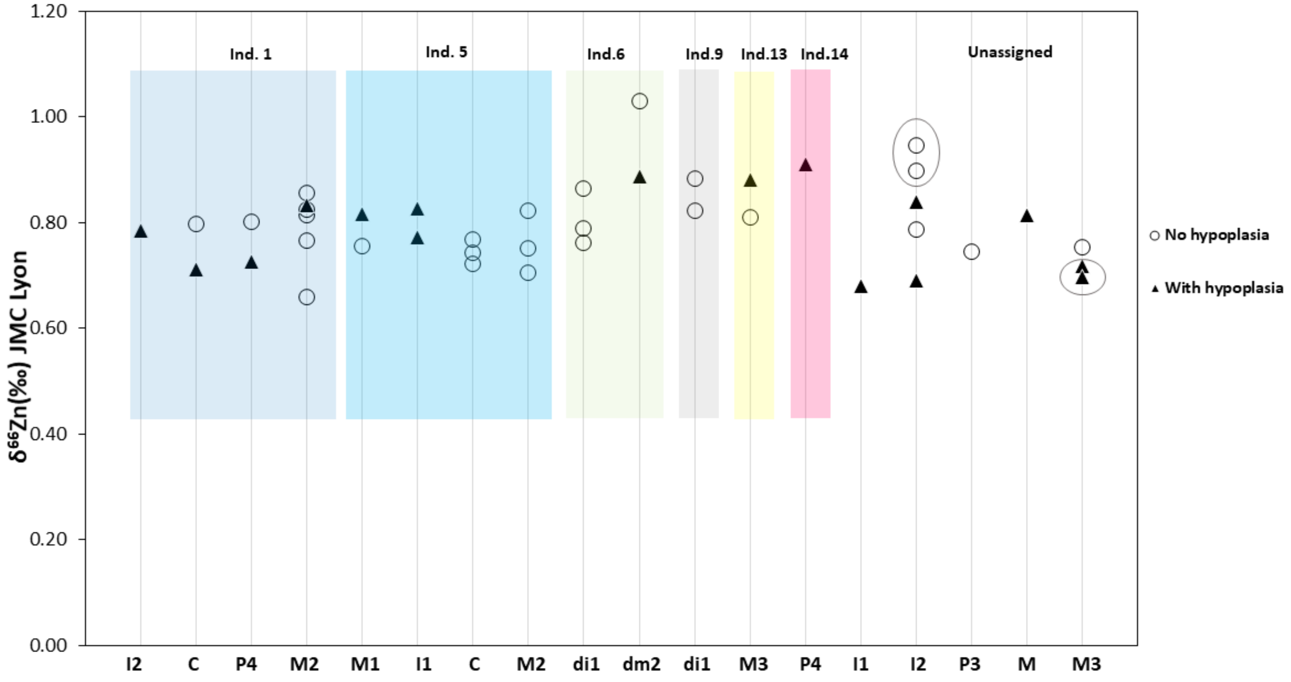

Supplementary Figure 15: The zinc isotope ratio for each human tooth sampled from areas with hypoplasia and with no hypoplasia. For unassigned teeth, the samples belonging to the same tooth are circled in grey. The x-axis corresponds to the tooth type.

5.4 Identified individuals' summaries based on all isotope measurements

Individual 1: a male individual with a plant-based diet

Most of our human samples come from individual 1, which is a young male as revealed by the aDNA[120] . Estimated age at death is 20 years.

The range of enamel analyzed for zinc and strontium for this individual is formed between 1 year (I2) and 8 years (M2). The zinc isotopic ratio does not show any significant changes during this period (0.78±0.05‰) (Extended Data Figure. 3). On the other hand, collagen $\delta^{15}N_{collagen}$ values were obtained from both tooth dentine (5.5-14 years) and bone record different time periods with the latter recording the last years of life due to constant bone remodeling. His bone collagen also shows the lowest $\delta^{15}N_{collagen}$ value of all analyzed samples. The lowest human TP of 2.2 in Taforalt obtained from the bone collagen of this individual, indicates a predominantly plant-based diet (~20 years). To our knowledge, this is the lowest TP value ever recorded in a pre-agriculturist

population. The teeth collagen of this male, formed earlier in life, recorded higher values than his bone collagen suggesting a switch to a plant-based diet shortly before his death. Interestingly, 50% of the remaining teeth of this individual were affected by caries[5]. We should mention that most of this individual's teeth show evidence of hypoplasia on both the crown and the root (See PowerPoint). The observation of the hypoplasia on the teeth included in this study indicates that he suffered from stress from around 3 years (recorded on the I2) until 16 years of age (recorded on his M2). A possible explanation for this transition to a plant-based diet could be related to a health condition that led to nutritional stress.

Individual 5: a female adolescent individual with an intermediate trophic position

Individual 5 is an adolescent female who died between 16 and 18 years of age[5]. The range zinc isotope values from this individual are obtained from teeth formed between birth (M1) and around 8 years (M2) . Zinc isotope ratios also show no significant variation (0.77‰±0.04) similarly to $\delta^{15}N_{collagen}$ values (10.34‰±0.51). We did not obtain the TP from CSIA for this individual because we could not recover enough collagen for the analyses. The TL based on $\delta^{66}Zn$ equation value suggests 60% of plant consumption. We observed the presence of enamel hypoplasia on the teeth of this female indicating that she experienced stresses between 2.5 and 5 years. Caries were present in 57% of the remaining teeth of this individual, in addition to alveolar absorption[4]. The study of the teeth of this individual also shows that this female had the lowest attrition rates among the individuals recovered during the recent excavations[5].

Individual 13: a male individual buried with horn cores and who might have increased his meat consumption over the course of his life

Individual 13 is a male whose age of death is estimated between 18 and 20 years [5]. His M3, which started its formation at the approximated age around of 15 and completed at around 20 years [84] recorded the highest TP (2.6) in the identifiable individuals. This reflects an omnivorous diet with both meat and plant in his diet. His $\delta^{15}N_{collagen}$ is slightly above the values of the other individuals. However, his $\delta^{66}Zn$ from the third molar is relatively high (0.85‰). It is therefore possible that the individual increased his meat consumption after the age of 14.5 years old.

Interestingly, the burial of this individual was associated with seven horn cores of Barbary sheep as funerary artifacts in addition to a freshwater snail shell (*Melanopsis sp.*). In addition, 85% of

his remaining teeth were affected by caries and periodontal diseases[5]. The presence of enamel hypoplasia on his teeth indicates that he suffered from stress at around 13 years of age which coincides with the beginning of the formation of the root of his M3.

Individual 14: a male individual with a plant-based diet

Individual 14 is also a male with an age-at death of 18-20 years[5]. No collagen was preserved in the tooth samples. The tooth enamel of the P4 sampled for the Zn and Sr formed between 3 and 6.5 years, which unlikely reflect breastfeeding. The δ^{66}Zn value of this individual is relatively high (0.92‰,) indicating an important consumption of plant foods. Moreover, a grindstone and a pestle were found in his burial. Caries are present in 42% of the remaining teeth of this individual in addition to alveolar resorption in three teeth. The tooth analyzed for this individual is impressively full of hypoplasia (Supplementary information 7). This period of stress could extend from around 4 to 10 years of age.

Individual 6: a male infant with an early introduction of solid food in diet

Individual 6 is an infant male that died within the first year of his life (6-12 months)[5]. This infant had significant TP change during his short life. The deciduous m2 is formed between 18 weeks *in utero* and 3 years. For this individual the root of this tooth was not totally formed (only 1/3 of the root formed) therefore only reflecting the first year of his life since he died at around 6-12 months. The tooth has a TP of 2.8 clearly reflecting breastfeeding. Similarly, a high value is recorded on his long bone collagen (2.9). It is expected for breastfed infants to be one trophic level above the mother[121]. However, his rib bone collagen TP (2.5) reflects a significant decrease in the trophic level prior to death. Since rib bones have a faster bone remodeling it is possible that they recorded the very last period of his infant life. We suggest that for this individual weaning started before the age of 1 year with an early introduction of adult's foodstuff into his diet. An early weaning is usually unexpected among hunter-gatherer societies as they have prolonged breastfeeding periods due to the lack of suitable food for weaning[122]. We propose that plant food could have been used to wean infants at this site. This sudden change in trophic position was not detected using the $\delta^{15}N_{collagen}$ of the two bones that were analysed for this individual.

The beginning of the formation of his di1 shows a relatively lower δ^{66}Zn value (0.76‰) than the end of the formation of his dm2 (1.03‰). The range of enamel analyzed for zinc and strontium for this individual reflect a formation period between 18 weeks *in utero* (di1) and 10 months (dm2).

We suggest that the deciduous incisor, formed *in utero*, recorded the placental diet coming from the mother, while the dm2 corresponds to an exclusive breastfeeding. A similar pattern was observed in the medieval site from France (Rennes, Jacobins convent) where m2 showed higher δ^{66}Zn values[45].

The CT scan on the deciduous first incisive of this infant showed the presence of hypoplasia along the root (Supplementary information 7, PowerPoint file). This indicates that the stress happened between 4 and 11 months of his life. The fact that he died around the age of 12 months suggests that he may not have survived the cause of this stress.

Individual 9: an infant female with an early solid food introduction

Individual 9 is an infant female as determined by aDNA [5,120] and has an estimated age at death of 5-6 months. The TP from her bone is 2.7 suggesting at least partial breastfeeding but not as high as for Individual 6. The introduction of solid food could therefore have been quite early for this individual too, so that it could be recorded in her bones. No hypoplasia was however detected on the tooth of this individual.

6 Supplementary information 6: Additional tables and figures

6.1 Supplementary figures

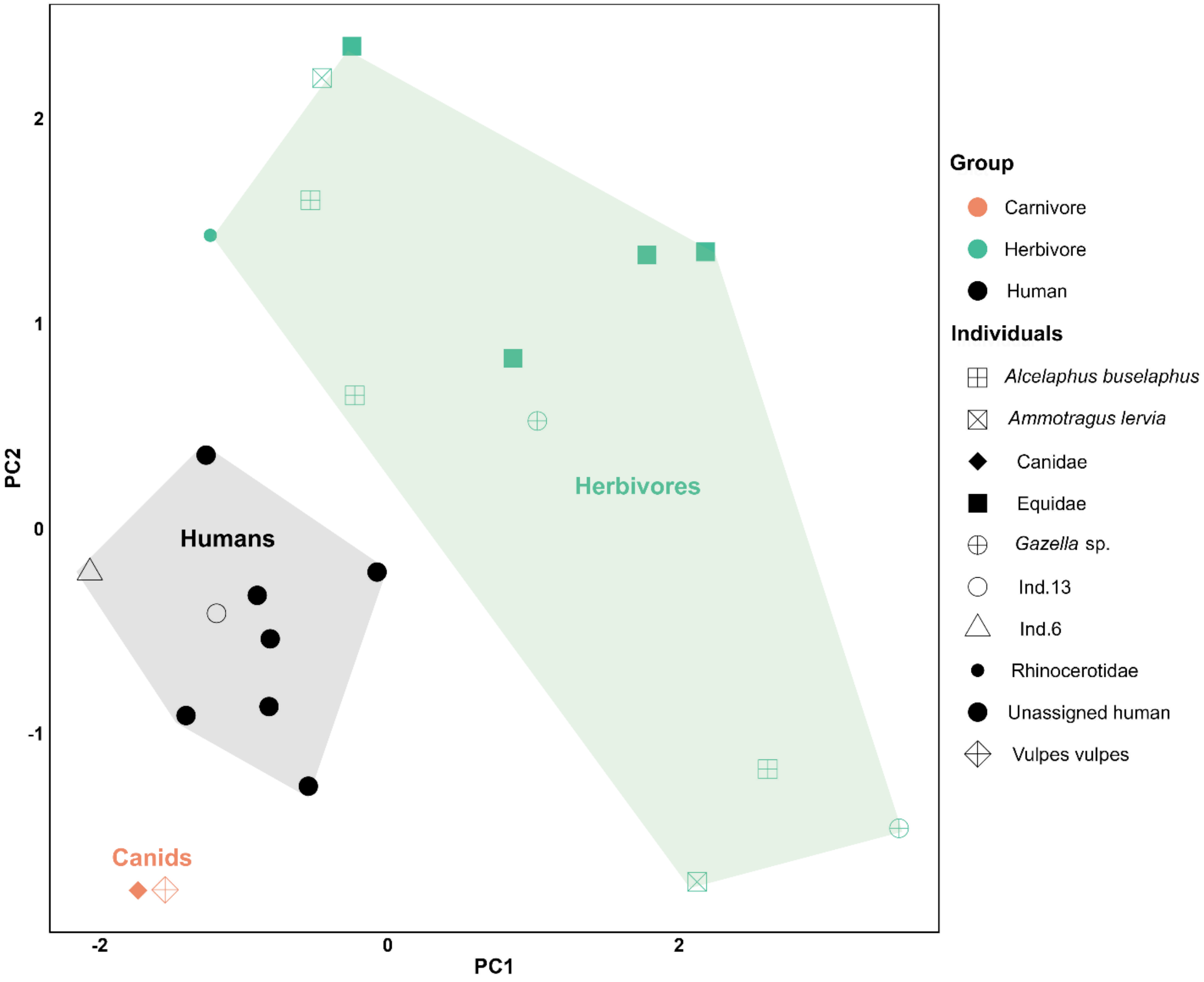

Supplementary Figure 16: PCA plot of $\delta^{13}C$, $\delta^{15}N$, $\delta^{15}N_{Glu}$, $\delta^{15}N_{Phe}$ and $\delta^{66}Zn$ values of the human and faunal remains of Taforalt

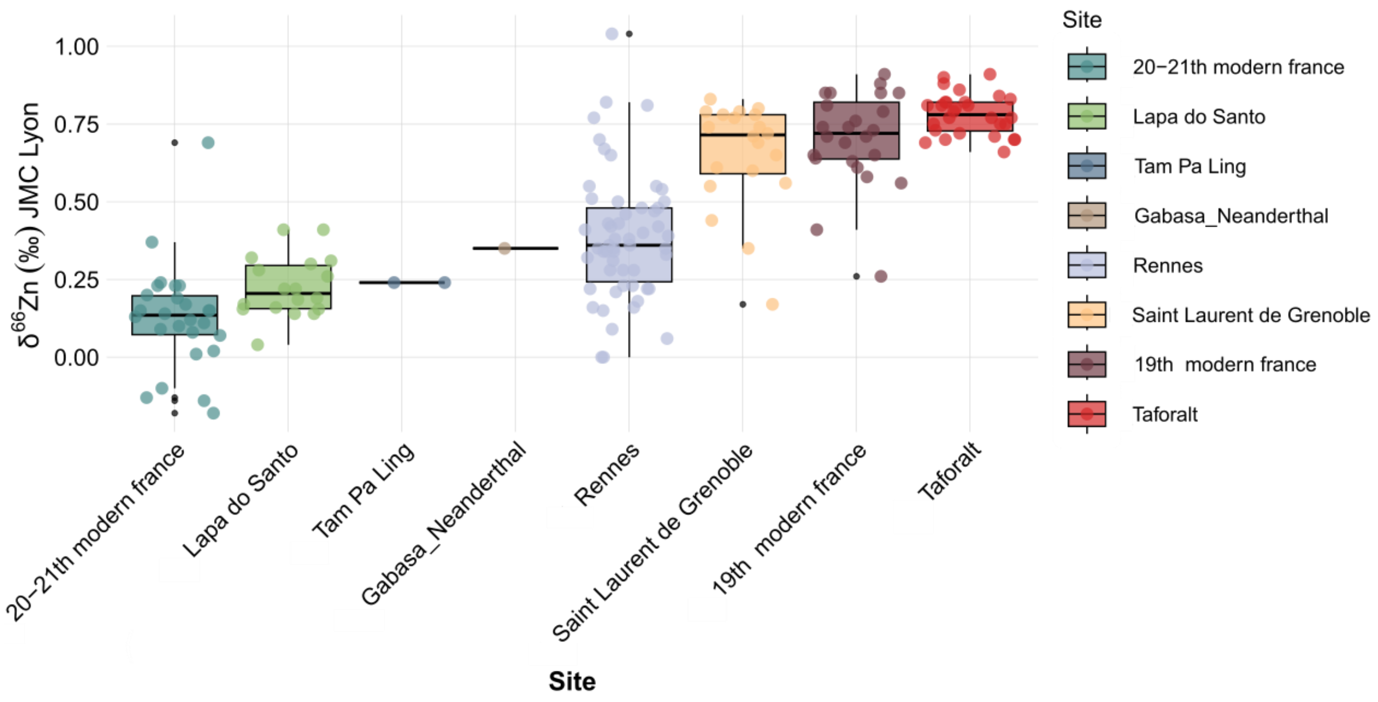

Supplementary Figure 17: Boxplot of $\delta^{66}Zn$ values from Taforalt compared to published data and showing the highest $\delta^{66}Zn$ values recorded for a human population[39,45,48,123,124]. Boxes correspond to the median (centre line) and the first and third quartiles, while whiskers indicate the minimum and maximum values. Each point corresponds to a tooth samples (n= 173).

6.2 Tables of the additional excel file

Supplementary Table 14: Faunal species examined in Taforalt, along with details on their diet and water consumption

Supplementary Table 15: The human individuals from Taforalt included in our study along with their age and gender estimation (ND= Non determined)

Supplementary Table 16: Isotopic data of the human remains from Taforalt and the parts sampled for each isotopic analysis.

Supplementary Table 17: Isotopic data of the faunal remains from Taforalt and the parts sampled for each isotopic analysis.

Supplementary Table 18: The zooarchaeological and the ZooMs identification of the faunal species from Taforalt.

Supplementary Table 19: The mean value of the isotopic results for each human individual from Taforalt analysed in this study.

Supplementary Table 20: The caries and hypoplasia presence in the analyzed teeth of the humans from Taforalt.

Supplementary Table 21: Nitrogen isotope data on amino acids in Taforalt humans and fauna - average and standard deviation

Supplementary Table 22: Carbon isotope data on amino acids in Taforalt humans and fauna - average and standard deviation

Supplementary Table 23: Covariance matrix with the calculated eigenvalues for each of the features used.

Supplementary Table 24: Summary of the number of samples taken per tissue for each isotope analysis

7 Supplementary information 7: Extracts of 3D Scans of the human teeth (pdf)

8 References:

1. Barton, R. N. E. *et al.* Origins of the Iberomaurusian in NW Africa: New AMS radiocarbon dating of the Middle and Later Stone Age deposits at Taforalt Cave, Morocco. *Journal of Human Evolution* **65**, 266–281 (2013).
2. Barton, R. N. E. *et al.* *Cemeteries and Sedentism in the Later Stone Age of NW Africa: Excavations at Grotte Des Pigeons, Taforalt, Morocco*. *Propylaeum* (Monographien des Römisch-Germanischen Zentralmuseums, 2020). doi:10.11588/propylaeum.734.
3. Barton, R. N. E. *et al.* Reconsidering the MSA to LSA transition at Taforalt Cave (Morocco) in the light of new multi-proxy dating evidence. *Quaternary International* **413**, 36–49 (2016).
4. Humphrey, L. T. *et al.* Earliest evidence for caries and exploitation of starchy plant foods in Pleistocene hunter-gatherers from Morocco. *Proceedings of the National Academy of Sciences* **111**, 954–959 (2014).

5. Humphrey, L., Freyne, A., Berridge, P. & Berridge, P. Human burial evidence. in *Cemeteries and Sedentism in the Later Stone Age of NW Africa: Excavations at Grotte des Pigeons, Taforalt, Morocco* (eds. Barton, R. N. E., Bouzouggar, A., Collcutt, S. N. & Humphrey, L. T.) 444–482 (Monographien des Römisch-Germanischen Zentralmuseums, 2020). doi:10.11588/propylaeum.734.
6. Humphrey, L. *et al.* Infant funerary behavior and kinship in Pleistocene hunter-gatherers from Morocco. *Journal of Human Evolution* **135**, 102637 (2019).
7. Bouzouggar, A. *et al.* Reevaluating the Age of the Iberomaurusian in Morocco. *The African Archaeological Review* **25**, 3–19 (2008).
8. Hogue, J. & Barton, N. New radiocarbon dates for the earliest Later Stone Age microlithic technology in Northwest Africa. *Quaternary International* (2016) doi:10.1016/j.quaint.2015.11.144.
9. Barton, N. *et al. Human Burial Evidence from Hattab II Cave and the Question of Continuity in Late Pleistocene–Holocene Mortuary Practices in Northwest Africa*. *Cambridge Archaeological Journal* vol. 18 (2008).
10. Barton, R. N. E. *et al.* The Late Upper Palaeolithic Occupation of the Moroccan Northwest Maghreb During the Last Glacial Maximum. *Afr Archaeol Rev* **22**, 77–100 (2005).
11. Turner, E. Large mammalian faunal assemblages. in *Cemeteries and Sedentism in the Later Stone Age of NW Africa: Excavations at Grotte des Pigeons, Taforalt, Morocco* (eds. Barton, R. N. E., Bouzouggar, A., Collcutt, S. N. & HUMPHREY, L.) 239–308 (Monographien des Römisch-Germanischen Zentralmuseums, 2020). doi:10.11588/propylaeum.734.
12. Arambourg, C., Boule, M., Valois, H. & Verneau, R. *Les Grottes Paléolithiques Des Beni-Segoual (Algérie)*. (Masson, 1934).

13. Hachi, S. Résultats des fouilles récentes d'Afalou Bou Rmel (Bédjaia, Algérie). in *El món mediterrani després del pleniglacial (18000-12000 BP)* 77–92 (Museo de Arqueología de Cataluña = Museu d'Arqueologia de Catalunya, 1997).
14. Desmond, A. *et al.* ZooMS identification of bone tools from the North African Later Stone Age. *Journal of Archaeological Science* **98**, 149–157 (2018).
15. Minagawa, M. & Wada, E. Stepwise enrichment of 15N along food chains: Further evidence and the relation between δ15N and animal age. *Geochimica et Cosmochimica Acta* **48**, 1135–1140 (1984).
16. Schoeninger, M. J. & DeNiro, M. J. Nitrogen and carbon isotopic composition of bone collagen from marine and terrestrial animals. *Geochimica et Cosmochimica Acta* **48**, 625–639 (1984).
17. Ambrose, S. H. & DeNiro, M. J. Reconstruction of African human diet using bone collagen carbon and nitrogen isotope ratios. *Nature* **319**, 321–324 (1986).
18. O'Connell, T., Kneale, C., Tasevska, N. & Kuhnle, G. The diet-body offset in human nitrogen isotopic values: a controlled dietary study. *Am J Phys Anthropol* **149**, 426–434 (2012).
19. Schwarcz, H. P. & Schoeninger, M. J. Stable isotope analyses in human nutritional ecology. *American Journal of Physical Anthropology* **34**, 283–321 (1991).
20. Van Der Merwe, N. J. & Vogel, J. C. 13C Content of human collagen as a measure of prehistoric diet in woodland North America. *Nature* **276**, 815–816 (1978).
21. Richards, M. P., Pettitt, P. B., Stiner, M. C. & Trinkaus, E. Stable isotope evidence for increasing dietary breadth in the European mid-Upper Paleolithic. *PNAS* **98**, 6528–6532 (2001).

22. Drucker, D. & Bocherens, H. Carbon and nitrogen stable isotopes as tracers of change in diet breadth during Middle and Upper Palaeolithic in Europe. *International Journal of Osteoarchaeology* **14**, 162–177 (2004).

23. van Klinken, G. J. Bone Collagen Quality Indicators for Palaeodietary and Radiocarbon Measurements. *Journal of Archaeological Science* **26**, 687–695 (1999).

24. Ambrose, S. H. Preparation and characterization of bone and tooth collagen for isotopic analysis. *Journal of Archaeological Science* **17**, 431–451 (1990).

25. Corr, L. T., Sealy, J. C., Horton, M. C. & Evershed, R. P. A novel marine dietary indicator utilising compound-specific bone collagen amino acid δ13C values of ancient humans. *Journal of Archaeological Science* **32**, 321–330 (2005).

26. Hedges, R. E. M. Isotopes and red herrings: comments on Milner et al. and Lidén et al. *Antiquity* **78**, 34–37 (2004).

27. Heaton, T. H. E., Vogel, J. C., von la Chevallerie, G. & Collett, G. Climatic influence on the isotopic composition of bone nitrogen. *Nature* **322**, 822–823 (1986).

28. Schwarcz, H. P. & Schoeninger, M. J. Stable Isotopes of Carbon and Nitrogen as Tracers for Paleo-Diet Reconstruction. in *Handbook of Environmental Isotope Geochemistry: Vol I* (ed. Baskaran, M.) 725–742 (Springer, Berlin, Heidelberg, 2012). doi:10.1007/978-3-642-10637-8_34.

29. Deniro, M. J. & Epstein, S. Influence of diet on the distribution of nitrogen isotopes in animals. *Geochimica et Cosmochimica Acta* **45**, 341–351 (1981).

30. Virginia, R. A. & Delwiche, C. C. Natural 15N abundance of presumed N2-fixing and non-N2-fixing plants from selected ecosystems. *Oecologia* **54**, 317–325 (1982).

31. Szpak, P., White, C. D., Longstaffe, F. J., Millaire, J.-F. & Sánchez, V. F. V. Carbon and Nitrogen Isotopic Survey of Northern Peruvian Plants: Baselines for Paleodietary and Paleoecological Studies. *PLOS ONE* **8**, e53763 (2013).
32. Codron, J. *et al.* Taxonomic, anatomical, and spatio-temporal variations in the stable carbon and nitrogen isotopic compositions of plants from an African savanna. *Journal of Archaeological Science* **32**, 1757–1772 (2005).
33. Ohkouchi, N. *et al.* Advances in the application of amino acid nitrogen isotopic analysis in ecological and biogeochemical studies. *Organic Geochemistry* **113**, 150–174 (2017).
34. Chikaraishi, Y., Ogawa, N. & Ohkouchi, N. Further evaluation of the trophic level estimation based on nitrogen isotopic composition of amino acids. *Earth, Life, and Isotopes* 37–51 (2010).
35. Honch, N. V., McCullagh, J. S. O. & Hedges, R. E. M. Variation of bone collagen amino acid δ13c values in archaeological humans and fauna with different dietary regimes: Developing frameworks of dietary discrimination. *American Journal of Physical Anthropology* **148**, 495–511 (2012).
36. Colonese, A. C. *et al.* Long-Term Resilience of Late Holocene Coastal Subsistence System in Southeastern South America. *PLOS ONE* **9**, e93854 (2014).
37. McCormack, J. *et al.* Trophic position of Otodus megalodon and great white sharks through time revealed by zinc isotopes. *Nature Communications* **13**, 1–10 (2022).
38. Bourgon, N. *et al.* Zinc isotopes in Late Pleistocene fossil teeth from a Southeast Asian cave setting preserve paleodietary information. *PNAS* **117**, 4675–4681 (2020).

39. Jaouen, K. *et al.* A Neandertal dietary conundrum: Insights provided by tooth enamel Zn isotopes from Gabasa, Spain. *Proceedings of the National Academy of Sciences* **119**, e2109315119 (2022).
40. Tacail, T. *et al.* Calcium isotopic patterns in enamel reflect different nursing behaviors among South African early hominins. *Science advances* **5**, eaax3250 (2019).
41. Martin, J. E., Tacail, T., Braga, J., Cerling, T. E. & Balter, V. Calcium isotopic ecology of Turkana Basin hominins. *Nature communications* **11**, 1–7 (2020).
42. Martin, J. E., Vance, D. & Balter, V. Magnesium stable isotope ecology using mammal tooth enamel. *Proceedings of the National Academy of Sciences* **112**, 430–435 (2015).
43. Bourgon, N. *et al.* Trophic ecology of a Late Pleistocene early modern human from tropical Southeast Asia inferred from zinc isotopes. *Journal of Human Evolution* **161**, 103075 (2021).
44. Costas-Rodríguez, M., Van Heghe, L. & Vanhaecke, F. Evidence for a possible dietary effect on the isotopic composition of Zn in blood via isotopic analysis of food products by multi-collector ICP-mass spectrometry†. *Metallomics* **6**, 139–146 (2014).
45. Jaouen, K. *et al.* Tracing intensive fish and meat consumption using Zn isotope ratios: evidence from a historical Breton population (Rennes, France). *Sci Rep* **8**, 5077 (2018).
46. Jaouen, K., Beasley, M., Schoeninger, M., Hublin, J.-J. & Richards, M. P. Zinc isotope ratios of bones and teeth as new dietary indicators: results from a modern food web (Koobi Fora, Kenya). *Scientific Reports* **6**, 26281 (2016).
47. Kohn, M. J., Schoeninger, M. J. & Barker, W. W. Altered states: effects of diagenesis on fossil tooth chemistry. *Geochimica et Cosmochimica Acta* **63**, 2737–2747 (1999).

48. Jaouen, K. *et al.* Zinc isotope variations in archeological human teeth (Lapa do Santo, Brazil) reveal dietary transitions in childhood and no contamination from gloves. *PLOS ONE* **15**, e0232379 (2020).
49. Cloquet, C., Carignan, J., Lehmann, M. F. & Vanhaecke, F. Variation in the isotopic composition of zinc in the natural environment and the use of zinc isotopes in biogeosciences: a review. *Anal Bioanal Chem* **390**, 451–463 (2008).
50. Maréchal, C. N., Nicolas, E., Douchet, C. & Albarède, F. Abundance of zinc isotopes as a marine biogeochemical tracer. *Geochemistry, Geophysics, Geosystems* **1**, (2000).
51. Moynier, F., Vance, D., Fujii, T. & Savage, P. The Isotope Geochemistry of Zinc and Copper. *Reviews in Mineralogy and Geochemistry* **82**, 543–600 (2017).
52. McCormack, J. *et al.* Zinc isotopes from archaeological bones provide reliable trophic level information for marine mammals. *Commun Biol* **4**, 1–11 (2021).
53. Jaouen, K., Szpak, P. & Richards, M. P. Zinc Isotope Ratios as Indicators of Diet and Trophic Level in Arctic Marine Mammals. *PLoS One* **11**, e0152299 (2016).
54. Balter, V. *et al.* Bodily variability of zinc natural isotope abundances in sheep. *Rapid Commun Mass Spectrom* **24**, 605–612 (2010).
55. Balter, V. *et al.* Contrasting Cu, Fe, and Zn isotopic patterns in organs and body fluids of mice and sheep, with emphasis on cellular fractionation. *Metallomics* **5**, 1470–1482 (2013).
56. Moynier, F., Fujii, T., Shaw, A. S. & Le Borgne, M. Heterogeneous distribution of natural zinc isotopes in mice. *Metallomics* **5**, 693–699 (2013).
57. Mahan, B., Moynier, F., Jørgensen, A. L., Habekost, M. & Siebert, J. Examining the homeostatic distribution of metals and Zn isotopes in Göttingen minipigs. *Metallomics* **10**, 1264–1281 (2018).

58. Moynier, F. *et al.* Isotopic fractionation and transport mechanisms of Zn in plants. *Chemical Geology* **267**, 125–130 (2009).
59. Viers, J. *et al.* Evidence of Zn isotopic fractionation in a soil–plant system of a pristine tropical watershed (Nsimi, Cameroon). *Chemical Geology* **239**, 124–137 (2007).
60. Fuller, B. T., Richards, M. P. & Mays, S. A. Stable carbon and nitrogen isotope variations in tooth dentine serial sections from Wharram Percy. *Journal of Archaeological Science* **30**, 1673–1684 (2003).
61. Bentley, A. Strontium Isotopes from the Earth to the Archaeological Skeleton: A Review. *J Archaeol Method Theory* **13**, 135–187 (2006).
62. Price, T. D., Burton, J. H. & Bentley, R. A. The Characterization of Biologically Available Strontium Isotope Ratios for the Study of Prehistoric Migration. *Archaeometry* **44**, 117–135 (2002).
63. Marie, P. J., Ammann, P., Boivin, G. & Rey, C. Mechanisms of action and therapeutic potential of strontium in bone. *Calcif Tissue Int* **69**, 121–129 (2001).
64. Craig, O. E., Ross, R., Andersen, S. H., Milner, N. & Bailey, G. N. Focus: sulphur isotope variation in archaeological marine fauna from northern Europe. *Journal of Archaeological Science* **33**, 1642–1646 (2006).
65. Nehlich, O. The application of sulphur isotope analyses in archaeological research: A review. *Earth-Science Reviews* **142**, 1–17 (2015).
66. Rees, C. E., Jenkins, W. J. & Monster, J. The sulphur isotopic composition of ocean water sulphate. *Geochimica et Cosmochimica Acta* **42**, 377–381 (1978).
67. Thode, G., H. Sulphur Isotopes in Nature and the Environment: An Overview. in (2005).

68. Kusakabe, M., Rafter, T. A., Stout, J. D. & Collie, T. W. Sulphur isotopic variations in nature. *New Zealand Journal of Science* **19**, 433–440 (1976).

69. Trust, B. A. & Fry, B. Stable sulphur isotopes in plants: a review. *Plant, Cell & Environment* **15**, 1105–1110 (1992).

70. Richards, M. P., Fuller, B. T., Sponheimer, M., Robinson, T. & Ayliffe, L. Sulphur isotopes in palaeodietary studies: a review and results from a controlled feeding experiment. *International Journal of Osteoarchaeology* **13**, 37–45 (2003).

71. Tcherkez, G. & Tea, I. 32S/34S isotope fractionation in plant sulphur metabolism. *New Phytologist* **200**, 44–53 (2013).

72. Webb, E. C. *et al.* Sulphur-isotope compositions of pig tissues from a controlled feeding study. *STAR: Science & Technology of Archaeological Research* **3**, 71–79 (2017).

73. Wilman, H. *et al.* EltonTraits 1.0: Species-level foraging attributes of the world’s birds and mammals. *Ecology* **95**, 2027–2027 (2014).

74. Mimoun, J. & Nouira, S. Food habits of the aoudad Ammotragus lervia in the Bou Hedma mountains, Tunisia. *South African Journal of Science* **Volume 111**, (2015).

75. Loggers, C. Forage availability versus seasonal diets, as determined by fecal analysis, of dorcas gazelles in Morocco. **55**, 255–268 (1991).

76. Herrera-Sánchez, F. J. *et al.* Identifying priority conservation areas in a Saharan environment by highlighting the endangered Cuvier’s Gazelle as a flagship species. *Sci Rep* **10**, 8241 (2020).

77. Estes, R. *The Behavior Guide to African Mammals : Including Hoofed Mammals, Carnivores, Primates*. (Berkeley : University of California Press, 1991).

78. Gray, G. G. & Simpson, C. D. Ammotragus lervia. *Mammalian Species* 1–7 (1980) doi:10.2307/3504009.

79. Ash, A. *et al.* Regional differences in health, diet and weaning patterns amongst the first Neolithic farmers of central Europe. *Sci Rep* **6**, 29458 (2016).

80. Ogilvie, M. D., Curran, B. K. & Trinkaus, E. Incidence and patterning of dental enamel hypoplasia among the Neandertals. *American Journal of Physical Anthropology* **79**, 25–41 (1989).

81. Clayton, F., Sealy, J. & Pfeiffer, S. Weaning age among foragers at Matjes river rock shelter, South Africa, from stable nitrogen and carbon isotope analyses. *American Journal of Physical Anthropology* **129**, 311–317 (2006).

82. Wright, J. T. Enamel Phenotypes: Genetic and Environmental Determinants. *Genes (Basel)* **14**, 545 (2023).

83. Bocquentin, F. Pratiques funéraires, paramètres biologiques et identités culturelles au Natoufien : une analyse archéo-anthropologique. (Université Bordeaux 1, 2003).

84. AlQahtani, S. j., Hector, M. p. & Liversidge, H. m. Brief communication: The London atlas of human tooth development and eruption. *American Journal of Physical Anthropology* **142**, 481–490 (2010).

85. Reade, H., Stevens, R. E., Barker, G. & O’Connell, T. C. Tooth enamel sampling strategies for stable isotope analysis: Potential problems in cross-method data comparisons. *Chemical Geology* **404**, 126–135 (2015).

86. Bryant, J. D., Froelich, P. N., Showers, W. J. & Genna, B. J. A Tale of Two Quarries: Biologic and Taphonomic Signatures in the Oxygen Isotope Composition of Tooth Enamel Phosphate from Modern and Miocene Equids. *PALAIOS* **11**, 397–408 (1996).

87. Fricke, H. C. & O'Neil, J. R. Inter- and intra-tooth variation in the oxygen isotope composition of mammalian tooth enamel phosphate: implications for palaeoclimatological and palaeobiological research. *Palaeogeography, Palaeoclimatology, Palaeoecology* **126**, 91–99 (1996).
88. Linhart, S. B. Dentition and Pelage in the Juvenile Red Fox (Vulpes vulpes). *Journal of Mammalogy* **49**, 526–528 (1968).
89. Dauphin, Y. & Williams, C. T. Diagenetic trends of dental tissues. *Comptes Rendus Palevol* **3**, 583–590 (2004).
90. Reynard, B. & Balter, V. Trace elements and their isotopes in bones and teeth: Diet, environments, diagenesis, and dating of archeological and paleontological samples. *Palaeogeography, Palaeoclimatology, Palaeoecology* **416**, 4–16 (2014).
91. Weiss, D. J. *et al.* Isotopic discrimination of zinc in higher plants. *New Phytologist* **165**, 703–710 (2005).
92. McCormack, J. *et al.* Combining collagen extraction with mineral Zn isotope analyses from a single sample for robust palaeoecological investigations. *Archaeol Anthropol Sci* **14**, 137 (2022).
93. Bataille, C. P. *et al.* Triple sulfur-oxygen-strontium isotopes probabilistic geographic assignment of archaeological remains using a novel sulfur isoscape of western Europe. *PLOS ONE* **16**, e0250383 (2021).
94. Guiry, E. J. & Szpak, P. Quality control for modern bone collagen stable carbon and nitrogen isotope measurements. *Methods in Ecology and Evolution* **11**, 1049–1060 (2020).

95. O’Connell, T. C., Kneale, C. J., Tasevska, N. & Kuhnle, G. G. C. The diet-body offset in human nitrogen isotopic values: a controlled dietary study. *Am J Phys Anthropol* **149**, 426–434 (2012).

96. Bocherens, H. & Drucker, D. Trophic level isotopic enrichment of carbon and nitrogen in bone collagen: case studies from recent and ancient terrestrial ecosystems. *International Journal of osteoarchaeology* **13**, 46–53 (2003).

97. Ambrose, S. H. Controlled Diet and Climate Experiments on Nitrogen Isotope Ratios of Rats. in *Biogeochemical Approaches to Paleodietary Analysis* (eds. Ambrose, S. H. & Katzenberg, M. A.) 243–259 (Springer US, Boston, MA, 2002). doi:10.1007/0-306-47194-9_12.

98. Lee-Thorp, J., Vaughan, A., Ditchfield, P. & Humphrey, L. T. Isotope ecology of the Sector 10 burials. in *Propylaeum* (eds. Barton, R. N. E., Bouzouggar, A., Collcutt, S. N. & Humphrey, L. T.) (Monographien des Römisch-Germanischen Zentralmuseums, 2020). doi:10.11588/propylaeum.734.

99. Fahy, G. E., Deter, C., Pitfield, R., Miszkiewicz, J. J. & Mahoney, P. Bone deep: variation in stable isotope ratios and histomorphometric measurements of bone remodelling within adult humans. *Journal of Archaeological Science* **87**, 10–16 (2017).

100. Jaouen, K. *et al.* Exceptionally high δ15N values in collagen single amino acids confirm Neandertals as high-trophic level carnivores. *PNAS* **116**, 4928–4933 (2019).

101. O’Connell, T. C. & Collins, M. J. Comment on “Ecological niche of Neanderthals from Spy Cave revealed by nitrogen isotopes of individual amino acids in collagen” [J. Hum. Evol. 93 (2016) 82–90]. *Journal of Human Evolution* **117**, 53–55 (2018).

102. Ma, Y. *et al.* Aminoisoscapes and palaeodiet reconstruction: New perspectives on millet-based diets in China using amino acid δ13C values. *Journal of Archaeological Science* **125**, 105289 (2021).

103. Cheung, C., Herrscher, E. & Thomas, A. Compound specific isotope evidence points to use of freshwater resources as weaning food in Middle Neolithic Paris Basin. *American Journal of Biological Anthropology* **179**, 118–133 (2022).

104. Choy, K., Smith, C. I., Fuller, B. T. & Richards, M. P. Investigation of amino acid δ13C signatures in bone collagen to reconstruct human palaeodiets using liquid chromatography–isotope ratio mass spectrometry. *Geochimica et Cosmochimica Acta* **74**, 6093–6111 (2010).

105. Mora, A., Pacheco, A., Roberts, C. & Smith, C. Pica 8: Refining dietary reconstruction through amino acid δ13C analysis of tendon collagen and hair keratin. *Journal of Archaeological Science* **93**, 94–109 (2018).

106. Thorp, J. H. & Bowes, R. E. Carbon Sources in Riverine Food Webs: New Evidence from Amino Acid Isotope Techniques. *Ecosystems* **20**, 1029–1041 (2017).

107. Chikaraishi, Y., Ogawa, N. & Ohkouchi, N. Further evaluation of the trophic level estimation based on nitrogen isotopic composition of amino acids. *Earth, Life, and Isotopes* 37–51 (2010).

108. Drucker, D. G. *et al.* Isotopic analyses suggest mammoth and plant in the diet of the oldest anatomically modern humans from far southeast Europe. *Sci Rep* **7**, 6833 (2017).

109. Naito, Y. I. *et al.* Ecological niche of Neanderthals from Spy Cave revealed by nitrogen isotopes of individual amino acids in collagen. *Journal of Human Evolution* **93**, 82–90 (2016).

110. Jhala, Y. & Moehlman, P. Golden jackal (Canis aureus). *Canids: Foxes, Wolves, Jackals and Dogs* 156–161 (2004).

111. Drake, N. A., Eckardt, F. D. & White, H., Kevin. Sources of sulphur in gypsiferous sediments and crusts and pathways of gypsum redistribution in southern Tunisia. *Earth Surface Processes and Landforms* **29**, 1459–1471 (2004).

112. Flentje, H. *et al.* Identification and monitoring of Saharan dust: An inventory representative for south Germany since 1997. *Atmospheric Environment* **109**, 87–96 (2015).

113. Passey, B. H. *et al.* Inverse methods for estimating primary input signals from time-averaged isotope profiles. *Geochimica et Cosmochimica Acta* **69**, 4101–4116 (2005).

114. Fulton, A., Amlani, M. & Parekh, S. Oral manifestations of vitamin D deficiency in children. *Br Dent J* **228**, 515–518 (2020).

115. Nakayama, N. The Relationship Between Linear Enamel Hypoplasia and Social Status in 18th to 19th Century Edo, Japan. *International Journal of Osteoarchaeology* **26**, 1034–1044 (2016).

116. Saunders, S. R. & Keenleyside, A. Enamel hypoplasia in a Canadian historic sample. *American Journal of Human Biology* **11**, 513–524 (1999).

117. Bocquentin, F., Chamel, B., Anton, M. & Noûs, C. The Subsistence and Foodways Transition during the Neolithization Process. Glimpses from a Contextualized Dental Perspective. *Food and History* **19**, 23–52 (2021).

118. Goldman, L. Three Stages of Health Encounters Over 8000 Human Generations and How They Inform Future Public Health. *Am J Public Health* **108**, 60–62 (2018).

119. Tacail, T. *et al.* Assessing human weaning practices with calcium isotopes in tooth enamel. *PNAS* **114**, 6268–6273 (2017).

120. van de Loosdrecht, M. *et al.* Pleistocene North African genomes link Near Eastern and sub-Saharan African human populations. *Science* **360**, 548–552 (2018).

121. Fuller, B. t., Fuller, J. l., Harris, D. a. & Hedges, R. e. m. Detection of breastfeeding and weaning in modern human infants with carbon and nitrogen stable isotope ratios. *American Journal of Physical Anthropology* **129**, 279–293 (2006).

122. Richards, M., Pearson, J., Molleson, T., Russell, N. & Martin, L. Stable Isotope Evidence of Diet at Neolithic Çatalhöyük, Turkey. *Journal of Archaeological Science* **30**, (2003).

123. Bourgon, N. *et al.* Trophic ecology of a Late Pleistocene early modern human from tropical Southeast Asia inferred from zinc isotopes. *Journal of Human Evolution* **161**, 103075 (2021).

124. Jaouen, K., Herrscher, E. & Balter, V. Copper and zinc isotope ratios in human bone and enamel. *Am J Phys Anthropol* **162**, 491–500 (2017).

Supplementary Information 7: 3D models of the human teeth and sampling strategy

SEVA: 35965

Ind: 6

URdi1

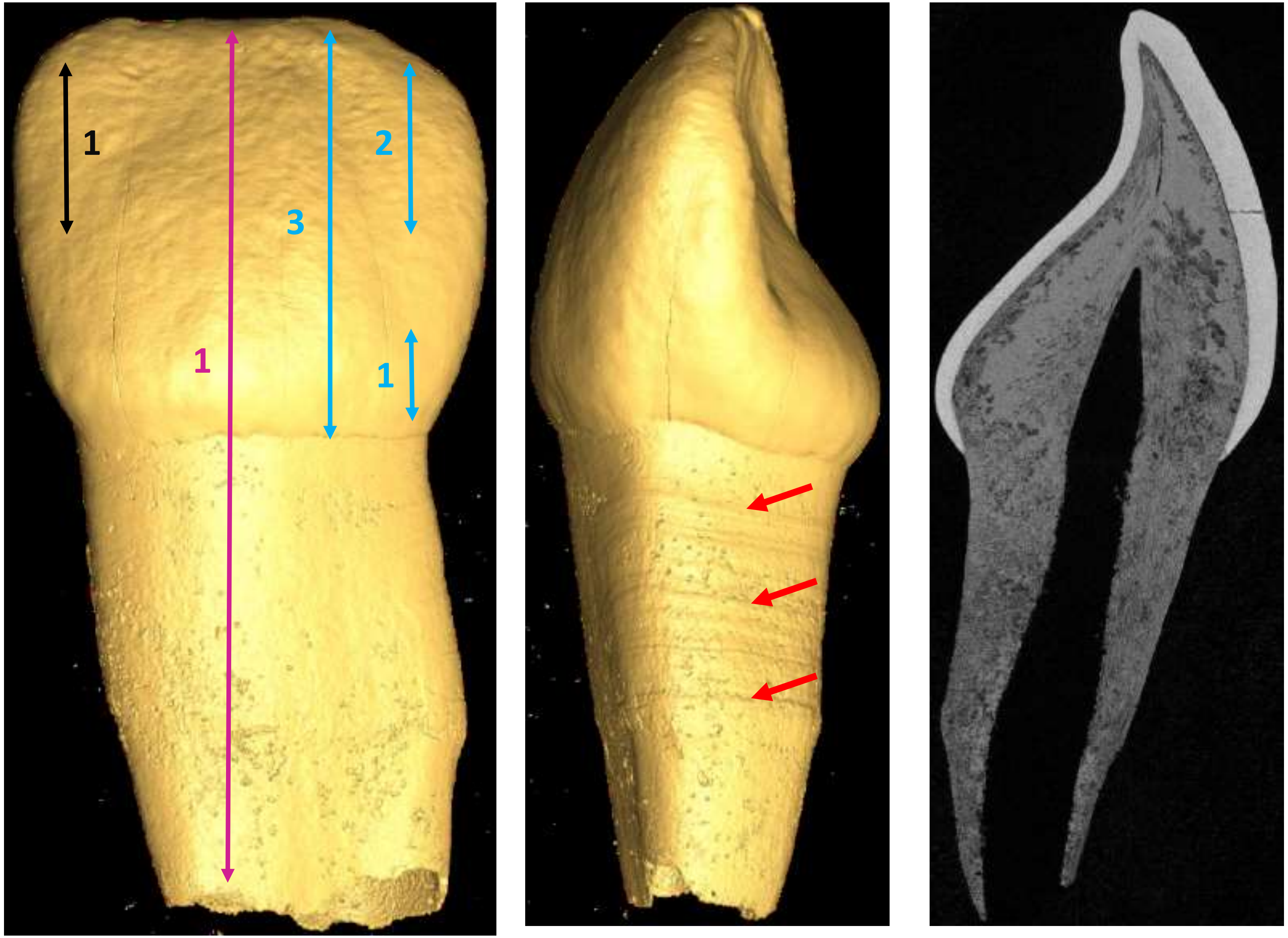

Black: Strontium isotope

Blue: Zinc isotope

Purple: Collagen

Red: Hypoplasia

Note: Many stress on the root

SEVA: 35964

Ind: 6

URdm2

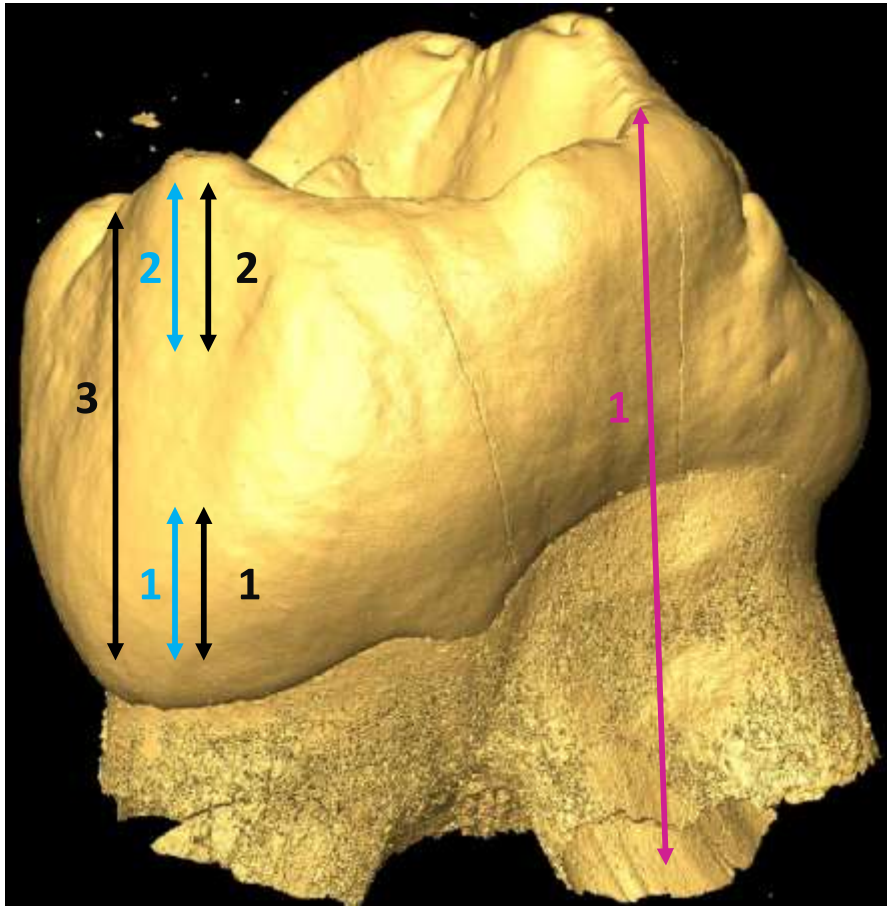

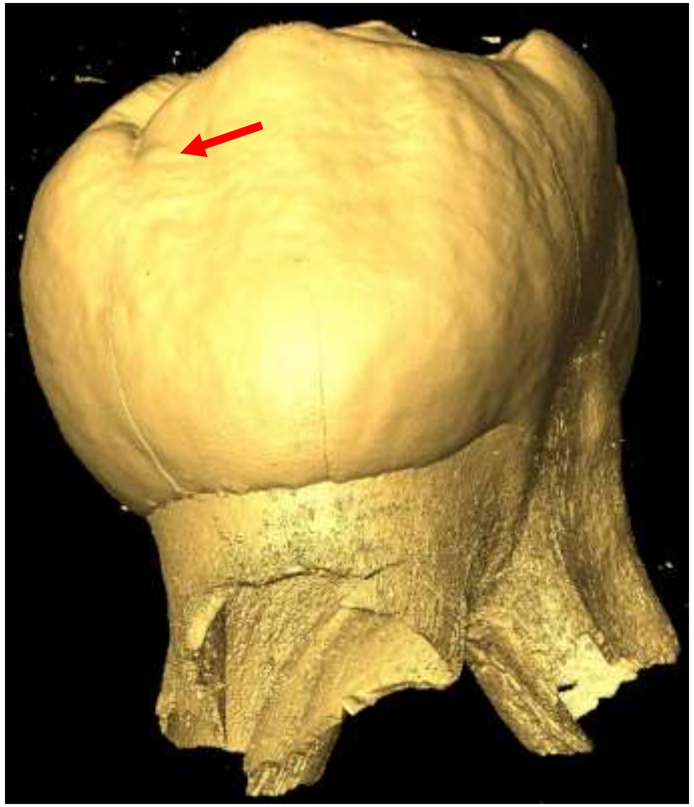

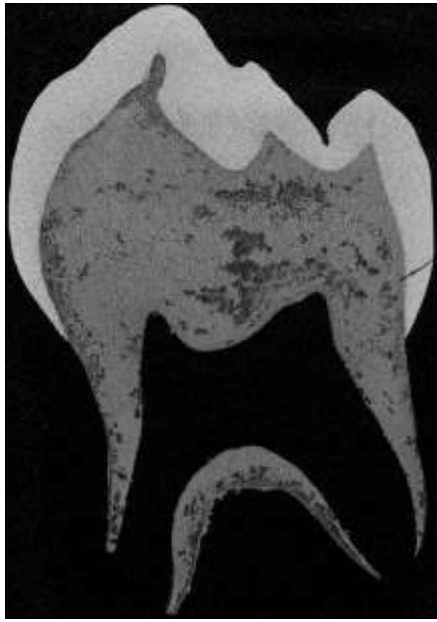

Black: Strontium isotope

Blue: Zinc isotope

Purple: Collagen

Red: Hypoplasia

SEVA: 35960

Ind: Unassigned

_LLI2

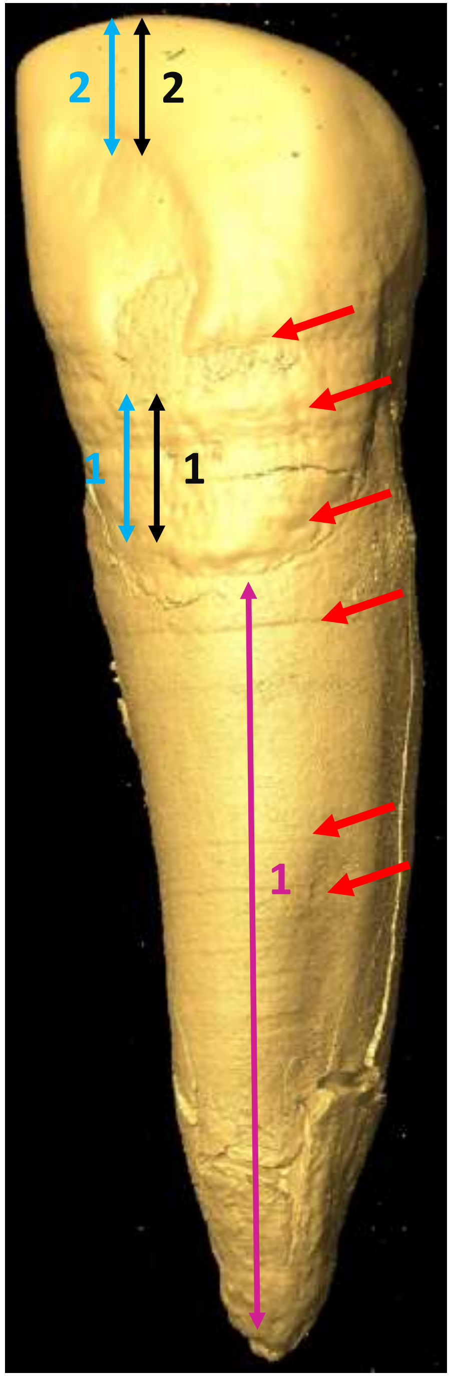

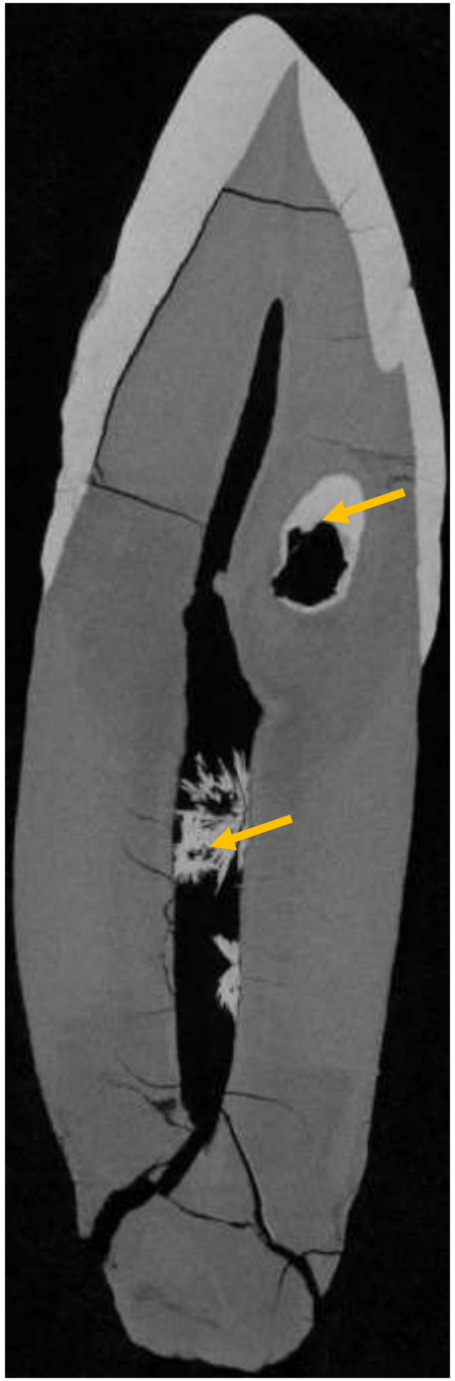

Black: Strontium isotope

Blue: Zinc isotope

Purple: Collagen

Red: Hypoplasia

Yellow: recrystallisation

35963.B

Ind. 1

Black: Strontium isotope

Blue: Zinc isotope

Purple: Collagen

Red: Hypoplasia
Green: Caries

_LLM2

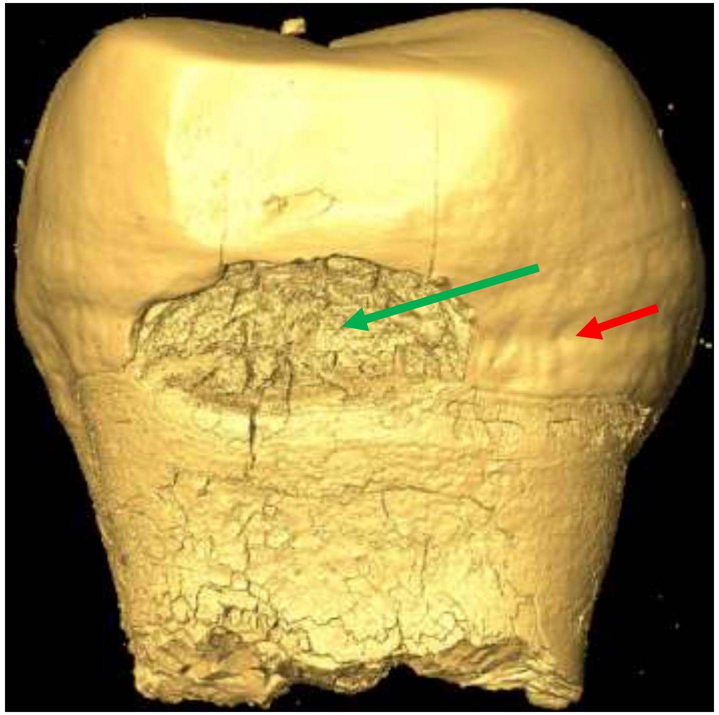

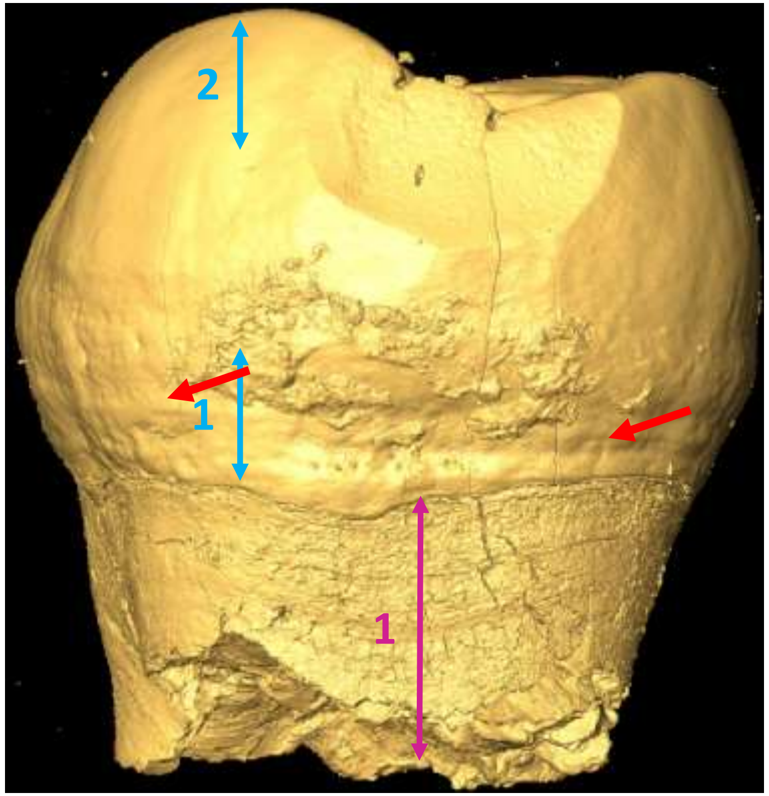

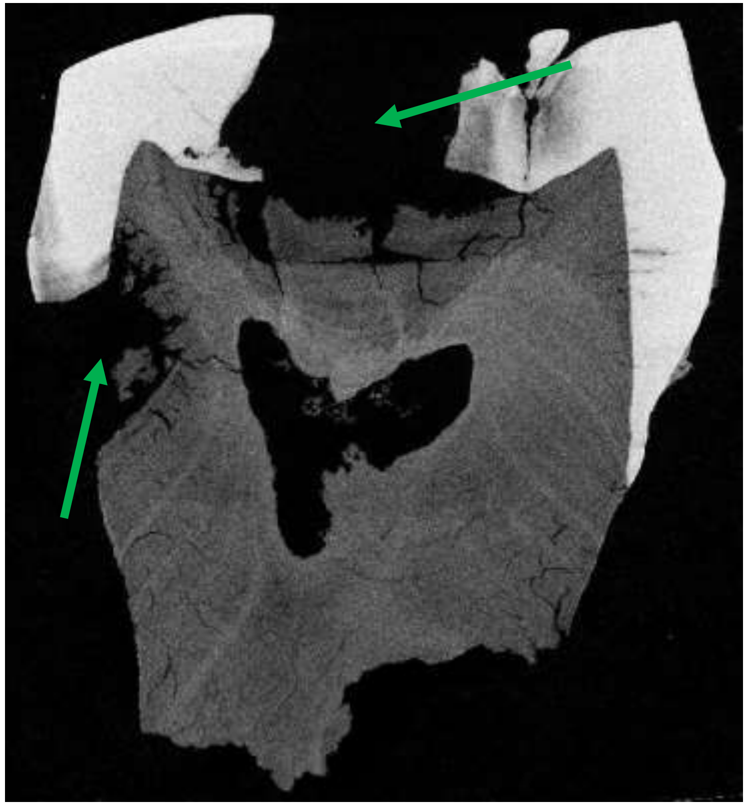

35961

Ind. 1

ULM2

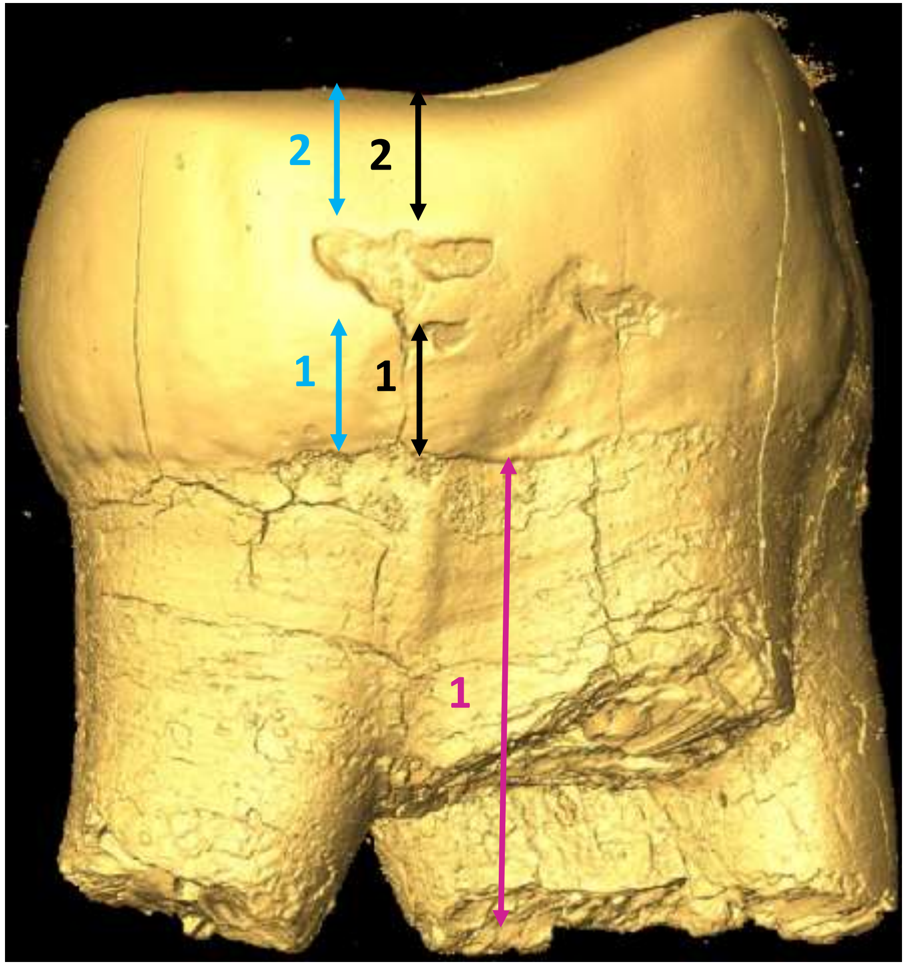

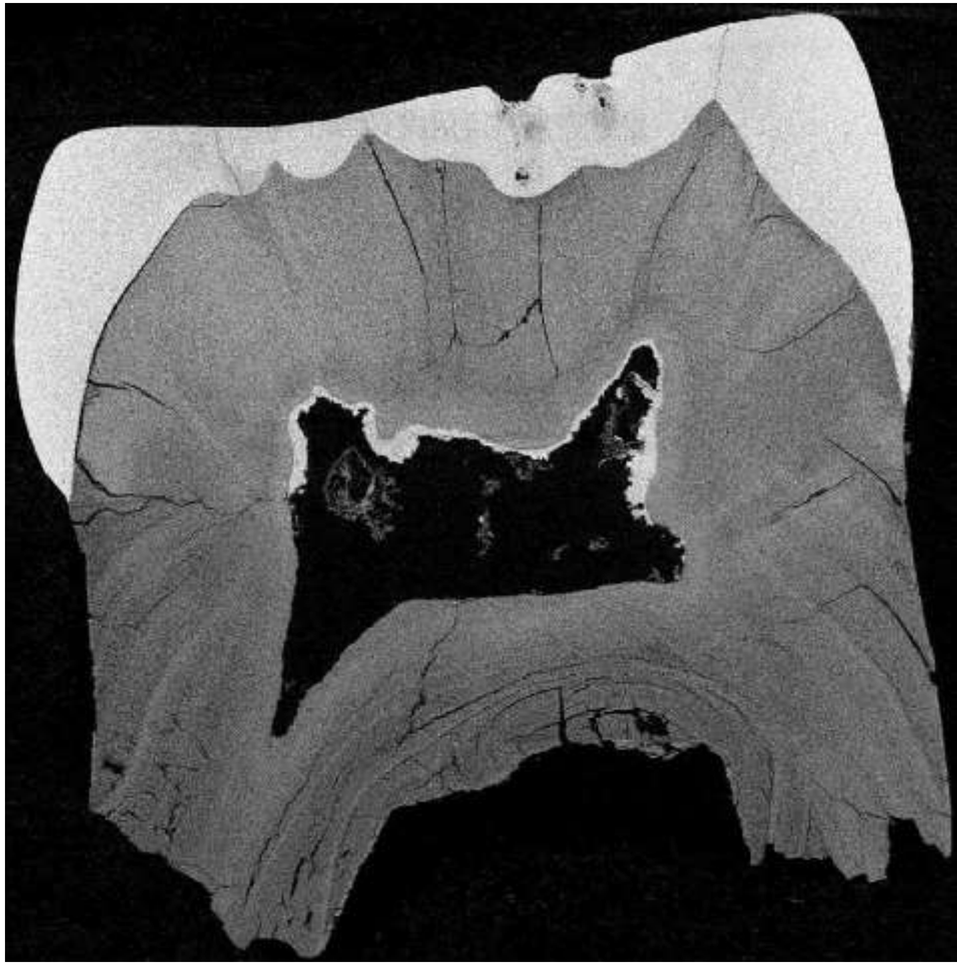

Black: Strontium isotope

Blue: Zinc isotope

Purple: Collagen

Note: Tooth full of stress

35966

Ind. 9

URdi1

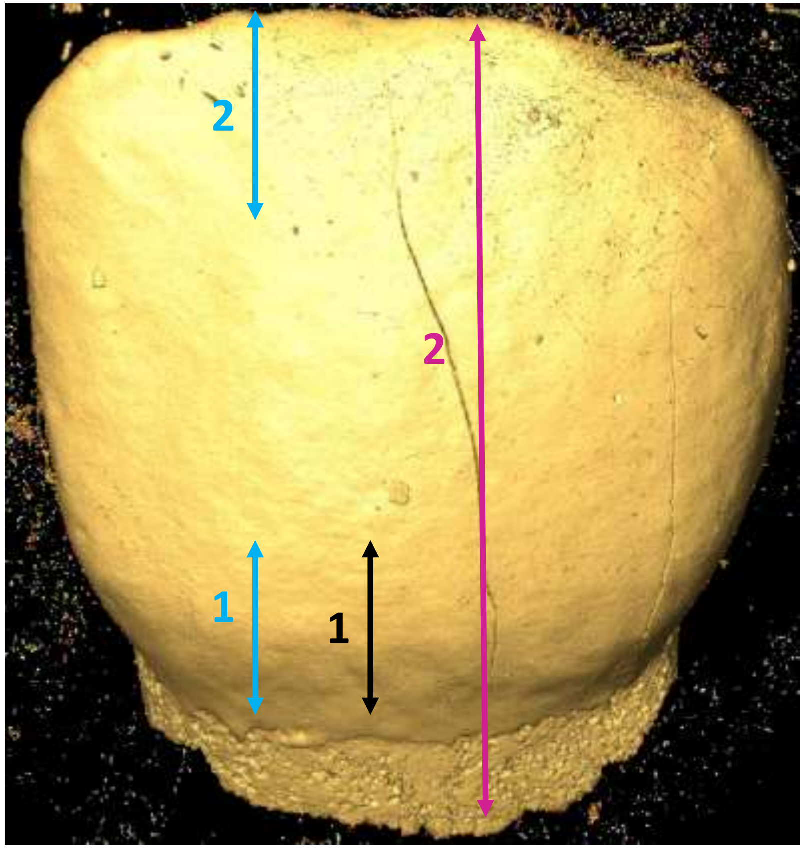

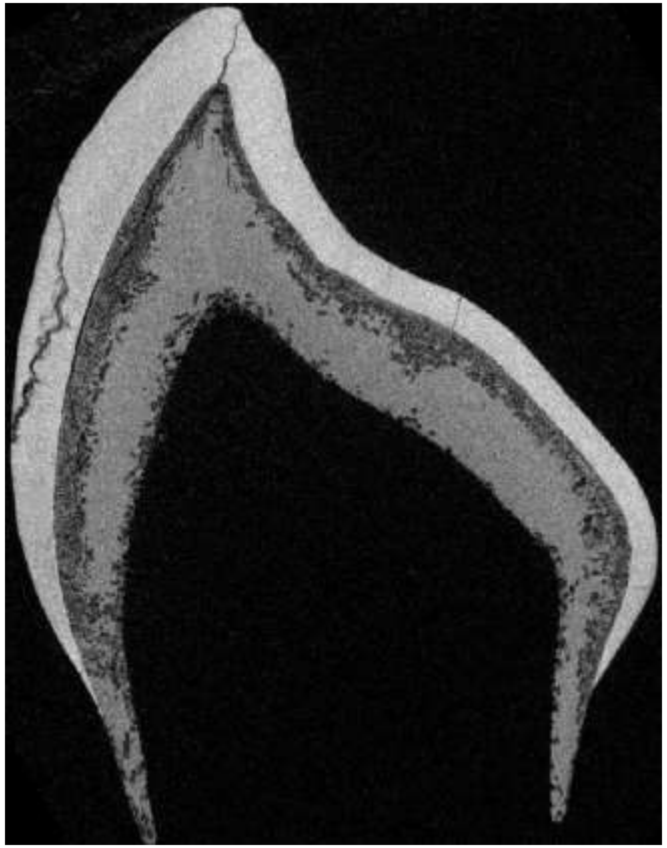

Black: Strontium isotope

Blue: Zinc isotope

Purple: Collagen

35963.A

Ind. 1

LLC

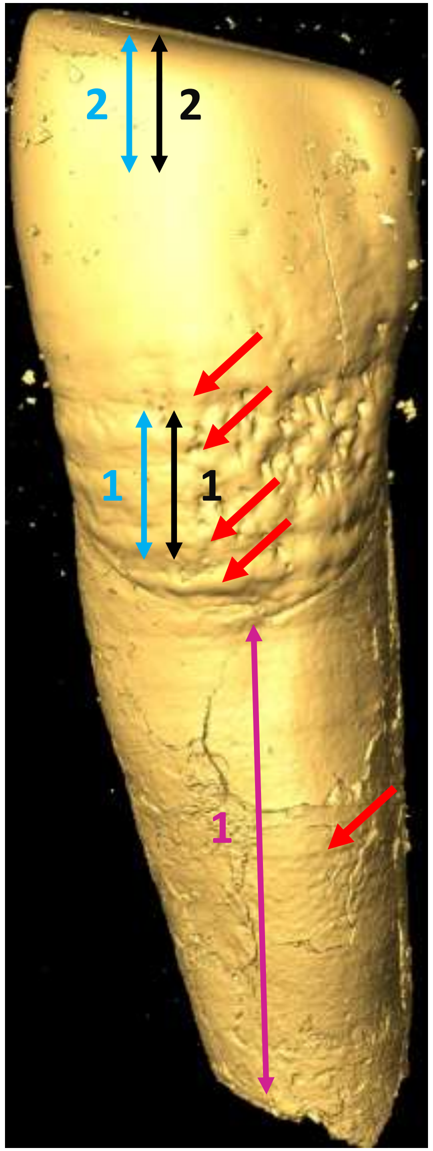

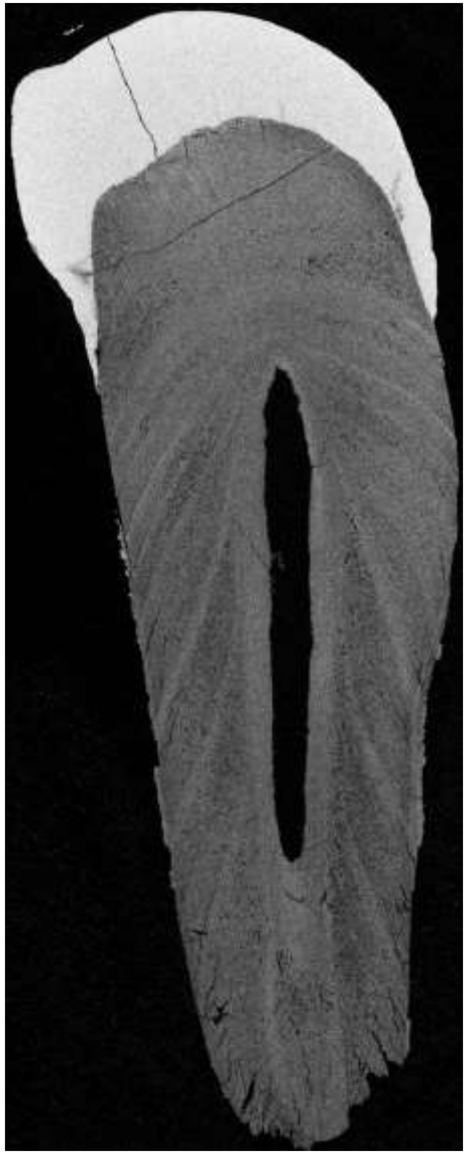

Black: Strontium isotope

Blue: Zinc isotope

Purple: Collagen

Red: Hypoplasia

Note: tooth full of stress

35962

LLM2

Ind. 1

Black: Strontium isotope

Blue: Zinc isotope

Purple: Collagen

Red: Hypoplasia

Green: Caries

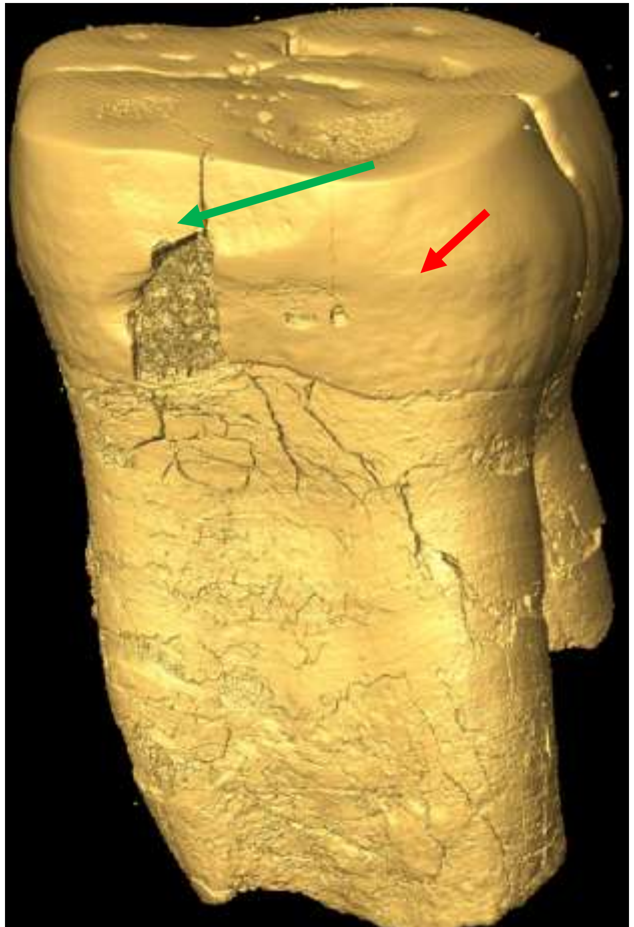

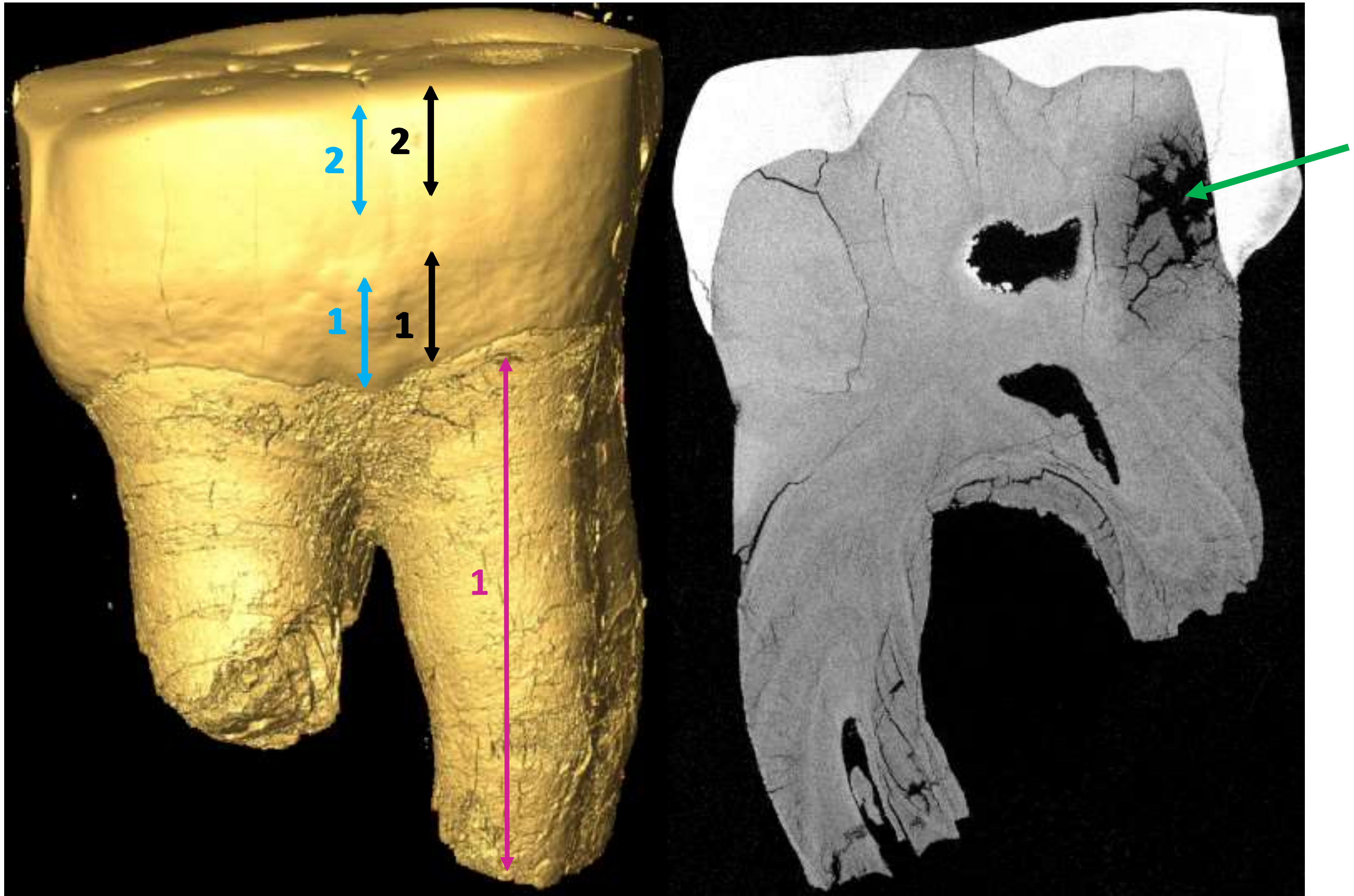

Note: Many small hypoplasia on the root

35959

Ind. unassigned

LxI#

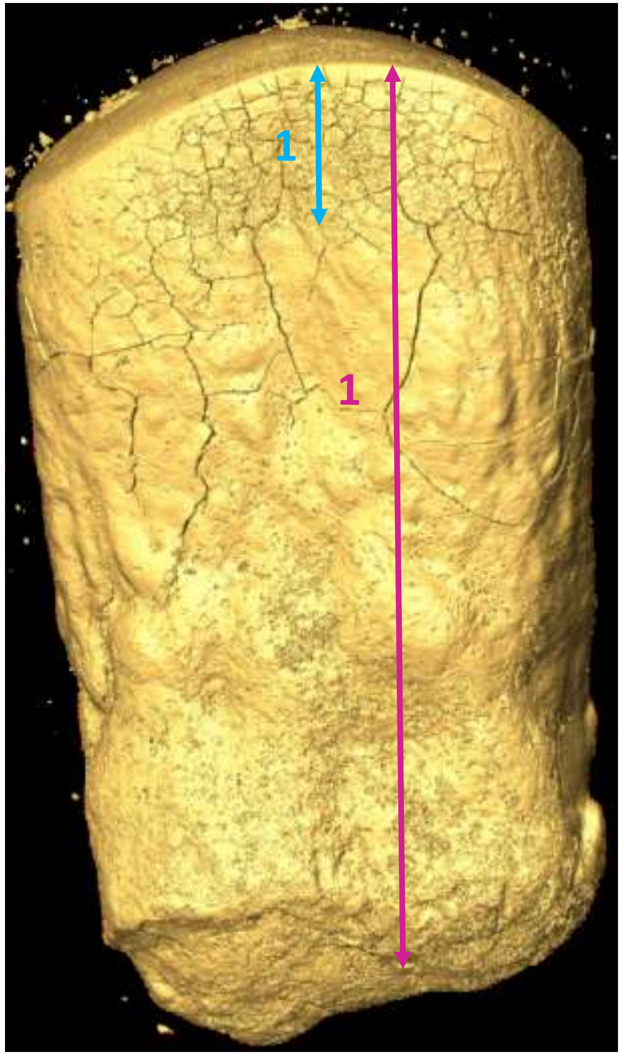

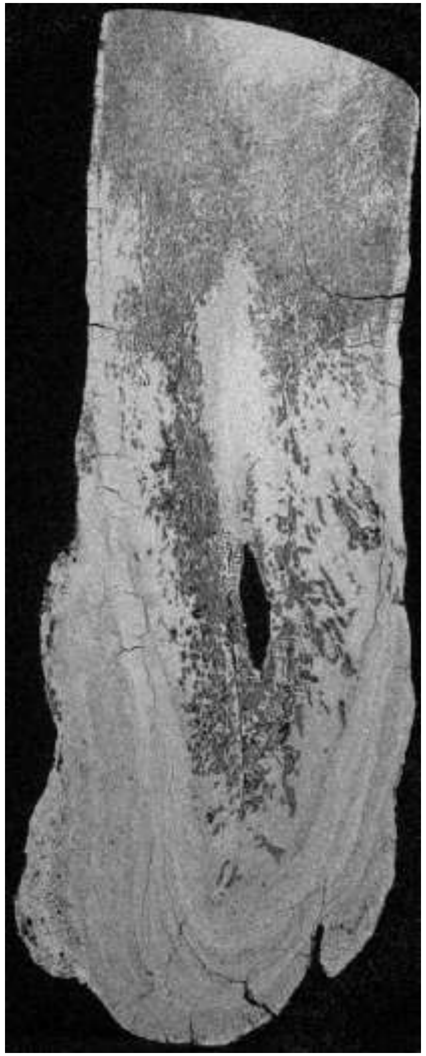

Black: Strontium isotope

Blue: Zinc isotope

Purple: Collagen

Note: stress lines on the cement

35968

unassigned

ULM3

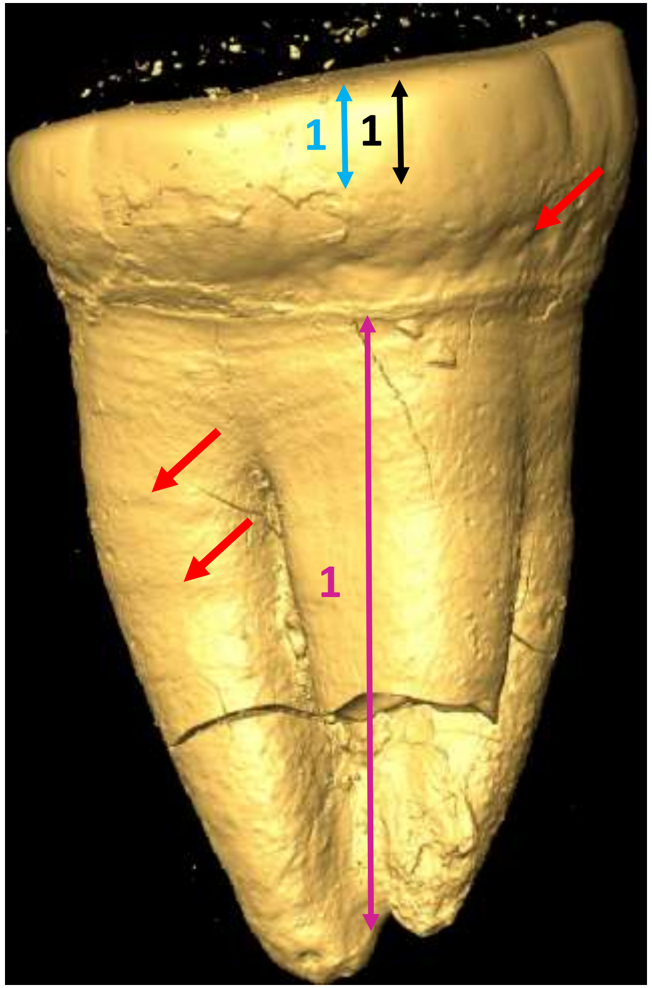

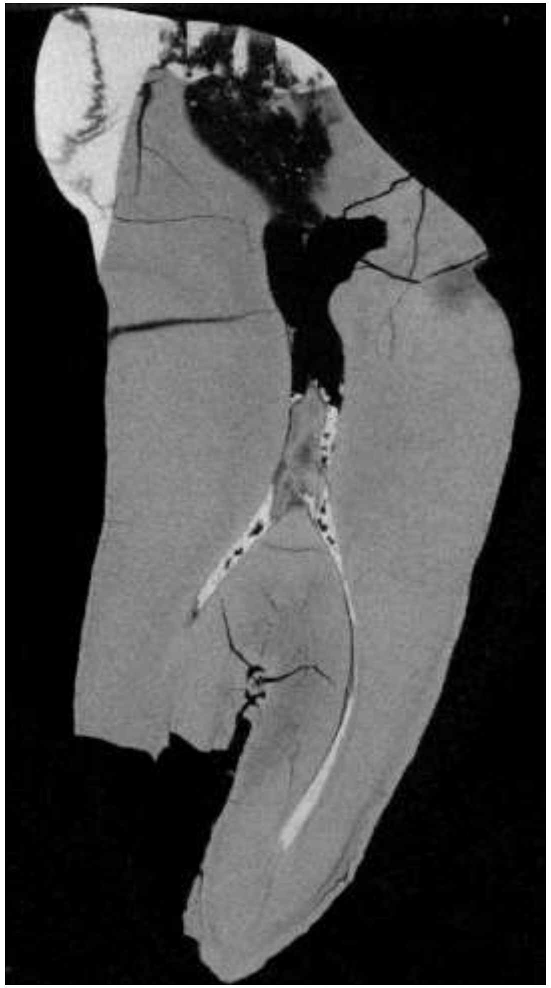

Black: Strontium isotope

Blue: Zinc isotope

Purple: Collagen

Red: Hypoplasia

Note: Very worn tooth

35969

unassigned

LRI2

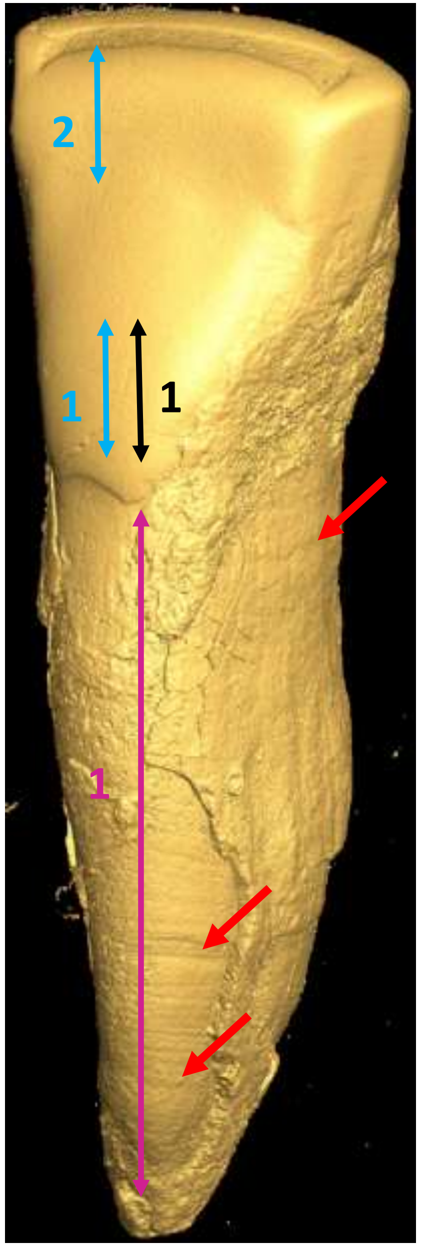

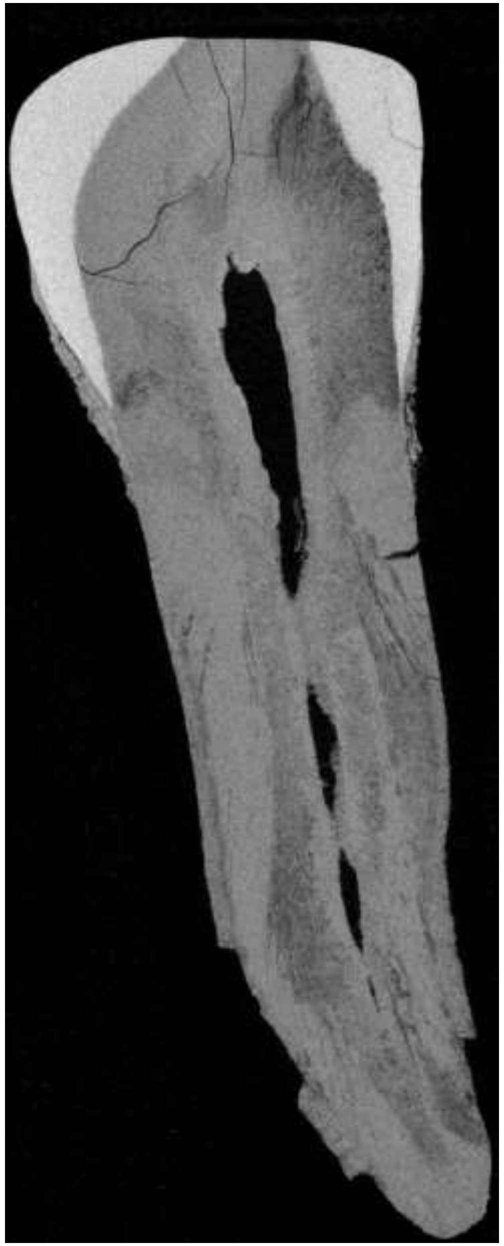

Black: Strontium isotope

Blue: Zinc isotope

Purple: Collagen

Red: Hypoplasia

35970

Ind. 1

UxM#

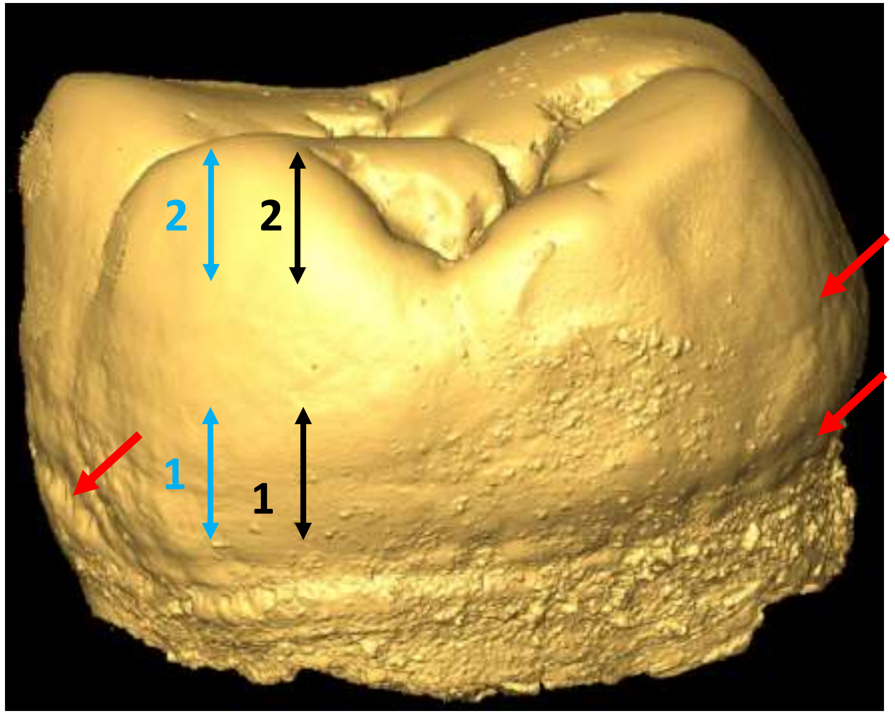

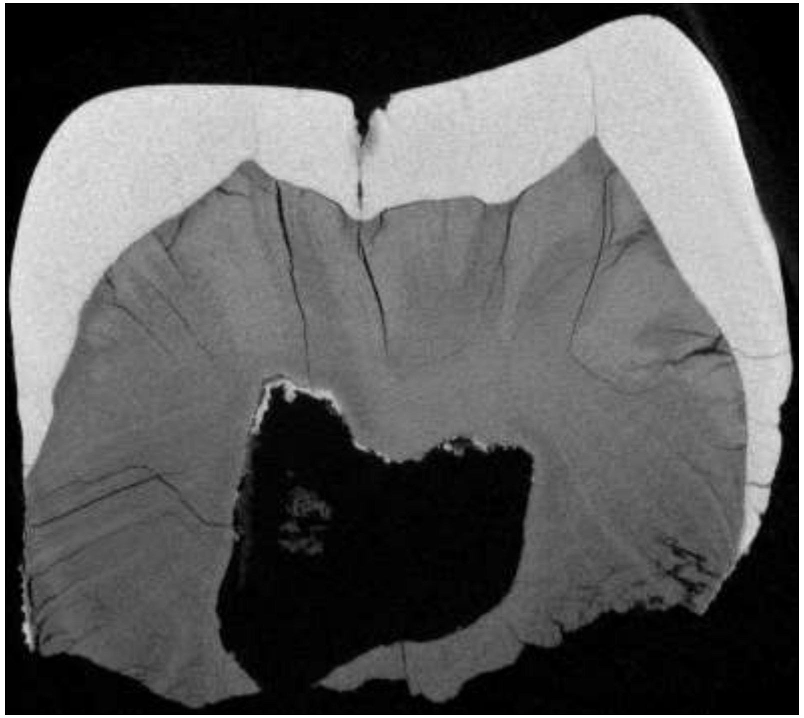

Black: Strontium isotope

Blue: Zinc isotope

Purple: Collagen

Red: Hypoplasia

Note: Tooth full of stress

35967

Ind. 1

LRI2

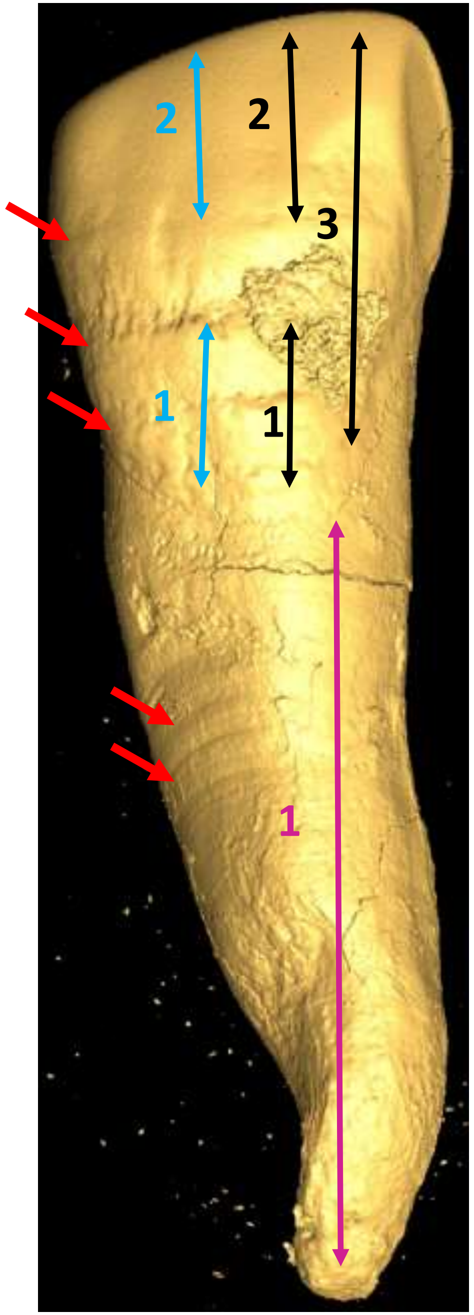

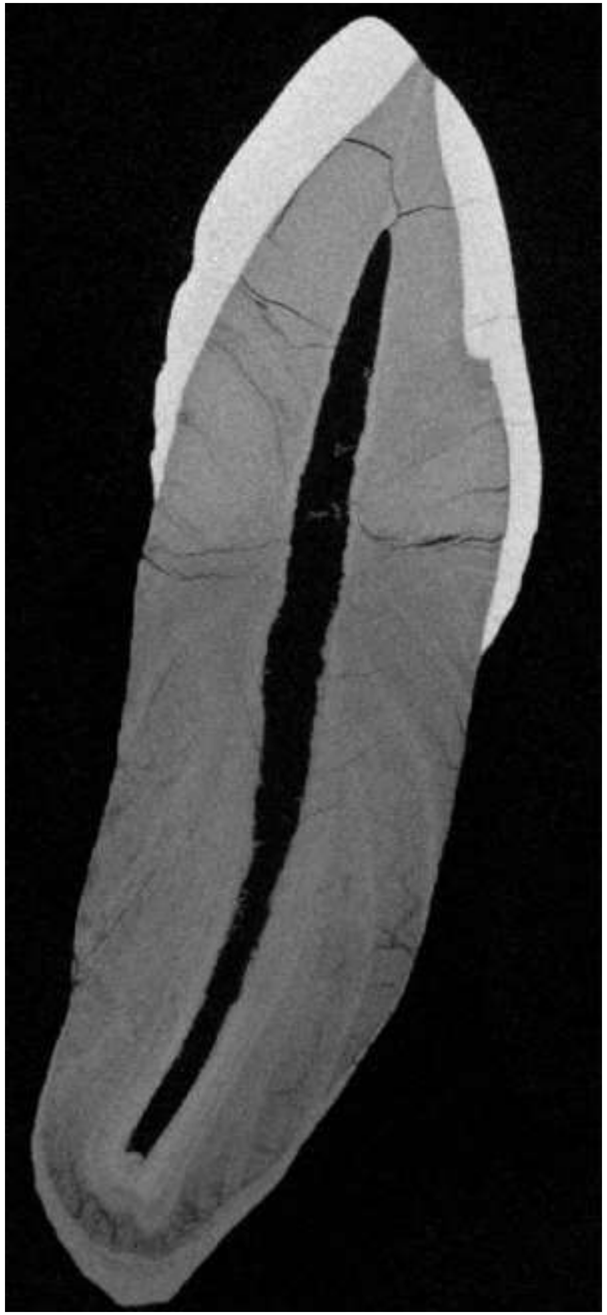

Black: Strontium isotope

Blue: Zinc isotope

Purple: Collagen

Red: Hypoplasia

Note: Tooth full of stress

35971.A

Ind. 5

LLI1

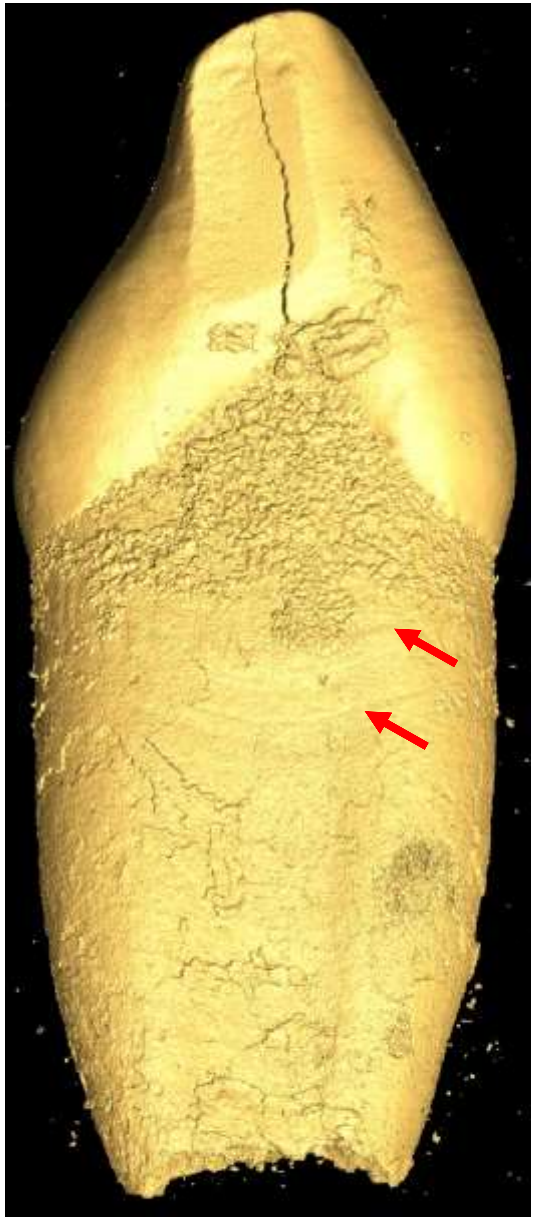

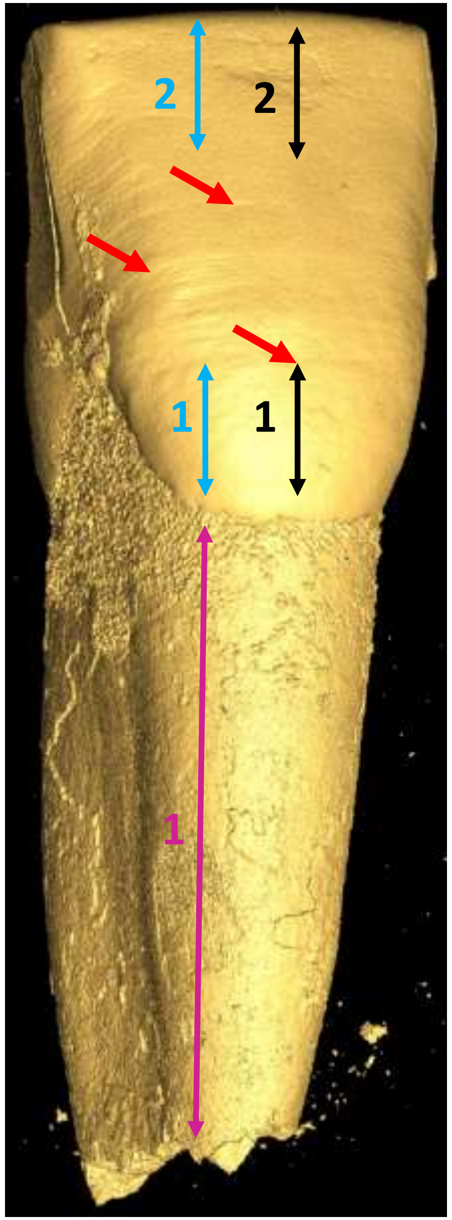

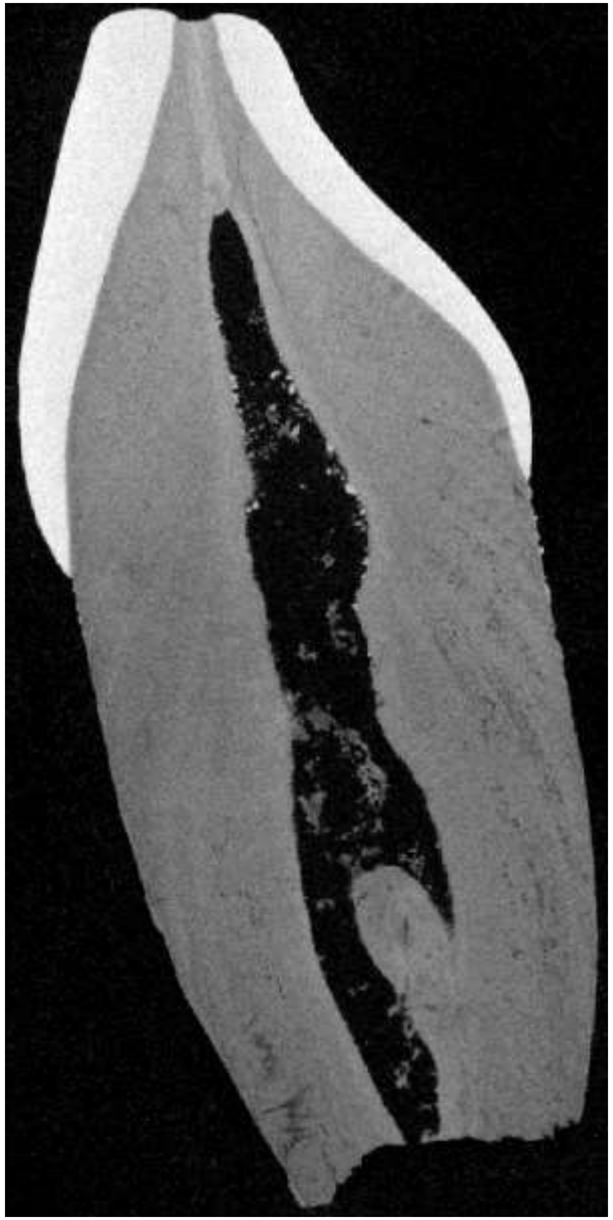

Black: Strontium isotope

Blue: Zinc isotope

Purple: Collagen

Red: Hypoplasia

Note: Tooth full of stress

35971.B

Ind. 5

Black: Strontium isotope

Blue: Zinc isotope

Purple: Collagen

Red: Hypoplasia

Yellow: recrystallisation

LRM1

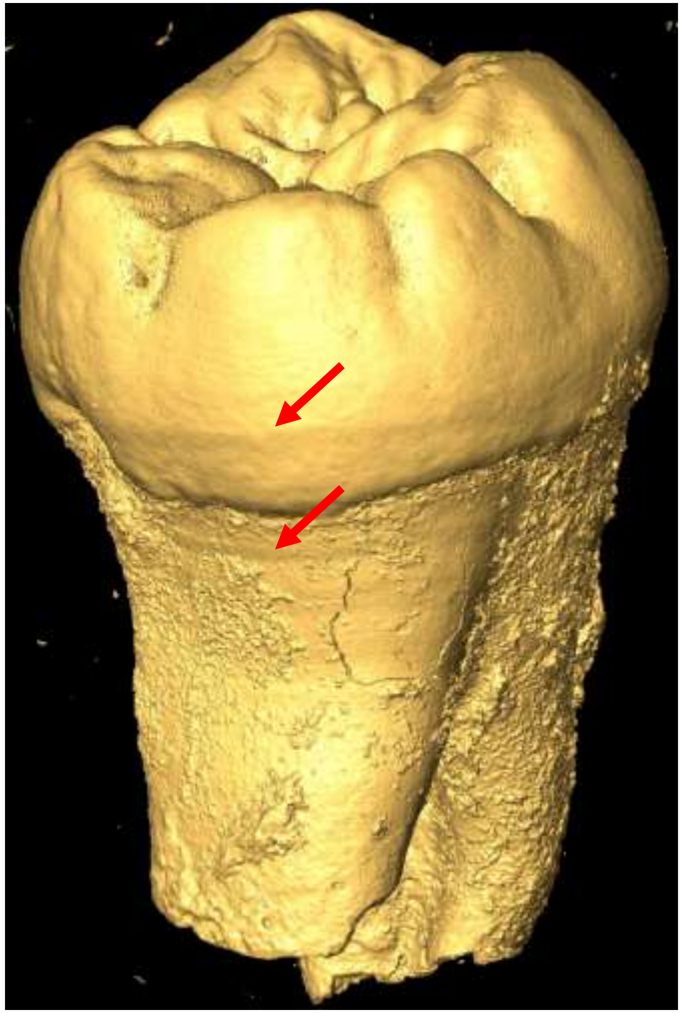

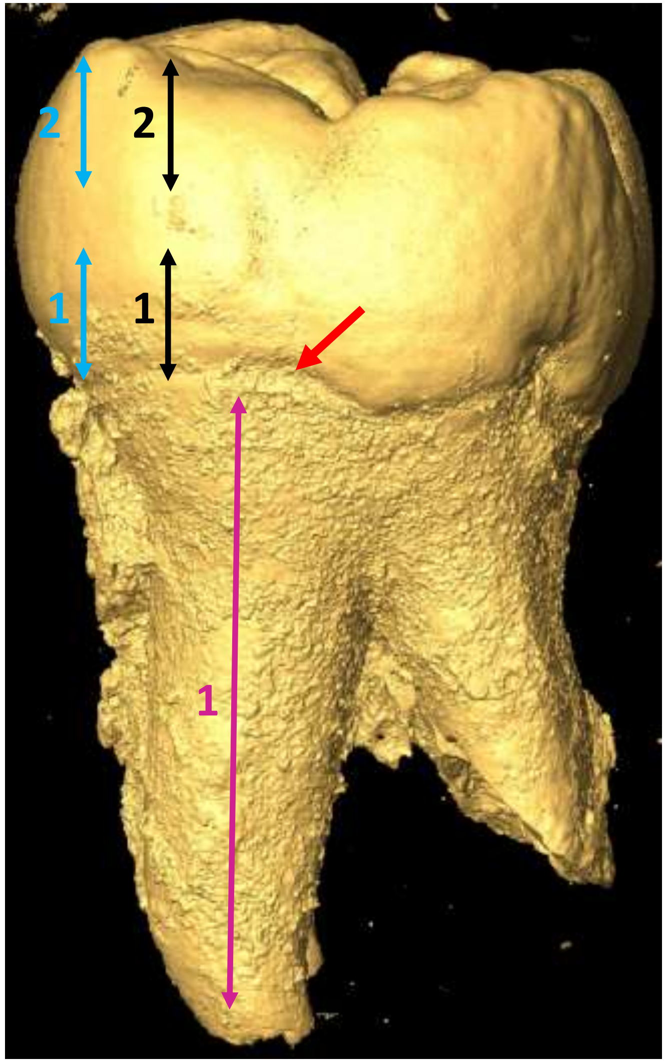

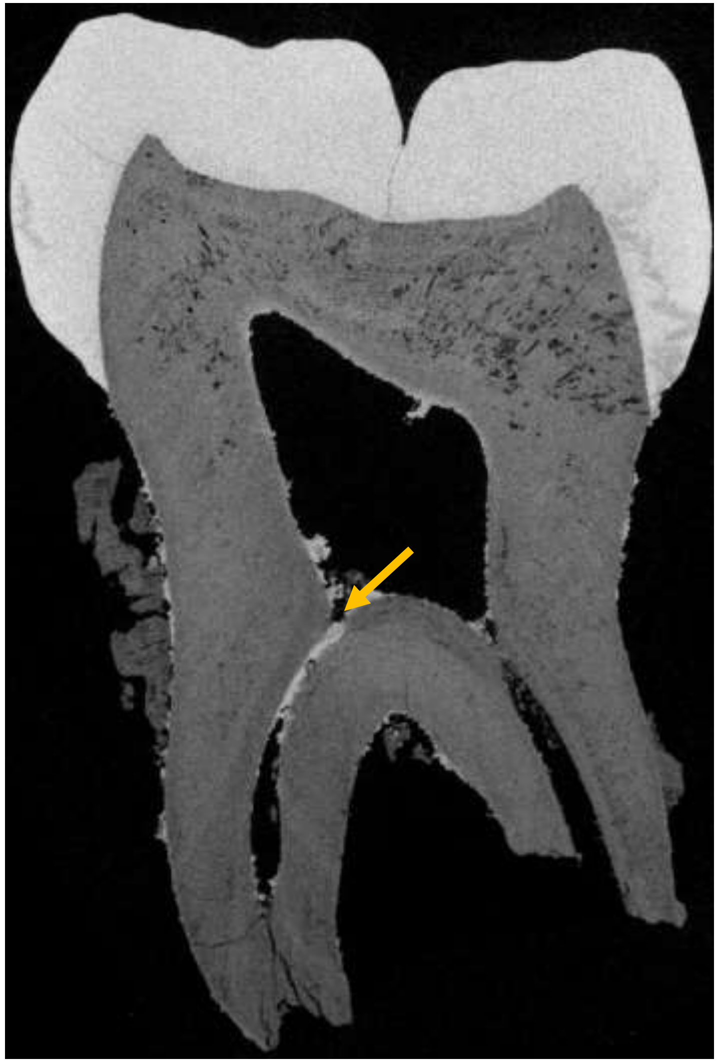

35976

Ind. unassigned

Black: Strontium isotope

Blue: Zinc isotope

Purple: Collagen

Red: Hypoplasia

Green: Caries

LRM#

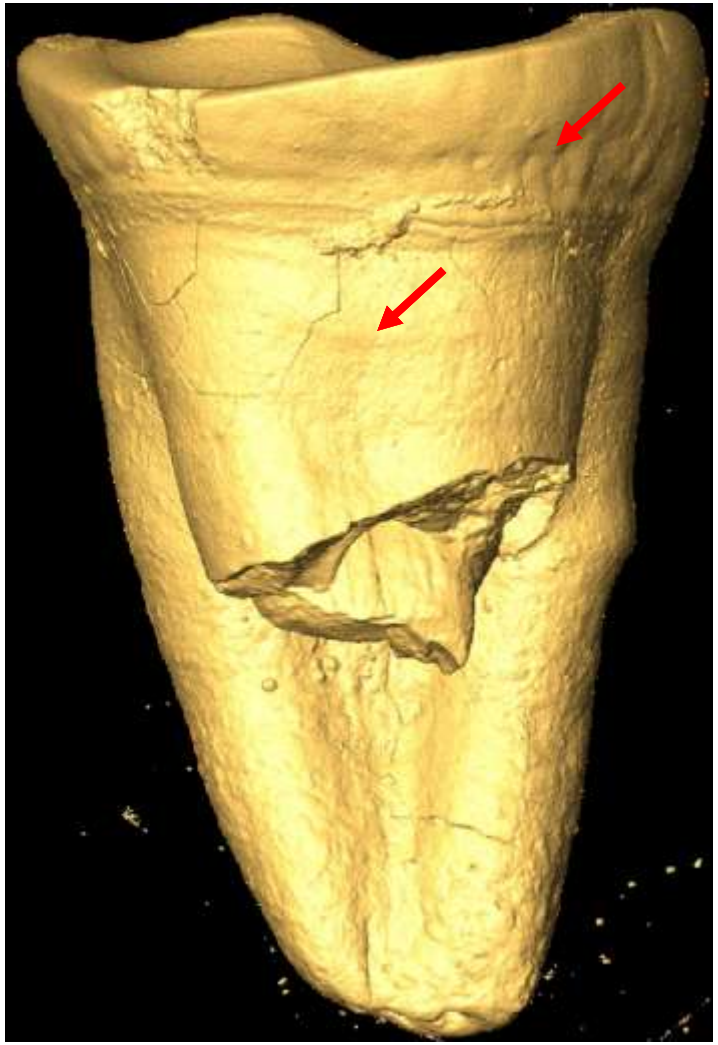

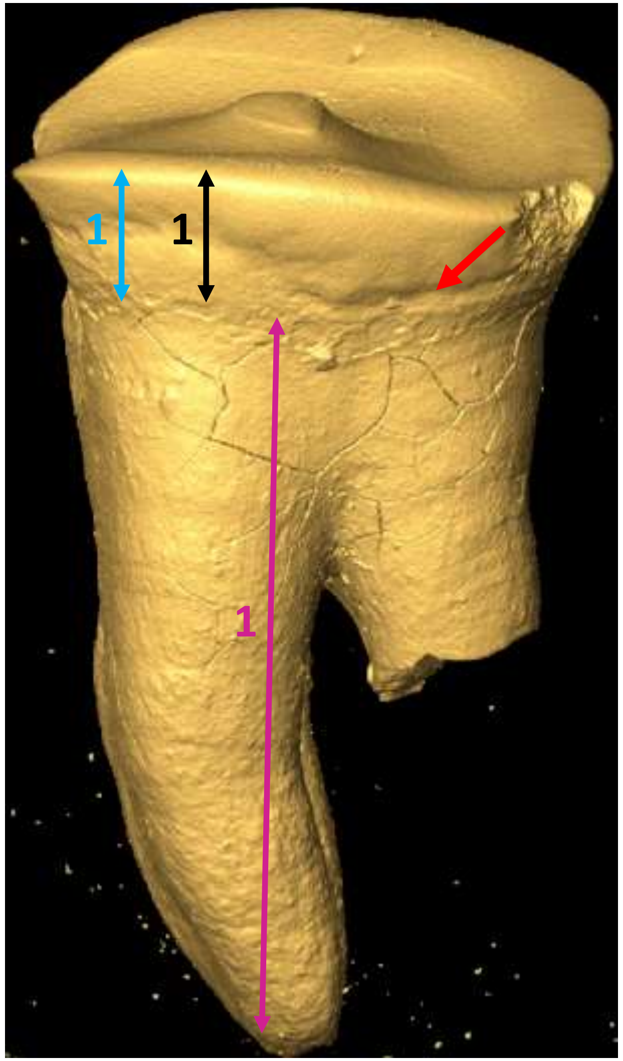

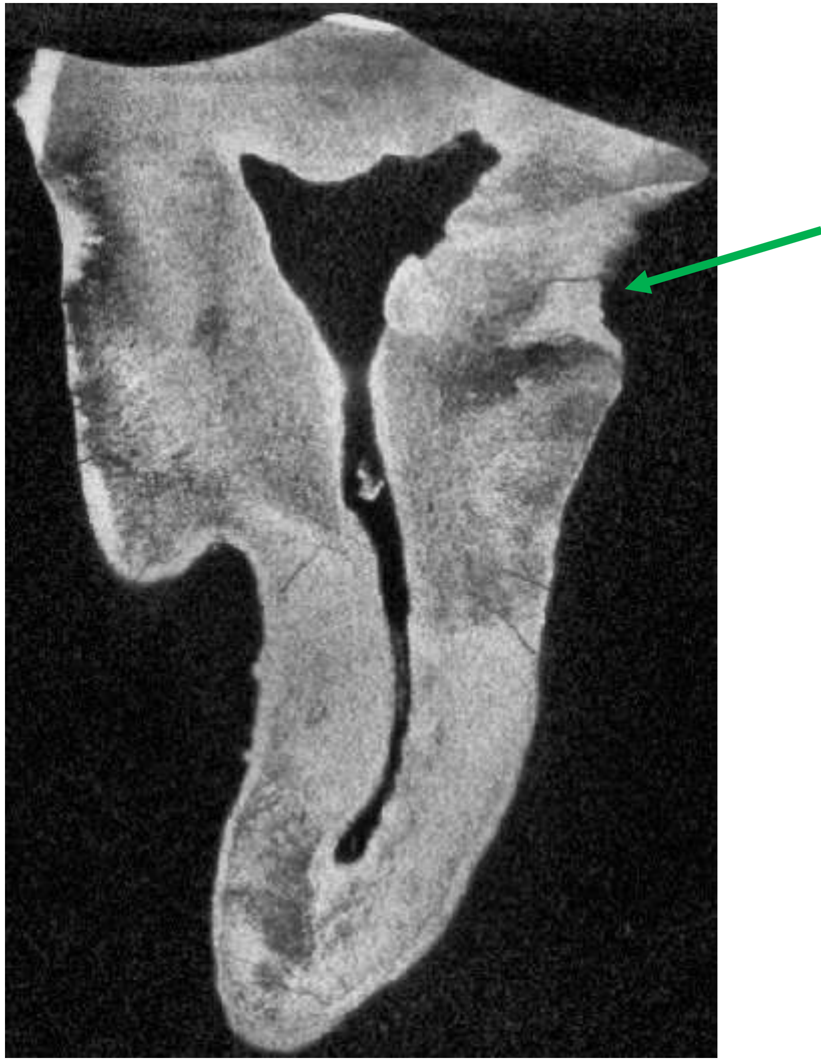

Note: Tooth intensely worn

35972

Ind. unassigned

LLP3

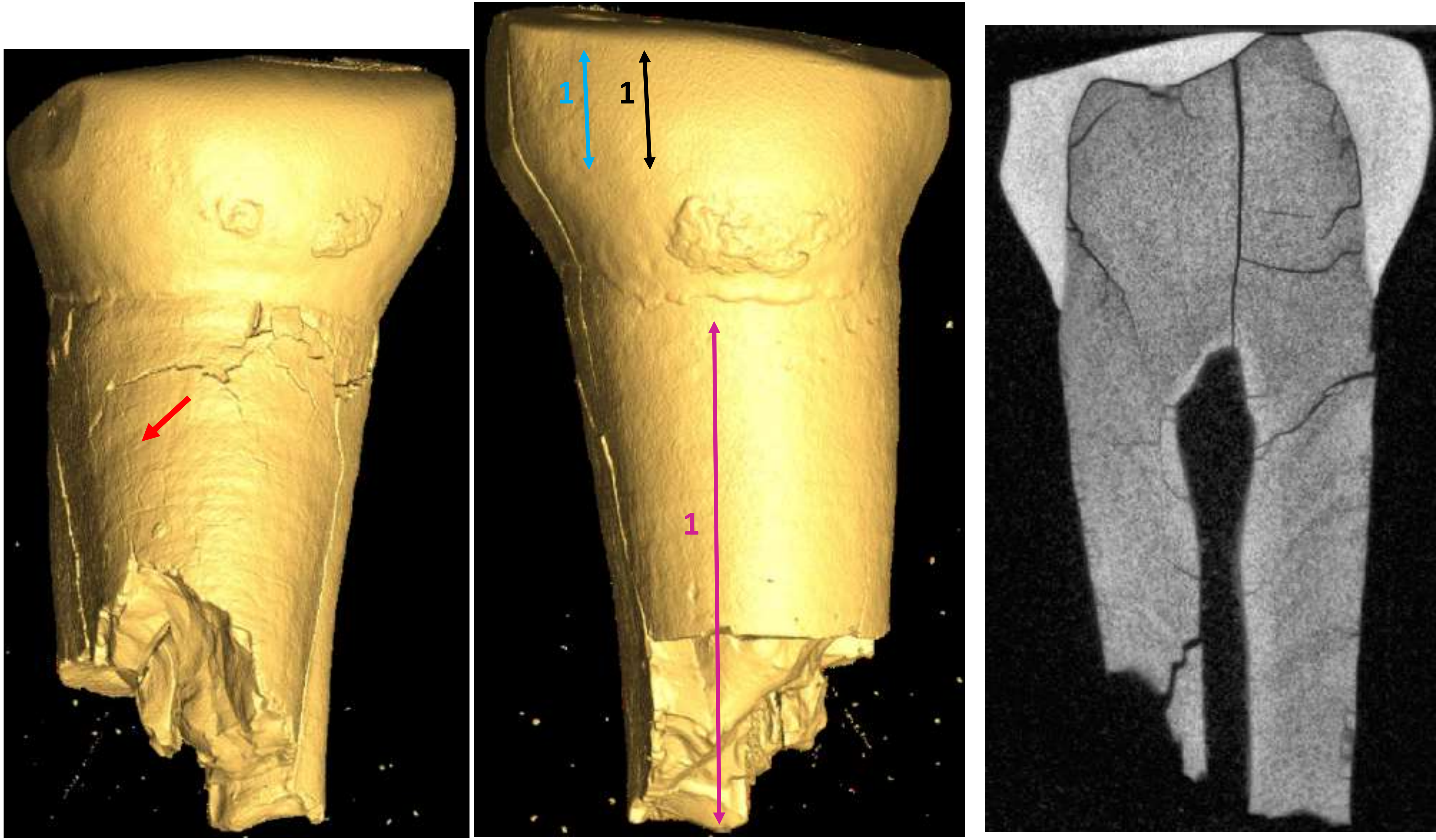

Black: Strontium isotope

Blue: Zinc isotope

Purple: Collagen

Red: Hypoplasia

Note: Many stress on the tooth

35973

Ind. unassigned

LRI2

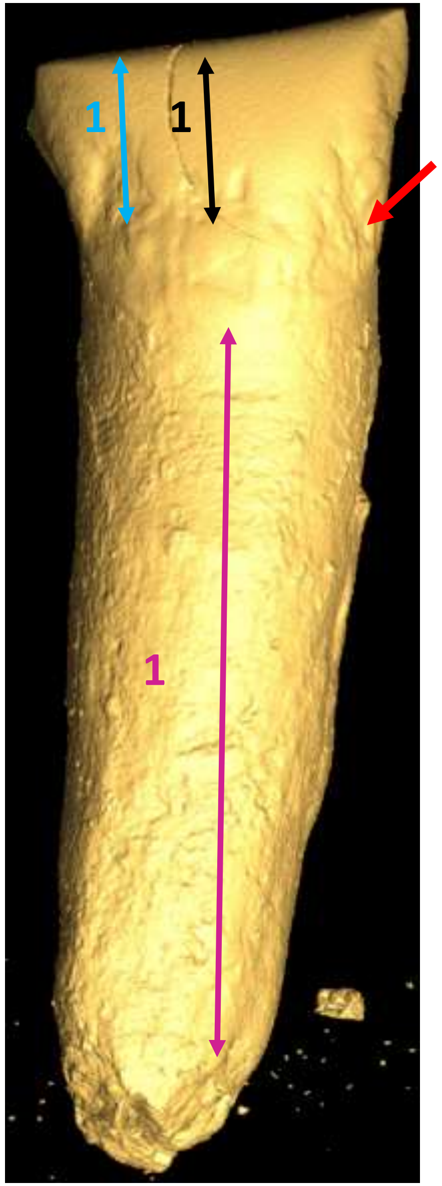

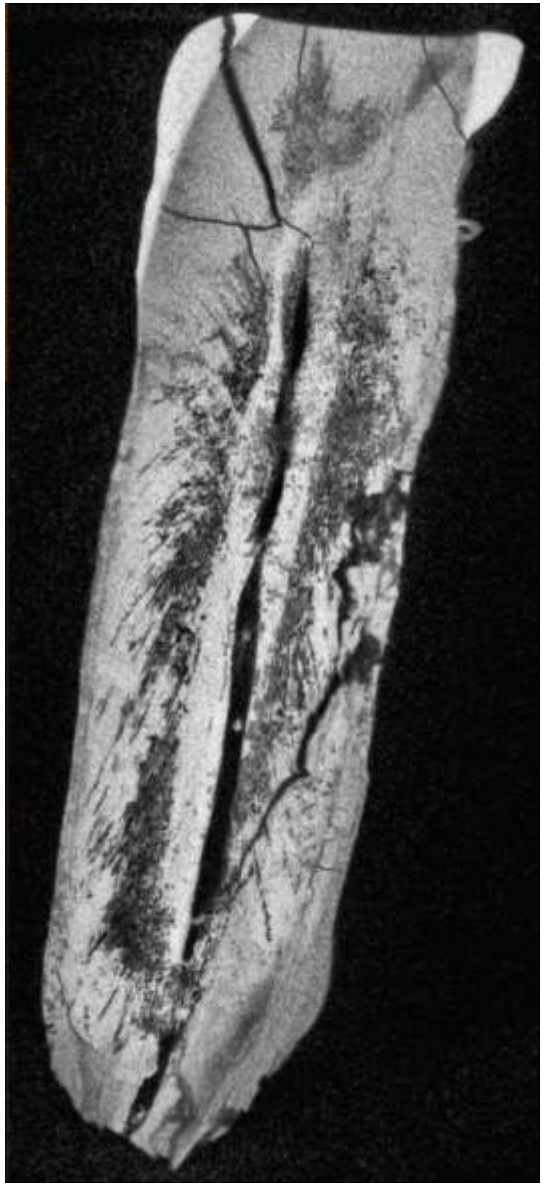

Black: Strontium isotope

Blue: Zinc isotope

Purple: Collagen

Red: Hypoplasia

Note: tooth intensely worn

35979

Ind. 1

LRP4

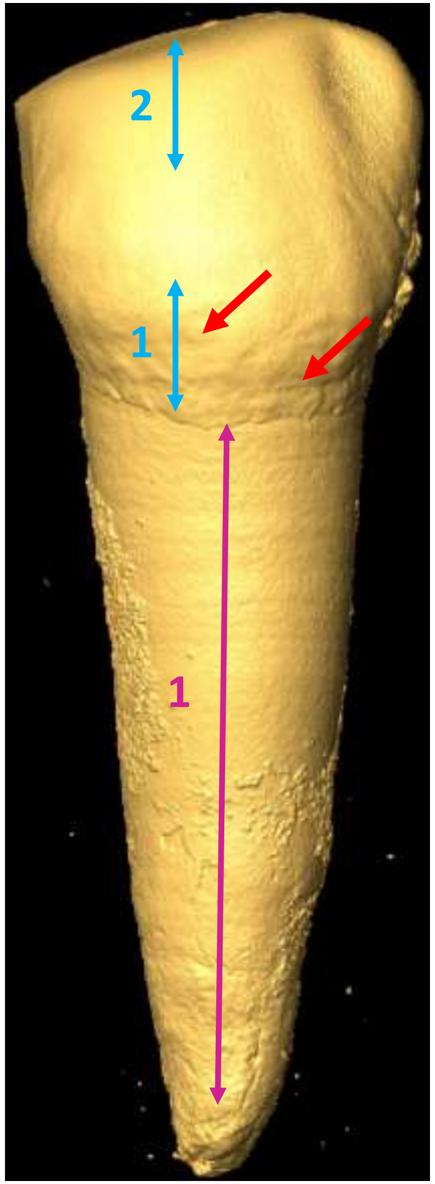

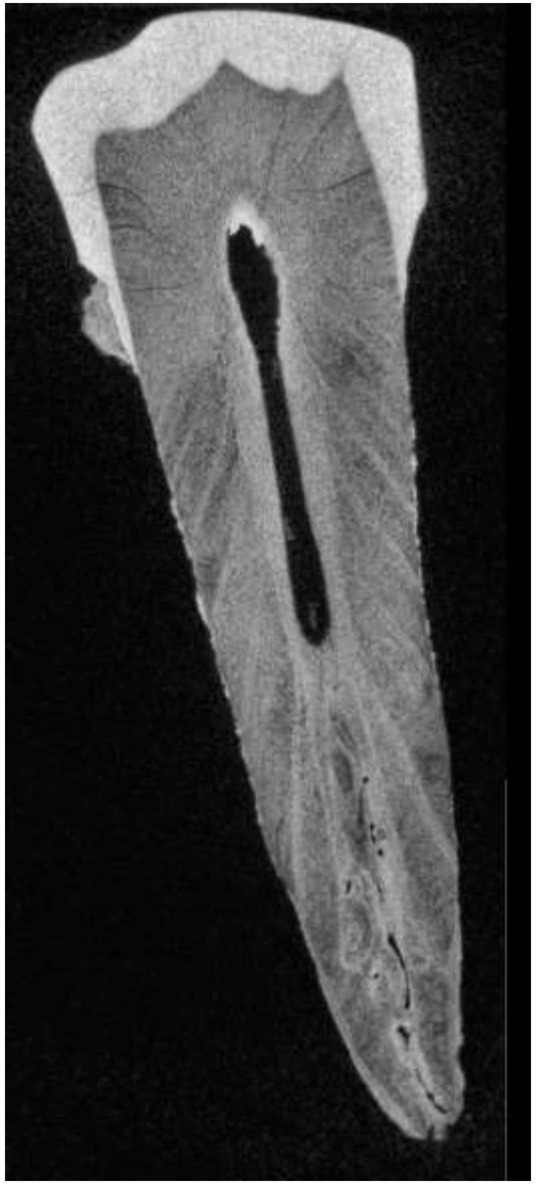

Black: Strontium isotope

Blue: Zinc isotope

Purple: Collagen (Carbon, Nitrogen, CSIA, Sulphur)

Red: Hypoplasia

Note: Many hypoplasia on the root

35977

Ind. 5

ULM2

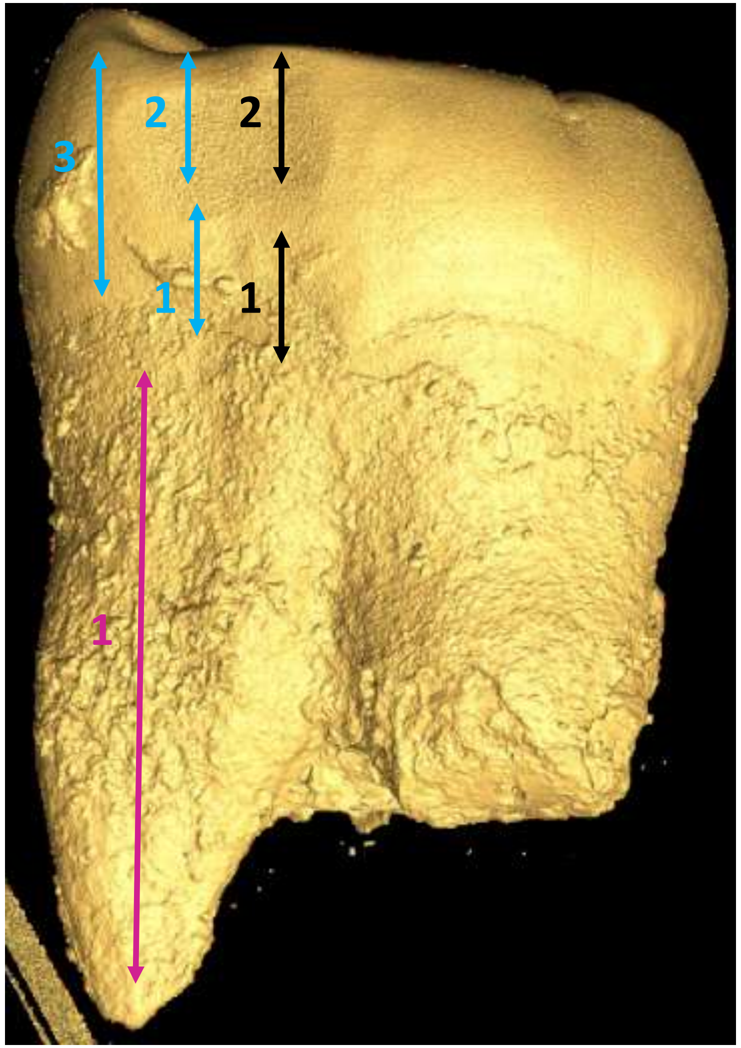

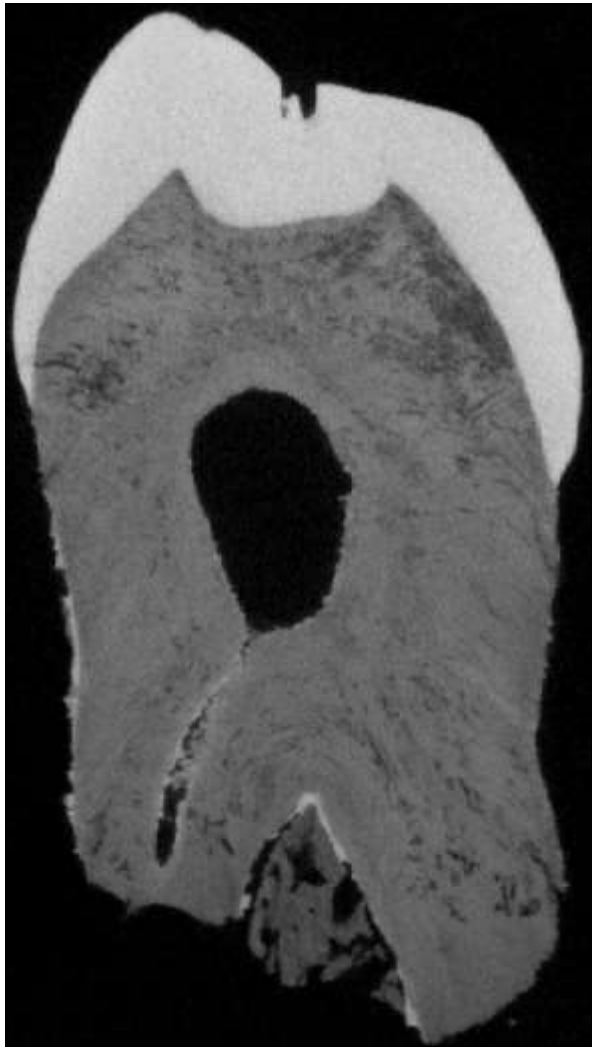

Black: Strontium isotope

Blue: Zinc isotope

Purple: Collagen

35978

Ind. 5

ULC

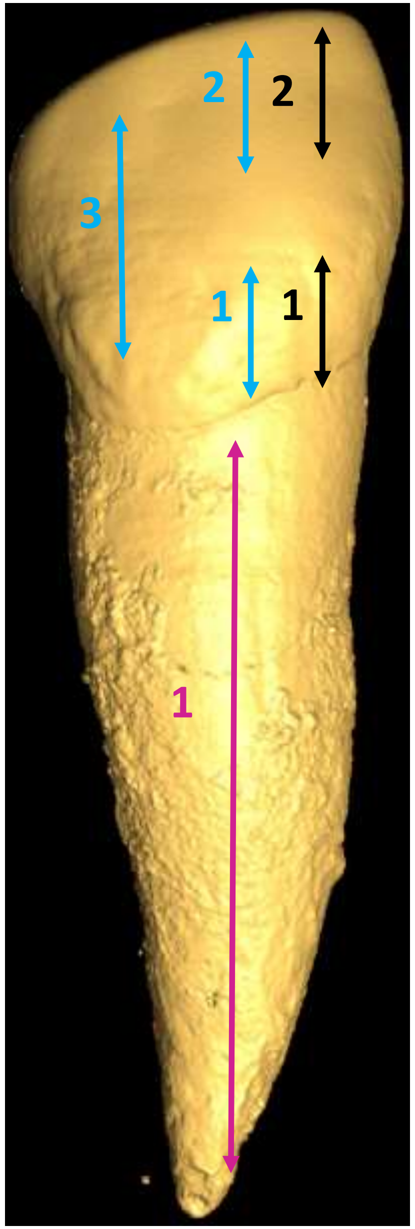

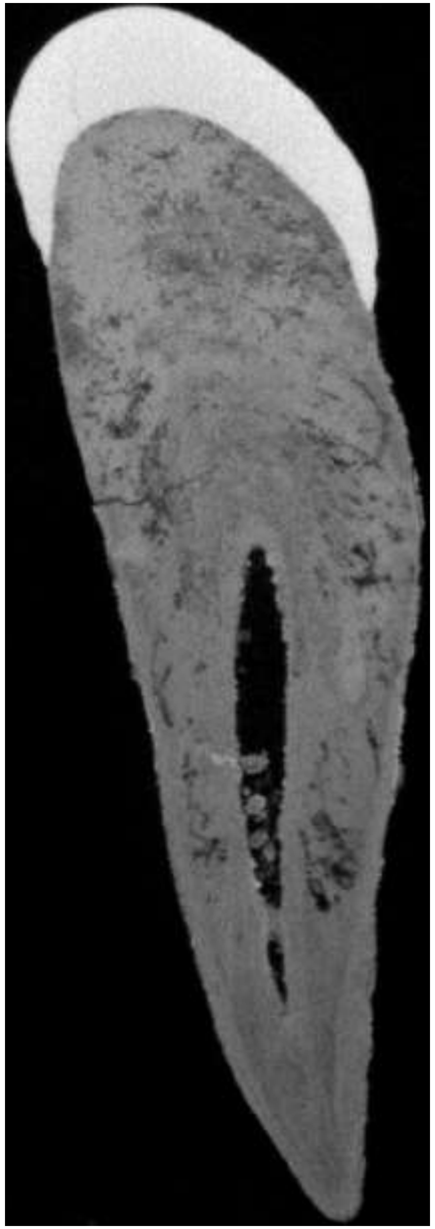

Black: Strontium isotope

Blue: Zinc isotope

Purple: Collagen

SEVA: 35980

Ind. 14

LRP4

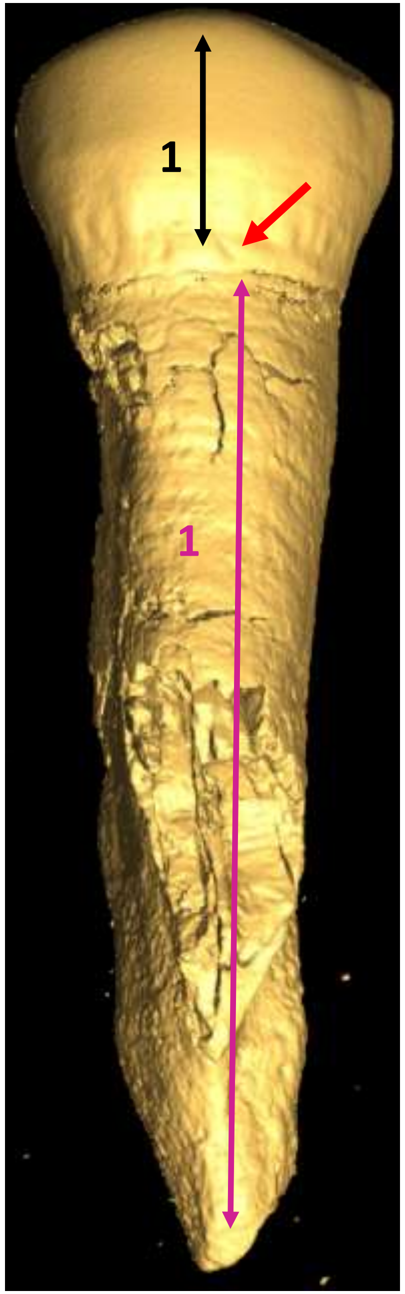

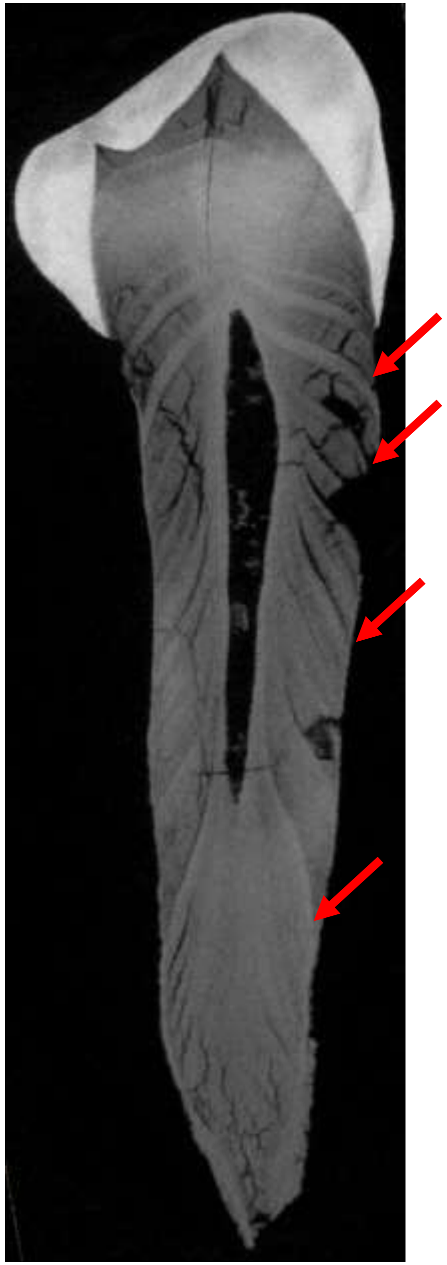

Black: Strontium isotope

Blue: Zinc isotope

Purple: Collagen

Red: Hypoplasia

Note: Intense stress in the tooth.

35974

Ind. unassigned

Black: Strontium isotope

Blue: Zinc isotope

Purple: Collagen

Red: Hypoplasia

Green: Caries

ULM3

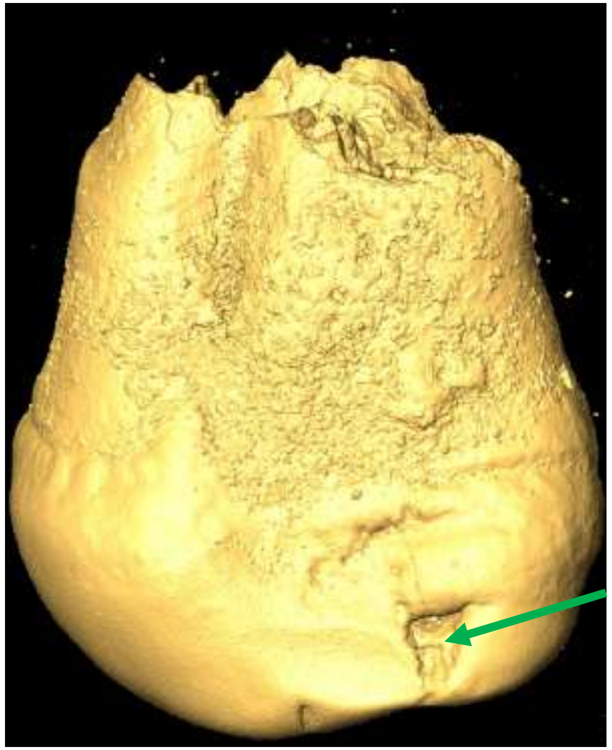

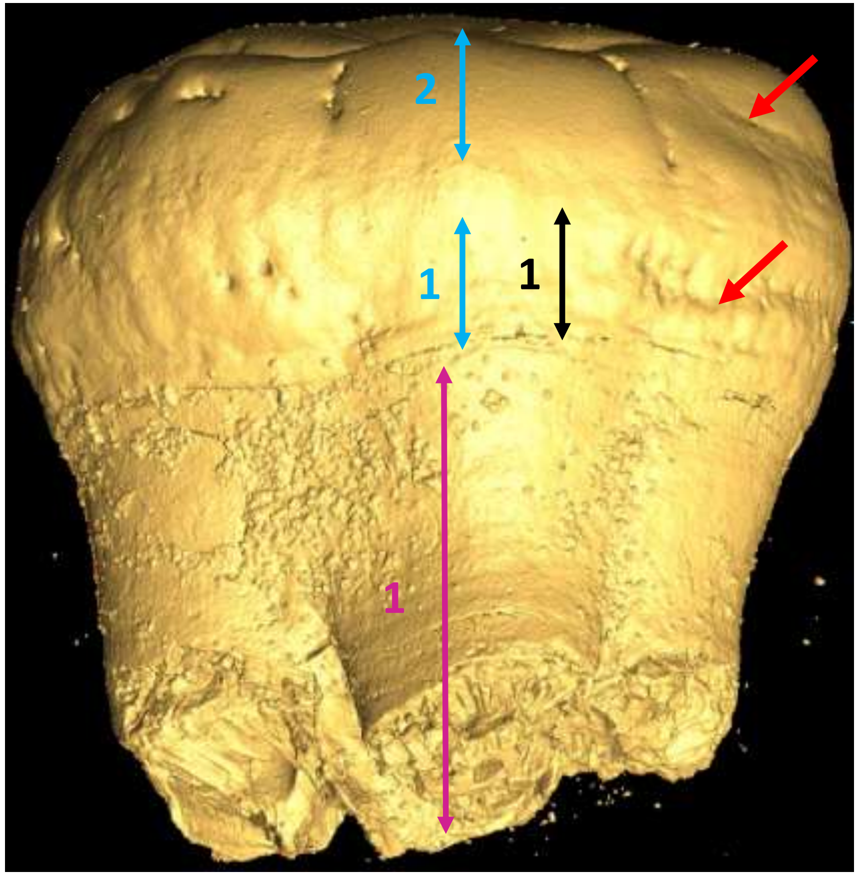

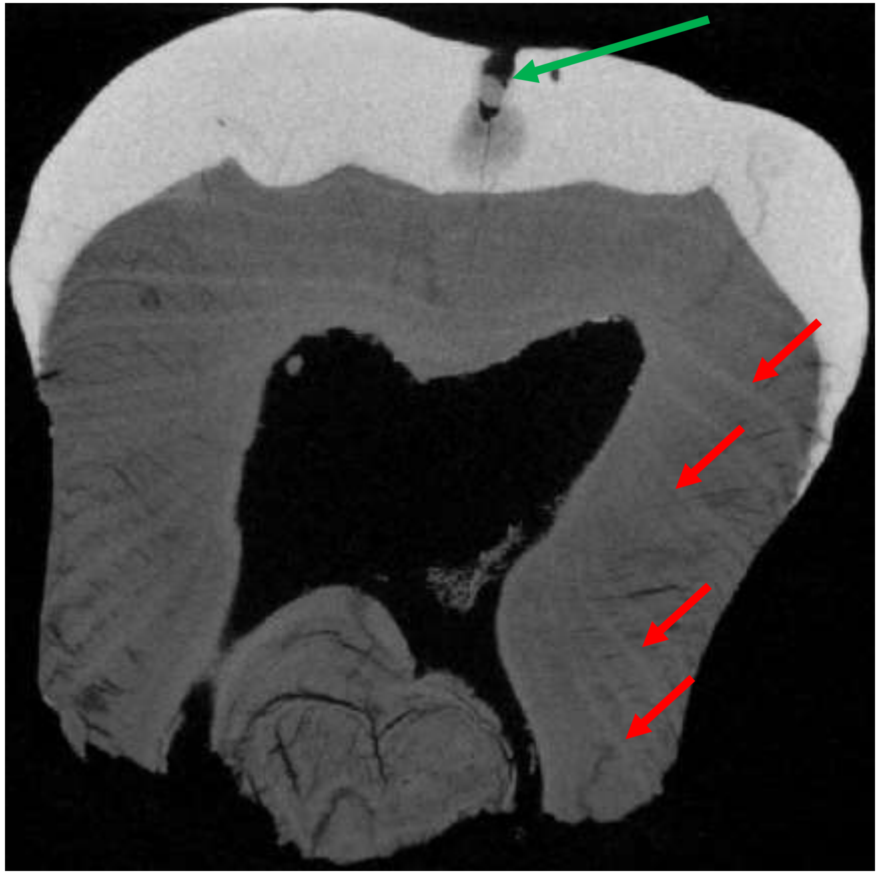

35975

Ind. 13

LRM3

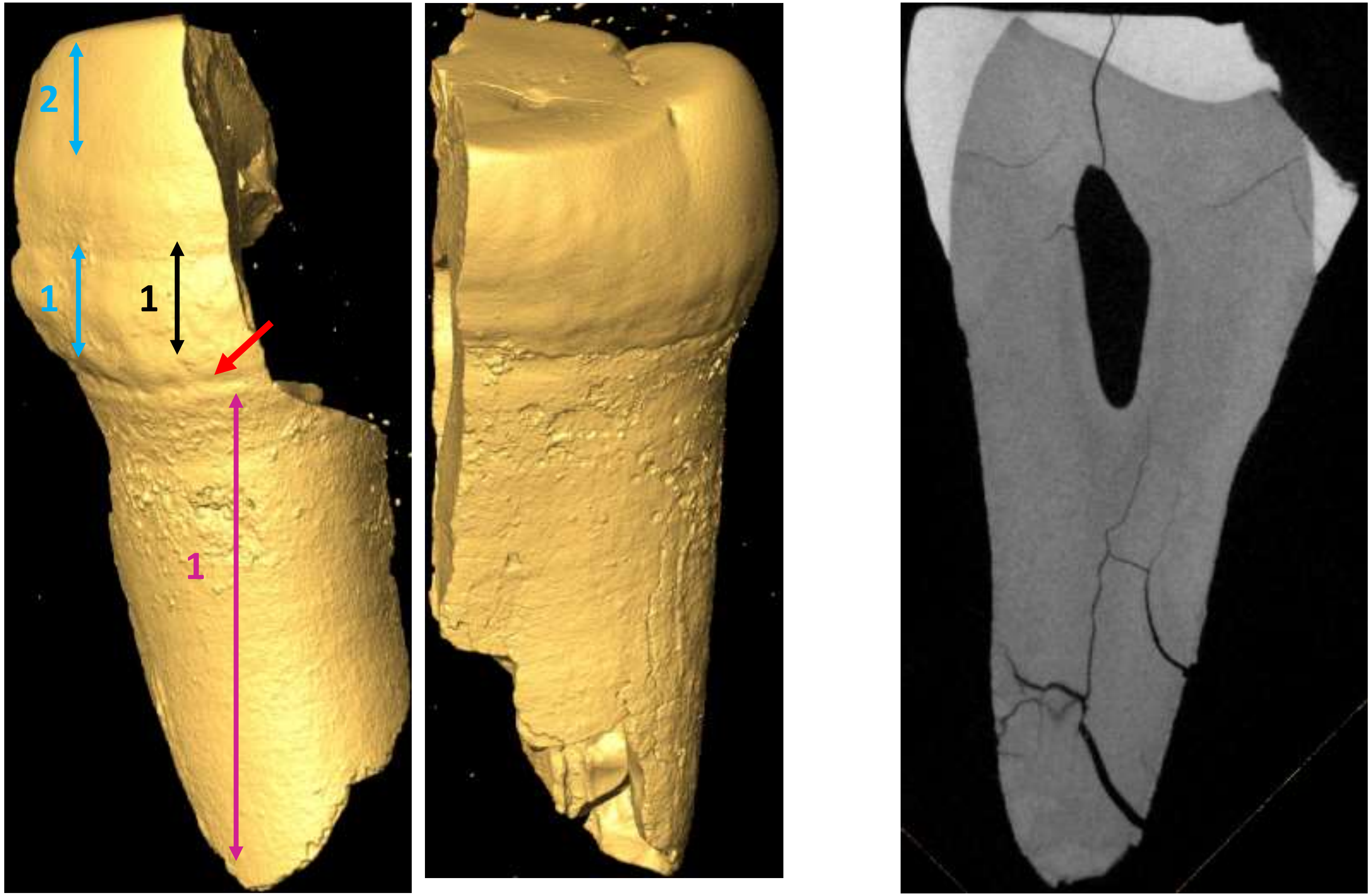

Black: Strontium isotope

Blue: Zinc isotope

Purple: Collagen

Red: Hypoplasia
